# Acknowledgment to Reviewers of *IJMS* in 2021

**DOI:** 10.3390/ijms23031628

**Published:** 2022-01-31

**Authors:** IJMS Editorial Office

**Affiliations:** MDPI AG, St. Alban-Anlage 66, 4052 Basel, Switzerland

Rigorous peer-reviews are the basis of high-quality academic publishing. Thanks to the great efforts of our reviewers, *IJMS* was able to maintain its standards for the high quality of its published papers. Thanks to the contribution of our reviewers, in 2021, the median time to first decision was 16 days and the median time to publication was 34 days. The editors would like to extend their gratitude and recognition to the following reviewers for their precious time and dedication, regardless of whether the papers they reviewed were finally published:

A. GajewskaKatja J. TeerdsA. GanesanKatja WitschasA. Jezela-StanekKatrin BeyerA. N. M. Mamun-Or- RashidKatrin FrauenknechtA. Robles-MarhuendaKatrin S. LipsA. Rod MerrillKatrin SchäferA. V. RakovKatrin Streckfuß-BömekeAalap ChokshiKatrina AlbertAaron F. SeversonKatrina WalshAaron H. MorrisKatsueki OgiwaraAaron MendezKatsuhiko ArigaAaron ReinkeKatsuhiko MorimotoAaron Silva SanchezKatsuhiko MurakiAaron StevensKatsuhiko SuzukiAaron W. MillerKatsuhiro KonnoAashish SoniKatsuki TsuchiyamaAbbas KhoshnoodKatsumi KitagawaAbbas SmileyKatsura TakanoAbd El-Galil E. AmrKatsuro HagiwaraAbdelaziz AmraniKatsuya HiranoAbdeldjalil OuahabiKatsuya HirasakaAbdellah LakehalKatsuyoshi AndoAbdellah TebaniKaushal Kumar BhatiAbdelsamed ElshamyKaushik GhoseAbderrahman MaftahKaveh AhadiAbdou BoucekkineKavindra Kumar KesariAbdul Latif KhanKavita SharmaAbdul RehmanKawa AminAbdul SirajKawachi TetsuyaAbdulhameed Al-GhabkariKawaguchi NanakoAbdullah Al MarufKay D. BeharryAbdullah Sami SaribaşKay Dietrich WagnerAbdussalam AzemKay L. H. WuAbed AgbaryaKay MetcalfeAbel Martínez-RodrigoKay-Dietrich WagnerAbha GuptaKazem M. AzadzoiAbheepsa MishraKazimierz KuliczkowskiAbhigya MookherjeeKazlauskas AndriusAbhijeet A. BakreKazuchika MiyoshiAbhijit AmbegaonkarKazue SembaAbhijit KaleKazuei IgarashiAbhijit SarkarKazuhide OkudaAbhilash SamykuttyKazuhiko ArimaAbhilash VenugopalanKazuhiko KotaniAbhimanyu ThakurKazuhiko YamaguchiAbhinav JainKazuhiro IshibashiAbhinay RamaprasadKazuhiro P. IzawaAbhirup DasKazuhiro YamanoiAbhishek ChandraKazuhisa WatanabeAbhishek DeyKazuhito AsanoAbhishek Kumar SinghKazuhito SuzukiAbhishek MangaonkarKazuki InoueAbhishek ThavamaniKazumasa SakuraiAbid HussainKazumi HiranoAbigail C. LayKazumichi FujiokaAbigail PauleyKazumoto MurataAbolfazl NarmaniKazunari NoharaAbu Bakar SiddiqueKazunori KageyamaAbu Hena Mostafa KamalKazunori KawaguchiAbu Imran BabaKazunori MatsuuraAchchuthan ShanmugasundramKazunori OkadaAchim WernerKazuo ItoAda FunaroKazuo N. WatanabeAdalberto VieyraKazuo NakashimaAdam B. EdwardsKazuomi SatoAdam BoothKazutaka KusunokiAdam BurtonKazuto TajiriAdam C. SedgwickKazutoshi NishijimaAdam C. WilkinsonKazuya DoiAdam D. SteinbrennerKazuya MatsuoAdam DavisKazuyoshi KawaharaAdam E. FramptonKazuyoshi KuwanoAdam FrankelKazuyuki HirookaAdam GołąbKazuyuki TakaiÁdám HorváthKe NingAdam J. LukaszewskiKecheng LeiÁdám JuhászKedar GhimireAdam JunkaKedar PaiAdam KernKee Siang LimAdam LiwoKeechilat PavithranAdam M. PieczonkaKeehoon JungAdam OssowickiKeehoon LeeAdam ReichKeeley L. MacNeillAdam SchrumKee-Lung ChangAdam T. SuttonKee-Yeon KumAdam ThrashKefei ChenAdam Vivian-SmithKei FujiwaraAdamczyk-Grochala JagodaKei HirumaAddolorata CorradoKei IidaAdeel MalikKei MaruyamaAdel ElmoselhiKei NishidaAdél SepsiKeigi FujiwaraAdela BanciuKeigo IkedaAdelaida AvinoKeigo NakamuraAdelaide CaligoKeiichi HironoAdele StewartKeiji HasumiAdeniyi Michael AdebesinKeiji IidaAdhimoolam KarthikeyanKeiji KomatsuAdi BeharKeijo ViiriAdil AkkouchKeiko HosohataAdil MardınoğluKeisuke HitachiAdina RaducanKeisuke OtaniAditya DandekarKeisuke SekineAdler R. DillmanKeita KuroiwaAdnan IsmaielKeita TamuraAdnan KhdairKeitaro HayashiAdnan Shami ShahKeith A. HansenAdnan YounisKeith RochfortAdolfo Baloira VillarKeittisak SuwanAdolfo Gabriele MauroKell OsterlindAdolfo PomaKelvin BrockbankAdoracion CabreraKelvin Ian AfrashtehfarAdoracion Gomez QuirogaKelvin O. YoroAdrià ArboixKen KatoAdrian AugustyniakKen LeungAdrian BarbulKen MillsAdrian Cǎtǎlin PuitelKen MotohashiAdrian ChlandaKen RosenthalAdrian CumminsKen TakahashiAdrian Daniel BoeseKen TakasawaAdrian GajewskiKen TeraoAdrián GarridoKen TeterAdrian Gheorghe MartăuKendrick KooAdrián MatencioKenichi KashimadaAdrian OoKen-ichi KimuraAdrian PiliponskyKen-ichi SatoAdrian Saura-SanmartinKenichi SudaAdrián Tenorio-AlfonsoKen-Ichi WadaAdrian TeoKenichi YoshikawaAdriana E. MieleKen-ichiro MinatoAdriana Estrada-BernalKenji FukunagaAdriana GeisingerKenji IzumiAdriana IsvoranKenji KanaoriAdriana Izquierdo LahuertaKenji MishimaAdriána LiptákováKenji SaitoAdriana PietropaoloKenji SaitohAdriano Costa De CamargoKenji YamadaAdriano LimaKenjiro BandowAdriano MollicaKenneth C. EhrlichAdriano PereiraKenneth D. SwansonAdriano PiattelliKenneth HettieAdrie VerhoevenKenneth HoughAdrienne GormanKenneth HungAdrienne GornyKenneth J. GenoveseAdrienne L. WatsonKenneth W. BerendzenAdrienne ScheckKent BradfordAdrija HajraKenta IwasakiAdugna WoldesemayatKenta MasuiAe-Kyung YiKenta MoritaAfchine FazelKentaro FukushimaAfonso Rocha Martins AlmeidaKentaro NakaminamiÁfrica SanchizKen-Taro SekineAfshin BeheshtiKentaro YamadaAgapi KatakiKento MiuraAgata Daszkowska-GolecKenya KamimuraAgata GabryelskaKenza MamouniAgata GłuszyńskaKenzo KoikeAgata JanowskaKeqiang WuAgata LeszczukKerstin BorgmannAgata MichalakKerstin JurkAgata MossakowskiKerstin MenckAgata PiecuchKerstin TroidlAgata Swiatly-BlaszkiewiczKesen MaAgata SwieciloKeshari ThakaliAgata SzadeKeshav Raj PaudelAgata TyczewskaKetav KulkarniAgata Wawrzkiewicz-JałowieckaKeting ChenAgata ZnamirowskaKeumhan NohAgatha BastidaKeunsub LeeAgenor LimonKeun-Yeong JeongAggelos AvramopoulosKevin A. FenixAglaia PappaKe-Vin ChangAgnès AttardKevin D. PavelkoÁgnes CséplöKevin Rouault-PierreÁgnes CséplőKevin Tak-Pan NgAgnes EnyediKevin Y. YangAgnès FrançoisKfir SharabiAgnes Girard-EgrotKhadija OuguerramAgnès HagègeKhajamohiddin SyedÁgnes KeményKhaled AzizÁgnes KinyóKhaled Mohammed GebaAgnes KlarKhaled Y. KamalAgnes S. KlarKhaleel Ibrahim AssafAgnes SchröderKhalil AhmedAgnes SzepesiKhalil KarimiÁgnes TantosKhalisanni KhalidAgnese De MarioKhayriyyah Mohd HanafiahAgnese MagnaniKheng Siang NgAgnese PoKhosrow KashfiAgnieszka A. Bojarska-JunakKhursheda ParvinAgnieszka A. KaczorKhurshid AhmadAgnieszka AdamekKhushwant Singh BhullarAgnieszka Baranowska-BikKi Hyun NamAgnieszka BiałekKi Soo ParkAgnieszka BlitekKi Wha ChungAgnieszka BossowskaKi Wook KimAgnieszka BronowskaKi Young ChoiAgnieszka BusKian Chuan SiaAgnieszka DacaKiat Hwa ChanAgnieszka Dzikiewicz-KrawczykKi-Bae HongAgnieszka E. DacaKibeom KimAgnieszka FilipekKieran ScottAgnieszka FiszerKi-Ho SonAgnieszka FogelKiichi HirotaAgnieszka GalantyKi-Jong RheeAgnieszka GęgotekKilian SimmetAgnieszka GniazdowskaKim DoughertyAgnieszka GornowiczKim GriggsAgnieszka Graczyk-JarzynkaKim HellgrenAgnieszka HanakaKim Jin-chulAgnieszka Jagiełło-GruszfeldKim M. Van VlietAgnieszka JaniakKimberley WangAgnieszka KarbownikKimberly A. HoffmannAgnieszka Kiełbowicz-MatukKimberly KeilAgnieszka KordekKimberly OngAgnieszka KrupaKimiko InoueAgnieszka Kubicka-TrzskaKiminori OhtaAgnieszka KuchtaKimio FukamiAgnieszka Kulczyńska-PrzybikKimitoshi SakamotoAgnieszka KuźniarKimmo JensenAgnieszka KwiatekKinan AlhallakAgnieszka Lecka-AmbroziakKinga DrzewieckaAgnieszka LobodaKinga GawelAgnieszka LudwikõwKinga Kostrakiewicz-GierałtAgnieszka ŁukomskaKinga SałatAgnieszka Łupicka-SłowikKinnya IshikawaAgnieszka Majkowska-PilipKinsang ChoAgnieszka MalcherKira V. DerkachAgnieszka Marasek-CiolakowskaKira V. VyatkinaAgnieszka MarkowskaKiran Kumar SoniAgnieszka MatuszewskaKiran SapkotaAgnieszka MostowskaKiril MishevAgnieszka Nosal WiercińskaKirill AntonetsAgnieszka Owczarczyk-SaczonekKirill Vitalievich AzarinAgnieszka Paradowska-GoryckaKirk CammarataAgnieszka Piastowska-CiesielskaKirmo WartiovaaraAgnieszka PiegatKirstin VachAgnieszka Piotrowicz-CieślakKishor Kumar SivarajAgnieszka PotęgaKishu RanjanAgnieszka PregowskaKi-Sun KwonAgnieszka RichertKi-Sun LeeAgnieszka RobaszkiewiczKisung KoAgnieszka ScibiorKit Leong CheongAgnieszka SliwinskaKitamura MineakiAgnieszka Strzelecka-KiliszekKivanc GorguluAgnieszka Ulatowska-JarżaKiwoo KimAgnieszka WąsikKiyoharu FukushimaAgnieszka WnukKiyohide IshihataAgnieszka WojtaniaKiyomitsu NemotoAgnieszka ZagórskaKiyoshi EgawaAgnieszka ZawiejskaKiyoshi FujisawaAgnieszka ZienkiewiczKiyoshi MasudaAgnieszka ZyromskaKiyoshi MatsumuraAgostinho LemosKiyoshi OhnumaAgostino GaudioKiyoshi TakagiAgostino GuidaKiyoshi YoshinoAgostino StrangiKiyosumi HoriÁgueda Castro QuintasKiyotaka HitomiAgustín AriñoKiyotaka TokurakuAgustin Gonzalez-VicenteKiyoyuki KitaichiAhan DalalKjetil AndressenAharon AzaguryKlára KosováAhlke HeydemannKlara MegyeriAhmad Fawzi HussainKlas FlärdhAhmad HamdanKlaudia BanachAhmad M. AlqudahKlaudia PawlinaAhmad MirzaKlaus BrandenburgAhmed A. ElolimyKlaus FortscheggerAhmed Abdal DayemKlaus GratzAhmed AbdelghanyKlaus GroschnerAhmed AbdelhakKlaus H. HoffmannAhmed BakillahKlaus HumbeckAhmed EidKlaus Michael HeegAhmed ElaswadKlaus Peter LattéAhmed EltokhiKlaus ReuterAhmed Ezat El ZowalatyKlaus SchickerAhmed FaikKlaus WandeltAhmed HasanKlaus-Dieter SchlüterAhmed HassanKlaus-Peter GötzAhmed M. DebelaKlemen BohincAhmed MaaroufKo FujimoriAhmed Saud AbdulhameedKo OkumuraAhmed Sherif AttiaKocki TomaszAhmet AyazKoen BartholomeeusenAhmet KertmenKoen ReesinkAhmet KrasniqiKoen Van De WeteringAida NaghilouKoen VandenbroeckAida PetcaKohei FunasakaAida SerraKohei HommaAiden EblimitKohei NakamuraAikaterini ArgyropoulouKohei UchimuraAiko NagaiKohei YuyamaAilin WangKohji FukunagaAilyn FadriquelaKoichi FurukawaAimilia Lydia KalafateliKoichi IwataAimin ZhouKoichi KatoAinara CastellanosKoichi KitagawaAinhoa AlberroKoichi KobayashiAinslie Lauren K. Derrick-RobertsKoichi MatsuoAiping ZhangKoichi OgamiAira MatsugakiKoichi TakakiAirat KayumovKoichiro MoriAirton C. MartinsKoji HatanoAisha Siddiqah AhmedKoji MiyamotoAisha SyedaKoji NagataAitor Eguía-BarrioKoji NishimuraAitor MaestroKoji OnomotoAjay AshokKoji OtaniAjay KumarKoji SawadaAjay Kumar YagatiKok Yong ChinAjay PalKo-Lin KuoAjay SharmaKollarigowda RavichandranAjinkya KawaleKolowerzo-Lubnau AgnieszkaAjit MagadumKomal KalaniAjjampura C. VinayakaKomuraiah MyakalaAkanksha SharmaKonda Reddy KarnatiAkanksha SinghKondababu KurakulaAkbar NawabKondareddy CherukulaAkhalesh Kumar ShakyaKoni GrobAkhilesh RaiKonno MasaeAkifumi KushiyamaKonrad A. SzychowskiAkihiko MiyanagaKonrad HuppiAkihiko NishikimiKonrad SzustakiewiczAkihiko OkaKonstantin A. LukyanovAkihiko TakadaKonstantin A. ProsolovAkihiko WatanabeKonstantin BelosludtsevAkihiro IsozakiKonstantin BirukovAkihiro MatsumotoKonstantin ChekanovAkihito TsubotaKonstantin KozlovAkikazu FujitaKonstantin MineevAkiko ArataKonstantin MiroshnikovAkiko Kojima-YuasaKonstantin PikulaAkiko KozakiKonstantin V. AnokhinAkiko MaedaKonstantin V. KiselevAkiko Sakasai-SakaiKonstantin V. MoiseenkoAkila MayedaKonstantina KaramanouAkilan KrishnamurthyKonstantinos AvgerinosAkimasa MiyanagaKonstantinos DimasAkinari SawadaKonstantinos KarakostisAkinobu NakamuraKonstantinos KoudounasAkinobu UsamiKonstantinos MakrisAkinori KawakamiKonstantinos SapalidisAkinori KibaKonstantinos SousounisAkio MiyaoKonstantinos VavitsasAkira IguchiKoon Ho ChanAkira KakugoKoraljka Gall TroseljAkira KitamuraKoraljka HusnjakAkira MatsudaKormos ViktóriaAkira MonjiKornél KirályAkira NishiyamaKornelia JaquetAkira SekikawaKorsi DumenyoAkira ShinoharaKoshiro NishimotoAkira SugawaraKosma SzutkowskiAkira TakedaKosmas DaskalakisAkira YamaguchiKostantin ChegaevAkira YamauchiKostas PantopoulosAkishige HokugoKosuke NishiAkito TanedaKosuke OkuyaAkiyoshi KawaokaKosuke SakoÁkos HorváthKota ArimaÁkos M. LőrinczKotakonda ArunasriAkos VegvariKotapati Kasi ViswanathAkshara RaghavendraKotb AbdelmohsenAkshata NaikKouichi IwataAkshay KumarKouichiro KawanoAkshay PandeyKoumei BabaAkshaya ChandrasekaranKousik SundararajanAkshyalakshmi SridharKousuke TsuchiyaAlaa AdawyKovalik Jean-PaulAlagu ManickaveluKowichi JimbowAlain ChapelKrešimir GalešićAlain CouvineauKrešimir RotimAlain HendliszKris A. CampbellAlain LabroKrish ChandrasekaranAlain LescureKrishan KumarAlain-Pierre GadeauKrishan RaiAlak MannaKrishna BhattaraiAlan A. ChenKrishna C. ChintaAlan B. RoseKrishna Chaitanya PavaniAlan DiamondKrishna ChinthalapudiAlan H. DeCherneyKrishna Mohan Padmanabha DasAlan HarperKrishna Mohan SurapaneniAlan HerbertKrishnanand P. KulkarniAlan J. WhitmarshKrishnarjuna BankalaAlan ReinKrishnendu KhanAlana ThackrayKrishnendu PalAlarico ArianiKrista M. HeinonenAlba GarciaKrista RomboutsAlba Maiques-DiazKristaps JaudzemsAlban GiraultKristel SleegersAlbano HelenaKristen VarneyAlbano MeliKristiaan J. Van Der GaagAlbena StoyanovaKristian Bruun LaursenAlbert E. PattersonKristian HaanesAlbert GuskovKristiina HimanenAlbert MoralesKristiina HuttunenAlbert RubbenKristiina M. NordforsAlbert Wilhelm SchulthessKristin WattAlberto AimoKristina KantminienėAlberto BañosKristina KuhbandnerAlberto Canfran-DuqueKristina LorenzAlberto CuestaKristina RamanauskienėAlberto Dávalos HerreraKristina ViktorssonAlberto FerrareseKristine FreudeAlberto GianinettiKristine M. KrajnakAlberto GranzottoKristine McGrathAlberto IngaKristof De SchutterAlberto LapiniKristóf Endre KárolyAlberto LeoniKristofer AndréassonAlberto Lo GulloKristy SwiderskiAlberto Marin-SanguinoKrisztina BelaAlberto Martín-MolinaKrisztina LudányiAlberto MiceliKrisztina PohóczkyAlberto OuroKrisztina TarAlberto Pérez-MediavillaKrupa NavalkarAlberto Rodríguez-ArchillaKrupal P. JethavaAlberto SensiniKrutika PatelAlberto TommasiniKrystyna MichalakAlbino CarrizzoKrystyna Mojsiewicz-PieńkowskaAlbrecht ClementKrystyna OraczAldo BonaventuraKrystyna Pierzchała-KoziecAldo GaleoneKrystyna WiniarczykAldo NicosiaKrzesimir CiuraAldo RoccaroKrzysztof BryniarskiAldona KasprzakKrzysztof BrzezińskiAleix SolanesKrzysztof CzajaAlejandra TomasKrzysztof CzamaraAlejandro A. TapiaKrzysztof FlisikowskiAlejandro BernaKrzysztof FornalskiAlejandro BugarinKrzysztof J. KurzydłowskiAlejandro Cabezas-CruzKrzysztof J. PawlikAlejandro Iglesias-LinaresKrzysztof KaliszewskiAlejandro Iván Ruiz RiquelmeKrzysztof M. MrozikAlejandro Molina LeyvaKrzysztof MarciniecAlejandro Rodriguez-SanchezKrzysztof PolewskiAlejandro RomeroKrzysztof SztankeAlejandro Samhan-AriasKrzysztof SzyfterAlejandro SanzKrzysztof TrederAleksandar M. JeremicKrzysztof ŻamojćAleksandar MilosavljevicKrzysztof ZienkiewiczAleksandar PavicKrzysztof ZwierzAleksander BurilovKsenia EgorovaAleksander F. SikorskiKsenia KirichenkoAleksander FilarowskiKsenija DurgoAleksander SiniarskiKshitij KhatriAleksander ZidanšekKuang-Hung ChengAleksandr A. RubelKuang-Sheng YehAleksandr KazachenkoKuang-Wen TsengAleksandra AdamskaKuan-Ting LinAleksandra Bielawska-PohlKuan-Wei ChenAleksandra BorodkinaKubra AralAleksandra CzumajKuei-Yang HsiaoAleksandra Duda-ChodakKugenthiren PermaulAleksandra FucicKui XuAleksandra Karczmarska-WódzkaKuldeep AttriAleksandra KlimczakKuldeep BansalAleksandra KotyniaKuldeep GuptaAleksandra KristoKumar SauravAleksandra LesiakKumar SomyajitAleksandra M. BondžićKumar VaibhavAleksandra Majchrzak-CelińskaKumi Sato-NaraAleksandra MarciniakKumiko SaekiAleksandra PawlakKun LiangAleksandra Piechota-PolanczykKun LuAleksandra RadtkeKun YangAleksandra Swida-BarteczkaKunal MishraAleksandra SzczepkowskaKundan SivashanmuganAleksandra Wisłowska-StanekKun-Eek KilAleksei TikhonovKunihiko NaitoAleksey ChaulinKunihiro TsuchidaAleksey KuznetsovKunio KawamuraAleksey ZaitsevKunio MatsumotoAlena OpattovaKuniyuki KanoAlena OrlenkoKun-Lin TsaiAlena SiskovaKun-Ling TsaiAlenka CopicKuntal DeAles EichmeierKuntebommanahalli ThimmaiahAleš HoloubekKunwei ShenAleš KladnikKuo Hao LeeAleš ObrezaKuo-Cheng LuAleš RučigajKuo-Ching MeiAlessandra AlciatiKuo-Ching YuanAlessandra AmmazzalorsoKuo-Hu ChenAlessandra BiancolilloKuo-Shyan LinAlessandra BoccacciniKurata MoritoAlessandra BorsettiKurt StenmarkAlessandra CapuanoKurt SvobodaAlessandra CuomoKwang Suk LimAlessandra D′AzzoKwang Yeon HwangAlessandra Di MasiKwang-Zin LeeAlessandra FerramoscaKwan-Kyu ParkAlessandra FerraresiKwanuk LeeAlessandra GarauKweon YuAlessandra GhigoKwiMi ChungAlessandra GirottiKwon Kyoo KangAlessandra GovoniKyeong-Man KimAlessandra NapolitanoKyeongryoon LeeAlessandra P. PrincivalleKyeongsoon ParkAlessandra PaganoKyla D. OmilusikAlessandra PelagalliKyle B. WilliamsAlessandra PisciottaKyle BurghardtAlessandra PullieroKyle HaddenAlessandra RampazzoKyle Holmberg ViningAlessandra RenziniKyle M. GardnerAlessandra RossiKyle PoulsenAlessandra StacchiottiKyle SueAlessandra StasiKylie J. WaltersAlessandra ToscoKyohei KinAlessandra ValerioKyohei NakamuraAlessandra ZanolettiKyoji MoritaAlessandro AlaimoKyojiro N. IkedaAlessandro AnselmoKyoko OuraAlessandro AntonelliKyoko Tsukiyama-KoharaAlessandro BertoliKyo-Seon KimAlessandro BittoKyoung Soo KimAlessandro BonardiKyoung-Jin OhAlessandro CampanellaKyoungjoo ChoAlessandro CasiniKyoung-Seok RyuAlessandro CastorinaKyoung-Sik MoonAlessandro D. GenazzaniKyriaki S. PafitiAlessandro De SireKytai Truong NguyenAlessandro De VitaKyu LimAlessandro Di CerboKyu Young ChoiAlessandro FanzaniKyueui LeeAlessandro FaticaKyuho KangAlessandro FiorenzanoKyung Eun KimAlessandro GranitoKyung Jun LeeAlessandro LeutiKyung Pyo KangAlessandro LucchesiKyung Taek RimAlessandro LucidiKyung-Chul ChoiAlessandro MalobertiKyungha LeeAlessandro MangognaKyungjae-andrew YoonAlessandro MarroneKyung-Jin MinAlessandro MatteKyung-Min KimAlessandro MaugeriKyung-Min LimAlessandro MeduriKyung-Mok ParkAlessandro MorettoKyung-Rok YuAlessandro OttaianoKyung-Sun HeoAlessandro ParodiKyuri JoAlessandro PasseraKyu-Sang ParkAlessandro RizzoKyuseok KimAlessandro ScalaKyu-Shik LeeAlessandro ScarsoL. B. RichardsAlessandro ScoppolaL. Kevin LewisAlessandro SinigagliaL. V. KuibidaAlessandro TafuriLacramioara OchiuzAlessandro VichiLăcrămioara OpricăAlessandro VittoriLacramioara PopaAlessandro VolonterioLada AnikinaAlessandro ZampognaLadislav AnděraAlessia CappelliLadislav ŠtěpánekAlessia CasoLaëtitia Trapp-FragnetAlessia FilipponeLaffranchi MattiaAlessia FinottiLafon-Hughes LauraAlessia Maria CossuLajos KovacsAlessia PellerinoLakkakula SatishAlessia PesericoLakshmanakumar KinthadaAlessio D′alessioLakshmi PrabhuAlessio ReggioLalit BatraAlessio RungatscherLalit GolaniAlessio SquassinaLalitha KuradaAlessio TorcinaroLamberto ReAlessio VallettaLamiaa M. A. AliAlex A. SayourLan GongAlex AptLan ZhangAlex ArtyukhinLana McClementsAlex BirgLana NezicAlex GarantoLance LeeAlex J. RathboneLander Egana GorronoAlex JaegerLan-Szu ChouAlex KuchumovLapo TurriniAlexa Karina KlettnerLara S. CarrollAlexa KlettnerLara Valiño-RivasAlexander A. GulinLarbi RhaziAlexander A. IshchenkoLarisa AnghelAlexander A. KorlyukovLarisa LitvinovaAlexander A. ShtilLarisa RyskalinAlexander Aleksandrovich SafonovLarissa BalabanovaAlexander Alexandrovich GromovLarissa KotelevetsAlexander BerezinLarry CroftAlexander BetekhtinLarry OldhamAlexander BunevLars HellmanAlexander CarpinteiroLars KaestnerAlexander DeutschLars MalmströmAlexander DityatevLars MichelAlexander DornLars NorgrenAlexander FoninLars P. KamolzAlexander G. ObukhovLars ScharffAlexander G. TskhovrebovLars TöngesAlexander GorbushinLars-Peter KamolzAlexander I. KokorinLasse LindahlAlexander IgnatovLászló AntalAlexander IvanovLászló BakacsyAlexander J. MichelsLaszlo BodaiAlexander J. SodtLászló CsanádyAlexander LangLaszlo DeresAlexander LarinLászló JicsinszkyAlexander LjubimovLászló Kozma-BognárAlexander M. ShestopalovLászló SomsákAlexander MatyushenkoLászló SzabóAlexander MevesLaszlo TretterAlexander MironovLászló VécseiAlexander MuellerLaszlo VighAlexander N. ShikovLatifa Rbah-VidalAlexander NguyenLaura A. GenovesiAlexander NovikovLaura Alejandra De La RosaAlexander OtahalLaura Ancuţa PopAlexander PanossianLaura BaldomàAlexander PietrasLaura BassolinoAlexander PogrebnjakLaura BergantiniAlexander RappLaura BianchiAlexander RemelsLaura Bravo-ClementeAlexander RevelskyLaura BulgariuAlexander RoeschLaura CaliognaAlexander S. VoronkovLaura CerqueiraAlexander SchrammLaura CrisponiAlexander ShpakovLaura D′AlfonsoAlexander SurinLaura E. SwanAlexander T. H. WuLaura Elena SperlingAlexander TamalunasLaura EspínAlexander TsouknidasLaura Fernández-AlacidAlexander V. BagaevLaura Fusar-PoliAlexander V. LjubimovLaura GalanteAlexander V. MaltsevLaura GambariAlexander V. TimoshenkoLaura GhigliottiAlexander VolkovLaura GiganteAlexander W. EckertLaura Grațiela VicașAlexander WreeLaura Katharina SieversAlexander ZaboronokLaura KerosuoAlexandr Alexandrovich KolobovLaura LagostenaAlexandr DejnekaLaura LindsayAlexandr KazachenkoLaura LocatiAlexandra AntunesLaura M. Hunsicker-WangAlexandra BogomazovaLaura MangiaviniAlexandra Cristina BlagaLaura MarchettiAlexandra Cristina MunteanuLaura MauriAlexandra DubrovinaLaura MaziluAlexandra HarveyLaura Medina-PucheAlexandra M. GouveiaLaura MercataliAlexandra MüllerLaura MicheliAlexandra PauloLaura MitreaAlexandra Ripszky TotanLaura Mora-LópezAlexandra TsidulkoLaura Muinelo-RomayAlexandre BenedettoLaura MusazziAlexandre BerrLaura N. BorodinskyAlexandre CamposLaura N. BurgaAlexandre ChojnowskiLaura NarcisoAlexandre De NonnevilleLaura NigiAlexandre EscargueilLaura OrianAlexandre G. De BrevernLaura PascualAlexandre GoncalvesLaura PasseriniAlexandre GuyLaura PatrussiAlexandre LamasLaura PiquerasAlexandre QuintasLaura RagonaAlexandrina Ferreira MendesLaura RaimondiAlexandrina MunteanLaura RestaniAlexandros GeorgakilasLaura RidolfiAlexandros PapachristodoulouLaura SalvadoriAlexandros ProtonotariosLaura Sánchez-MuñozAlexandros RovasLaura SanzAlexandru BurlacuLaura SciaccaAlexandru Florin RogobeteLaura ScranoAlexandru Mihai GrumezescuLaura Silva-RosalesAlexandru-Florian DeftuLaura SilvestriAlexei B. ChukhlovinLaura VicenteAlexei N. SuminLaura WesseldijkAlexei NabokLaurel HiebertAlexei SlepzofLaureline BerthelotAlexei V. TumanovLauréline RogerAlexey AgranovskyLauren DoumaAlexey AlekseevLauren L. JantzieAlexey ChubarovLaurencas RaslavičiusAlexey ChurovLaurence Gamet-PayrastreAlexey G. GerbstLaurence Hibrand-Saint-OyantAlexey G. KruglovLaurence RisAlexey G. MittenbergLaurens A. Van MeeterenAlexey KovalLaurent ChatreAlexey LuchinskyLaurent ChavatteAlexey LukoyanovLaurent CombettesAlexey MalyginLaurent CronierAlexey NazarovLaurent GatéAlexey PshezhetskyLaurent LimozinAlexey RuzovLaurent MetzingerAlexey S. VasilchenkoLaurent MullerAlexey SarapultsevLauri J. MoilanenAlexey SokolovLauro MeloAlexey V. PindyurinLav SharmaAlexi KissLavanya GoodlaAlexia PolissidisLavinia MareriAlexios PolidorosLawrence H. LashAlexis GalanisLawrence N. DiebelAlexis GautreauLaxmi Narayan MishraAlexis GrandeLazar B. DavidovicAlfonso Blázquez-CastroLazar VujanovicAlfonso Fernández-ÁlvarezLe YingAlfonso Gutiérrez-AdánLea LojkovaAlfonso LagaresLeah M. WuescherAlfonso MartinezLeandro MoreiraAlfonso MuñozLeandro Rodrigues SantiagoAlfonso NavasLeandro SaccoAlfonso Olaya AbrilLeandro SastreAlfonso OyarzabalLeao Martins José ManuelAlfonso T. García-SosaLech B. DobrzańskiAlfonso Varela-LópezLee Bulla, Jr.Alfonso ZambonLee Chaw-NingAlfred GugerellLee Wei LimAlfredo Cabrera OreficeLee-Ann H. AllenAlfredo CaturanoLeena KadamAlfredo CiccodicolaLeena LatonenAlfredo Cruz-RamírezLeena P. BharathAlfredo G. CasanovaLei HuangAlfredo GorioLei WanAlfredo GrilliLei WangAlfredo IacoangeliLeigh PlantAlfredo J. LucendoLeigh-Anne SwayneAlfredo Téllez ValenciaLeila E. RiederAli AbbaraLeila JahangiriAli Abbas RizviLeila YoussefianAli Adem BaharLene Duedahl-OlesenAli Alexander AghdassiLenka BesseAli AtouiLenka KubicovaAli CanbayLenka MaletínskáAli JazirehiLenka MichalcovaAli Mahdavi FardLenka ŠindlerováAli MirzaeiLenka VysloužilováAli MobasheriLennane Michel Espinoza-FonsecaAli R. JazirehiLeo FitzpatrickAli Salehzadeh-YazdiLeo R. QuinlanAlica PizentLeo VeenmanAlice BerardoLeodevico L. IlagAlice BoilèveLeon Garland ColemanAlice BruceLeon StraubAlice ConigliaroLeona GilbertAlice DesmarchaisLeonard B. MaggiAlice Georgia VassiliouLeonard CheungAlice GiontellaLeonard L. DobensAlice H. MacQueenLeonardo BascoAlice IndiniLeonardo BrunoAlice LuddiLeonardo De AssisAlice M. TurnerLeonardo ErminiAlice MelãoLeonardo MastropasquaAlice RothnieLeonardo OñaAlice S. PereiraLeonardo SacconAlice TorresLeonhard MöcklAlicia Ayerdi GotorLeonhard MüllauerAlicia K. ByrdLeontina C. GurguAlicia Manjon SanzLeonzio FortunatoAlicia Rodríguez-BarberoLeopold EckhartAlicia Rodriguez-PlaLeopoldo FornerAlicia Romero-LorcaLeszek SzablewskiAlicia RoqueLeszek SzalewskiAlicja ChrzanowskaLeszek WawiórkaAlicja FormaLeticia MoraAlicja KluczykLetizia De ChiaraAlicja PacholewskaLetizia GiampietroAlicja SułekLetizia MattiiAlida AmadeoLetizia PittoAlida PalmisanoLetizia PorcelliAliesha González-ArenasLevi AndreaAliki KapazoglouLevon KhachigianAlin CiobicaLeyre ZubiriAlin Laurenţiu TatuLhassane IsmailiAlin RaiLi FuAlina BoraLi JiangAlina ConstantinLi LiAlina Gabriela RusuLi LinAlina GeorgescuLi Ren KongAlina KuryłowiczLi Sung HsuAlina PykaLi TanAline P. BeckerLia RimondiniAline Yen Ling WangLiad HindenAlisa BoutinLiam O′NeillAlisa SokolovskayaLi-An ChuAlisha JonesLiana SilvaAlisha PrasadLianbo LiAlison C. WestLi-Ang LeeAlison EastmanLiang ZhaoAlistair J. LeesLiangcan HeAlizée MalnoëLiang-Yin KeAlja Videtič PaskaLianwu FuAlja ZottelLibero VitielloAlla F. FominaLi-Ching ChenAlla MitrofanovaLicia UccelliAlla SalminaLicínio MancoAllan RadaicLicio CollavinAllegra BattistoniLidia CantacorpsAllen O. EghrariLidia FeliuAllen R. ChenLidia GaffkeAllison B. ReissLidia Hanna MarkiewiczAllyson F. O′DonnellLidia Polkowska-KowalczykAlma BalestrazziLidia Szulc-DąbrowskaAlma OsmanovicLidija BarisicAlsu SaifitdinovaLidija TruncaitėAltaf MohammedLi-Han ChenAltaira D. DearbornLi-Han HsuAltaner CestmirLihua ChenAlthea CampuzanoLijian ShaoAlvaro CrevennaLijuan FengÁlvaro GonzálezLilach SoreqÁlvaro Gutiérrez UzquizaLilah GlazerÁlvaro TeijeiraLilia SabantinaAlvin H. SchmaierLilian BassoAlyce RussellLilian I. PlotkinAmadeus DobrescuLiliana BurlibasaAmaia ArranzLiliana KiczakAmaia RodríguezLiliana LayerAmalia Floriou-ServouLiliana Mititelu TartauAmalia PiroLiliya FrolovaAmancio CarneroLiliya VasilevaAmanda C. SharkoLilla TuriákAmanda GendersLiming ChenAmanda GuthLimor LandsmanAmanda IglesiasLin ZhuAmanda Lopez-PicadoLina A. ShehadehAmanda PacholakLina BaranauskieneAmanda UnsworthLina GölzAmanda WasylishenLinards KlavinsAmandine AnastácioLinbo ZhaoAmandine FlouratLinchao LuAmandine GrimmLinda ChaabaneAmandine JullienneLinda L. BenskinAmandine RoviniLinda ParsonsAmann-Winkel KatrinLinda ReisAmany MohamedLinda S. Forero-QuinteroAmar SinghLinda ZuurbierAmaranatha VennapusaLindsay BrownAmaresh C. PandaLindsey Van HauteAmarish YadavLing HeAmber JollyLing LiAmbra Del GrossoLing-Chu ChangAmbrish SinghLingling MiaoAmedeo LonardoLingwen DingAmedeo VetereLing-Wen DingAmeera AlsadeqLingyan ShiAmel DudakovicLinhan LinAmelia A. PetersLinhao RuanAmelia BarilliLinlin MaAmelia CataldiLinlin ZhaoAmelia FullerLinyi ZhuAmelia J. HessheimerLionel CarneiroAmelia LicariLionel CheruzelAmelia M. SilvaLionel MoureyAmelia SalimontiLionel TafforeauAmélie CalmontLionel TarragoAmelie DeglaireLiping WangAmeya KastureLiqing ZangAmie MoyesLirong WangAmin DerouicheLisa CameronAmin MojiriLisa EbiharaAmin ShavandiLisa Juntti-BerggrenAmina RhouatiLisa K. BrentsAminollah KhormaliLisa MapelliAmir Masoud PourrahimiLisa Privette VinnedgeAmir SohrabiLisa Van Den BroeckAmirbabak Sioofy-KhojineLisbet HaglundAmit BeraLisha LiuAmit HalderLisheng KongAmit KumarLishi XieAmit Kumar HalderLitia Alves De CarvalhoAmit PalLi-Ting LeeAmit PandeyLiubov BuchynskaAmitava AdhikaryLiubov N. SkrypnikAmitava MoulickLiubov SkrypnikAmjad KhanLiudmil AntonovAmmad Ahmad FarooqiLiudmila LeppikAmmar TahirLiujiang SongAmnon BustanLivia López-NoriegaAmnon KorenLivio LuongoAmol ShivangeLixian ChenAmos SakweLixin RuiAmosy M′KomaLiza Barki-HarringtonAmr Abd El-WahabLizabeth AllisonAmr ElKelishLluis EspinosaAmram EshelLobelia SamavatiAmrita SarkarLoic LeclercqAmro M. SolimanLoic LemonnierAmy CochraneLoka PenkeAmy D. ShapiroLokesh KumarAmy E. VincentLong Chiau MingAmy FirthLong-Sen ChangAmy HarmsLongteng TangAmy ReeveLonny R. LevinAmy WhitakerLontay BeátaAna Alfonso PiérolaLora Petrie-HansonAna ArnaizLorant JanosiAna Belen Garcia-RedondoLorant KiralyAna Belén HerreroLoredan NiculescuAna BorotaLoredana BergandiAna BugaLoredana BuryAna C. FernandesLoredana F. CiarmielloAna Camara-ArtigasLoredana FrascaAna CampilhoLoredana Liliana HurjuiAna CandiotaLoredana MarcuAna CaruntuLoredana MoroAna CazacuLoredana Sabina Cornelia ManolescuAna Cecília PiresLoredana UrsoAna Cipak GasparovicLorella NavazioAna Clara AprotosoaieLoren W. RunnelsAna Clara MarquesLorena DucaAna Correia-BrancoLorena Ortega MorenoAna CostaLorena TribeAna Cristina GonçalvesLorenz H. LehmannAna CrnkovicLorenzo A. CingolaniAna D. SimonovićLorenzo CarnevaleAna Elena Pérez-CobasLorenzo DragoAna Elena Rodríguez-RodríguezLorenzo FerroniAna Estela LedesmaLorenzo GuazzelliAna EusébioLorenzo LorussoAna FigueirasLorenzo MortaraAna FloresLorenzo PolimenoAna Galán CoboLorenzo RidolaAna Gómez-RamírezLorenzo SempereAna Gonzalez NoyaLorenzo SpadaAna GorostidiLoretta De-ChiaraAna Guerra-LibreroLori A. WalkerAna HorovistizLori RaetzmanAna I. AlvarezLoris PietrelliAna I. Álvarez-MercadoLory Saveria CrocèAna I. ArrobaLothar BreckerAna I. Benítez-MateosLouis GrafAna I. FernandesLouise E. See HoeAna I. FloresLouise MobergAna I. MatesanzLouise MoyleAna IgeaLouise SerpellAna Isabel Álvarez-MercadoLoukas KanetisAna Isabel Ferreira BarbosaLoukianos S. RallidisAna Isabel Fraguas-SánchezLourdes MounienAna J. Chucair-ElliottLu WangAna Julia García-MalinisLuana TonioloAna LosadaLuba PerryAna Luísa De Sousa-CoelhoLuba TchertanovAna LuzioĽubica ĎudákováAna Maldonado-ContrerasLubka RoumeninaAna Margarida PereiraLubka T. RoumeninaAna Maria Da Costa FerreiraLubomir LubomirovAna María Díez-PascualLubomir SkladanyAna Maria Gomez NeoLubos CipakAna María Navarro IncioLubov SnegurAna Maria PutzLubov TimchenkoAna MaroteLuc BidelAna MonteiroLuc BoussetAna MucaloLuc JaegerAna ObesoLuc MaroteauxAna OliveiraLuc NegroniAna P. LunaLuc Philippe Régis BidelAna PenezicLuc TéotAna Pérez De CastroLuc Van KaerAna Raquel SantiagoLuca AmbrosioAna Raquel SoaresLuca AntonioliAna ReisLuca BelliniAna Reis-MachadoLuca BonfantiAna ReulaLuca BrunoAna Rita CarlosLuca BruschiniAna Rita Costa-PintoLuca BurroniAna Rita GomesLuca CravelloAna Salomé PiresLuca De ToniAna SeixasLuca Del GiaccoAna Shek-VugrovečkiLuca Di GianfrancescoAna Sofia Leitão CarvalhoLuca DonazzanAna TeofilovićLuca EspositoAna Teresa Almeida-SantosLuca FalzoneAna TiganescuLuca FerrariAna V. González-De-PeredoLuca FilippiAna VinhaLuca FusaroAna ZubiagaLuca GentilucciAna-Bárbara García-GarcíaLuca GiacomelliAnabel Isabel MarinaLuca Giovanni RegazzoniAnabela Bernardes Da SilvaLuca La NotteAnalisa PastoreLuca Lo NigroAna-Maria BrezoiuLuca LombardoAnamaria BrozovicLuca M. NeriAnamaria Ekert KabalinLuca MadaroAnamarija RoginaLuca MarchettiAnand GururajanLuca MarinelliAnand Prakash SinghLuca Melotti‪Anand SinghLuca MorandiAnanda Ayyappan Jaguva VasudevanLuca PaganoAnanda VasudevanLuca PangrazziAnandharajan RathinasabapathyLuca ProiettiAnanta PaineLuca RaiteriAnanta Prasad ArukhaLuca RevelliAnantha Koteswararao KanugulaLuca Rosario LimiteAnastasia A. PonomaryovaLuca RuiuAnastasia A. ShvetsovaLuca SancinetoAnastasia AsimakopoulosLuca SchioAnastasia De LucaLuca SettanniAnastasia MalekLuca SimulaAnastasia MitseaLuca SteardoAnastasia MpakaliLuca TadiniAnastasia NazarovaLuca TestarelliAnastasia PyriochouLuca TianoAnastasia SpiliopoulouLuca UlianichAnastasia SveshnikovaLuca VaghiAnastasia TsingotjidouLuca VitaleAnastasia VenierakiLucas BrandaoAnastasiia I. PetushkovaLucas ReinekeAnastasiia K. KimeklisLucas Taoro-GonzálezAnastasios E. GermenisLucas Van Den HoogenAnastasios MarkopoulosLucia AltucciAnastasiya SolovievaLucia AnemonaAnastasiya V. PetrovaLucía Barbier-TorresAnastassios PapageorgiouLucia BattistiniAnastasya AnashkinaLucia BossoniAnastazia KeiLucia CaffinoAnasztázia HetényiLucia CarboniAnbazhagan SathiyaseelanLucia CavigliAnca ColitaLucia CsaderovaAnca DinischiotuLucia FrittittaAnca DoceaLucia Garcia GutierrezAnca MazareLucía García-OrtegaAnca Oana DoceaLucia GuidugliAnca PanaitescuLucia MalaguarneraAnca Roseanu ConstantinescuLucía Méndez LópezAnca SimionescuLucia MerolleAnca UngurianuLucía MonacoAnca-Roxana PetroviciLucia MontenegroAncha BaranovaLucia MorbidelliAncuţa Maria JurjLucia NatarelliAndaleb KholmukhamedovLucia PanzellaAnder VergaraLucia PreziosoAnders Christian LarsenLucia RoccoAnders JohanssonLucia ScisciolaAnders MeibomLucia SilvestriniAnders OlofssonLucia StančiakováAnderson GeorgeLucia TerzuoliAnderson Oliveira SouzaLucia UngerAndi Dian PermanaLucia Vieira HoffmannAndjelka KovačevićLucian ConstantinAndrada SerafimLucian CopoloviciAndras BikovLucian CuibusAndrás BikovLucian GorganAndras GaramiLuciana BordinAndrás KünstlerLuciana CacciottolaAndrás PappLuciana GalettoAndras SzaboLuciana MoscaAndraz DovnikLuciana PisaniAndré Ferreira MoreiraLuciana R. TalliniAndré GerberLuciana RennaAndré GillesLuciana TorquatiAndre LechelLuciano De CarlisAndre Luiz Pasqua TavaresLuciano De SioAndre Montiero Da RochaLuciano GalantiniAndre SilvaLuciano PecettiAndre StevenLuciano PirolaAndré TomalkaLucie BednarovaAndrea AlexandreLucie HejnovaAndrea AnesiLucie LanikovaAndrea AngeliLucie LowAndrea AnnoniLucie PlíhalováAndrea ArmaniLuciele MinuzziAndrea ArsiccioLucien N. LameijerAndrea AstolfiLucija Virovic-JukicAndrea BeccioliniLucillia BezuAndrea BenciniLucinda BessaAndrea BerenyiovaLucio CoccoAndrea BernardiniLucio Diaz-FloresAndrea BilgerLúcio FrigoAndrea CaporaleLucrezia BottalicoAndrea CarugnoLucrezia LaterzaAndrea CavazzoniLudger KolbeAndrea CerratoLudmila A. FrankAndrea CitarellaLudmila KazdovaAndrea Clavijo-McCormickLudmila L. SemenychevaAndrea CopettaLudmila VodickovaAndrea DardisLudmilla Morozova-RocheAndrea DefantLudovic JeanAndrea Di CristoforiLudovic TricoireAndrea Di NisioLudovic ZimmerlinAndrea DidierLuigi BennardoAndrea DisanzaLuigi BrunettiAndrea EwaldLuigi CampanellaAndrea FabbriLuigi ChiariniAndrea FusoLuigi De BellisAndrea Ghelli Di RoràLuigi De MasiAndrea GhiroldiLuigi De PetrisAndrea Giuseppe MaugeriLuigi GennariAndrea GiustiLuigi MilellaAndrea Gutierrez MariaLuigi R. CeciAndrea HalseyLuigi SantacroceAndréa HemmerlinLuigi SapioAndrea Herrero-CerveraLuigi VitaglianoAndrea HuwilerLuigia CristinoAndrea IlariLuís AbrunhosaAndrea KissLuis Alberto Pérez RomasantaAndrea KravatsLuís António Paulino PassarinhaAndrea KunovaLuis Antonio Pérez GarcíaAndrea LapucciLuis Carlos Sandoval-HerazoAndrea LuchettiLuis Felipe Santos MendesAndrea LuvisiLuis Fernandez De CastroAndrea MagrìLuis FrancoAndrea ManciniLuís FrijaAndrea Maria GuarinoLuis GonanoAndrea MartiniLuis GoyaAndrea MatteucciLuis M. Alonso-ColmenarAndrea MatucciLuís M. FélixAndrea MessinaLuís M. S. LouraAndrea MohrLuis M. ValorAndrea NuzzoLuis Mario Vaquero-RonceroAndrea Paola Rojas GilLuís Pedro Ferreira RatoAndrea PapaitLuis Perez Garcia-EstañAndrea PelusoLuís PintoAndrea PicciLuís Pinto Da SilvaAndrea PiccinLuís R. RaposoAndrea PoloLuis ReyAndrea PopelováLuís RibeiroAndrea PorcedduLuis RodrigoAndrea RagusaLuis VarelaAndrea RasolaLuisa AgnelloAndrea RoccuzzoLuisa BernardinelliAndrea RomaniLuisa GorzaAndrea ScozzafavaLuisa IommariniAndrea SevcovicovaLuisa LunaAndrea SonninoLuisa PilanAndrea SpallarossaLuisa PolitanoAndrea StoccoroLuisa RicciardiAndrea Thoma-KressLuisa RongaAndrea TicinesiLuisa StrocchioAndrea TrevisanLuisana Di CristoAndrea ViggianoLuiz Gustavo ChuffaAndrea VisentinLuiza HandschuhAndrea WeissLukas CyganekAndrea ZendriniLukas DedAndrea ZilleLukáš KratochvílAndreadi AikateriniLukáš KubalaAndreas BrachnerLukas LacinaAndreas BraunLukas MargaritisAndreas BrunschweigerLukáš NalosAndreas CarusLukas PacalAndreas EbertLukas RichteraAndreas EbneterLukas ValihrachAndreas H. H. KottmannŁukasz BalewskiAndreas HeylandŁukasz BoguszewiczAndreas K. NüsslerŁukasz Jan LaskowskiAndreas KernLukasz KozlowskiAndreas LudwigLukasz KurykAndreas MautnerŁukasz ŁapokAndreas NüsslerŁukasz M. MilanowskiAndreas PatzakŁukasz PaczesnyAndreas PetryŁukasz PajchelAndreas PeyrlLukasz SadowskiAndreas R. JaneckeŁukasz SalwińskiAndreas RitterŁukasz SobiechAndreas RummelŁukasz SobottaAndreas SegerŁukasz StępieńAndreas TrawegerŁukasz SzczukowskiAndreas TzschachŁukasz SzeleszczukAndreas Von Ameln-MayerhoferŁukasz WojtylaAndreas WeberLuke A. YatesAndreas WernerLuke CurtisAndreas-Neil UnterreinerLuke J. MortensenAndreea SoareLuke J. NeyAndreea-iren SerbanLuke R. TembrockAndrei A. DeviatkinLuminita CrisanAndrei KramerovLuminita Gabriela MarutescuAndrei LucaLuminita MarinAndrei PakhomovLuna Maslov BandićAndrei SurguchovLunawati BennettAndreia BarrosoLuping YinAndreia FigueiredoLusheng FanAndreia M. SilvaLu-Sheng HsiehAndreia PeixotoLu-Ting KuoAndreina BrunoLuz Berenice López-HernándezAndrej FrolovLuzalba Sanoja-FloresAndrej KastrinLuzia Maria De-Oliveira-PintoAndrej PerdihLydia E. MatesicAndrej ŠušorLydia VisserAndrej ZupanLynda PartridgeAndreja R. LeskovacLyndon KimAndrés Da Silva CandalLynette HodgesAndrés García-GarcíaLynn Jeanette SavicAndrés López RoldánLynnette CaryAndres MäeLysangela R. AlvesAndres Mendez LucasLyuben ZagorchevAndrés Molero ChamizoLyubov YudinaAndrés TittarelliLyudmila Bel′skayaAndrés TrostchanskyLyudmila RachekAndrew B. RubinM. Anwar IqbalAndrew Edet EkpenyongM. Azizur RahmanAndrew G. StephenM. Carmen MontesinosAndrew G. VolkM. Del Camino De Juan RomeroAndrew J. DingleyM. E. PembleAndrew J. ThompsonM. Elena Díaz-CasadoAndrew L. EamensM. El-Mogy MohamedAndrew L. SnowM. G. HolyavkaAndrew LalooM. M. SatyamitraAndrew MillardM. ManirujjamanAndrew MoorhouseM. Oliver AhlersAndrew N. StephensM. Paola CastelliAndrew NaylorM. Pilar Fernández-MateosAndrew ResnickM. Pilar Vázquez-TatoAndrew Robert BurkeM. QasimAndrew S. DayM. Sheikh MohamedAndrew S. VoreMª Carmen Duran-RuizAndrew W. CraigMa Xenia G. IlaganAndrew W. MaksymiukMaarit TiirikainenAndrew WestwellMaarten J. M. F. ReijndersAndrew WillettsMaarten MeesAndrew WoodMaartje G. HuijbersAndrey A. SavchenkoMaayan WaldmanAndrey A. SinjushinMacarena Sánchez NavarroAndrey A. TyuftinMaciej BuśkoAndrey E. RyabininMaciej ChalubinskiAndrey ElchaninovMaciej GagatAndrey EnyashinMaciej Iek SkrzypczakAndrey KruglovMaciej JaneczekAndrey LetarovMaciej JarzebskiAndrey MarkovMaciej JaśkiewiczAndrey PoddelskyMaciej KapkowskiAndrey R. SuprunMaciej LechAndrey RatushnyyMaciej MaciejczykAndrey SimbirtsevMaciej OstrowskiAndrey TsaturyanMaciej SałagaAndrii DomanskyiMaciej SalagierskiAndrii SlonchakMaciej SkotakAndris DišlersMaciej SkrzypczakAndrius SakalauskasMaciej TarnowskiAndriy ChmyrovMaciej UgorskiAndriy KovalenkoMacko-Podgorni AlicjaAndromachi TzaniMaddalena RipamontiAndrzej BajguzMadelon PaauweAndrzej BakMadelyn GillentineAndrzej BozekMadhu KamleAndrzej BuczyńskiMadhulika TripathiAndrzej EljaszewiczMadhumita ChatterjeeAndrzej KasperskiMadhuvanthi VijayanAndrzej Kazimierz JaworekMadison C. B. PatonAndrzej KoniecznyMadjid HadiouiAndrzej KutnerMaegen BorzokAndrzej PawlikMafalda LaranjoAndrzej PolańczykMafalda OliveiraAndrzej SemczukMafalda PintoAndrzej SlominskiMagaly MartinezAndrzej SzkaradkiewiczMagda FaijesAndrzej TeisseyreMagda MocanuAndrzej TrochimczukMagda PálAndrzej ZielińskiMagdalena BaymakovaAndy M. BaileyMagdalena BogutaAndy Po-Yi TsaiMagdalena Buszewska-ForajtaAndy T. Y. LauMagdalena CalAnelia G. DobrikovaMagdalena Cristina StanciuAnella SavianoMagdalena CzemierskaAneta Agnieszka BartosikMagdalena GrillAneta Cymbaluk-PloskaMagdalena Jabłońska-CzaplaAneta JezierskaMagdalena Jasińska-StroscheinAneta Ostróżka-CieślikMagdalena Jeszka-SkowronAneta PopovaMagdalena K. KowalikAneta Ptak-ChmielewskaMagdalena KaraśAneta Słabuszewska-JóźwiakMagdalena Klimek-ChodackaAneta SłomkaMagdalena Klimek-OchabAneta StachowiczMagdalena KostrzewaAneta StracheckaMagdalena Kowalewicz-KulbatAneta SzymańskaMagdalena KowalewskaAneta WieczorekMagdalena Koziorowska-GilunAnette Gjörloff-WingrenMagdalena Krajewska-WłodarczykAnette KaiserMagdalena Kurnik-ŁuckaAnette Susanne Bøe WolffMagdalena LisÁngel Díaz-OrtizMagdalena MachowskaÁngel Emilio Martínez De AlbaMagdalena Makarska-BialokozÁngel HernándezMagdalena Martínez-CañameroAngel J. MatillaMagdalena Olszanecka-GlinianowiczAngel KirchevMagdalena OrzechowskaAngel L. CorbíMagdalena PeruzynskaAngel L. PeyMagdalena SkarzynskaÁngel Luis García-OtínMagdalena SkotnickaAngel M. MontanaMagdalena SobiesiakAngel MakMagdalena StaszczakAngel Martin-PendasMagdalena StobieckaÁngel Mérida BerlangaMagdalena Sustkova-FiserovaAngel MurcianoMagdalena SzaryńskaAngel NuñezMagdalena SzymanskaAngel PiñeiroMagdalena TorresAngel R. De LeraMagdalena WeingartnerÁngel Romero MartínezMagdalena Wójcik-JagłaÁngel Santiago Sanz OrtizMagdalena WołoszyńskaAngel Vizoso-VázquezMagdalena Wróbel-KwiatkowskaAngela ArmentoMagdalena ZaborowskaAngela BentivegnaMagdalena ŻukAngela CaponnettoMagdalena ŻurawekAngela De BonisMagdolna NagyAngela Di BaldassarreMagne BørsetAngela Gomez-NinoMagnus KjaergaardAngela Gutierrez-CaminoMagnus LiebherrAngela IvaskMagnus MonnéÁngela M. ValverdeMagriet A. Van Der NestÁngela María Gonella-DiazaMahadev RaoAngela Maria Serena LezzaMahboob AlamAngela MastronuzziMahbubul ShihanAngela MeccarielloMahdi ImaniAngela MessinaMahdi Muhammad MoosaAngela MontoneMahin KhatamiAngela PalumboMahmoud AbdellatifAngela PennacchioMahmoud F. SeleimanAngela PolitoMahmoud HuleihelAngela Roberta Lo PieroMahmoud KandeelAngela SandriMahmoud NasrollahzadehAngela Serena MaioneMahmoud SofyAngela SousaMahsa JavidAngelantonio MaglioMahtab SamimiAngélica María Sabogal-GuáquetaMai RosenbergAngelíca OrtizMaicol ManciniAngelika ChroniMaik FinzeAngelika HausserMaike BuschAngelika V. TimofeevaMaiko SuzukiAngelika VoronovaMailo VirtoAngeliki AngelidiMaite InsaustiAngeliki ChroniMaja AbramAngelina AngelovaMaja BoczkowskaAngelisa FrascaMaja Cokarić BrdovčakAngelo Antonio D′ArchivioMaja Jazvinšćak JembrekAngelo De PaolisMaja KozarskiAngelo Di VincenzoMaja Majerić-ElenkovAngelo FacchianoMaja MatulicAngelo LavanoMaja PotokarAngelo MaoMaja SabolAngelo Maria GiuffrèMaja SochalskaAngelo Maria TagliettiMaja TomicicAngelo ZoliMakenzie E. MabryAngelos SikalidisMaki HosokiAngus LindsayMakiha FukudaAnh Tung PhamMakiko Mochizuki-KashioAniello FrancescoMakoto AnrakuAniello MaieseMakoto EndoAnika NagelkerkeMakoto FunakiAnika ToorieMakoto HasegawaAniko Keller-PinterMakoto HashimotoAnil ChekuriMakoto HosoyaAnil K. VermaMakoto KawamukaiAnil SinghMakoto KobayashiAnil TharappelMakoto MakishimaAnilkumar GopalakrishnapillaiMakoto MatsushitaAnimalsLidija ŠverMakoto MiyataAnindya GangulyMakoto MurakamiAnirban ChakrabortyMakoto NaoiAnirban KarMakoto TakafujiAnisa KaurMakoto TsunodaAnisoara CimpeanMaksymilian ProndzynskiAnissa MoktefiMakusu TsutsuiAnita BakraniaMalarz KatarzynaAnita FranczakMalavika AdurAnita Kloss-BrandstätterMalcolm BrodlieAnita KrokoszMalcolm WalkinshawAnita PłazińskaMałgorzata Anna MarćAnita ScipioniMałgorzata Bednarska-MakarukAnita TarbukMałgorzata BiałekAnita ToscaniMałgorzata Brzezińska-RodakAnita UmerskaMalgorzata BurekAnita W. RijneveldMałgorzata CzernickaAnja Elaine SørensenMalgorzata Czystowska-KuzmiczAnja Kathrin JaehneMalgorzata DobrzyńskaAnja Katrin BosserhoffMałgorzata DołowyAnja Krieger-LiszkayMałgorzata Dorota SkibinskaAnja LodeMałgorzata DudaAnja ThalhammerMałgorzata E. ZakrzewskaAnjali KusumbeMałgorzata Ewa DrywieńAnjali SinghMałgorzata GarnczarskaAnjan DebnathMałgorzata GieryńskaAnju KumariMałgorzata GrabarczykAnke HölligMalgorzata GrzesiakAnke SchwarzMalgorzata HolynskaAnke TukkerMałgorzata JarończykAnke WagnerMałgorzata JeleńAnke ZiesenissMałgorzata K. Włodarczyk-BiegunAnket SharmaMalgorzata KajtaAnkica ŠarićMałgorzata KapralAnkit RaiMałgorzata KapustaAnkit RochaniMalgorzata KasztanAnkit SrivastavaMalgorzata KlocAnkita PunethaMałgorzata Kotula-BalakAnkita ShuklaMalgorzata KucinskaAnkita SrivastavaMałgorzata KujawskaAnn E. SutherlandMałgorzata Kus-LiśkiewiczAnn IgoeMalgorzata LekkaAnn LiebertMałgorzata LewandowskaAnn SimpsonMałgorzata LichockaAnn Van SoomMalgorzata MajAnna A. KulminskayaMałgorzata MilczarekAnna A. MarusiakMalgorzata NaleczAnna Adamiok-OstrowskaMałgorzata Natalia MojsakAnna AnnunziataMałgorzata Oczko-WojciechowskaAnna AntsiferovaMałgorzata Pietrowska-BorekAnna ArdévolMałgorzata PiotrowskaAnna ArtatiMałgorzata Polz-DacewiczAnna AtlanteMałgorzata PrzybyłoAnna AviñóMałgorzata SułkowskaAnna B. VolnovaMałgorzata SztankeAnna BarbaszMałgorzata Tatarczak-MichalewskaAnna BellizziMalgorzata Zakłos-SzydaAnna BielenicaMalgorzata ZawadzkaAnna Bilska-WilkoszMałgorzata ZiarnoAnna BirkováMalin WennströmAnna BlendaMalini MukherjeeAnna BrozynaMallesh KurakulaAnna C. HearpsMallikharjun KoriviAnna CariboniMalte LendersAnna Ciemerych-LitwinienkoMalvindar K. Singh-BainsAnna CieślińskaMamede De CarvalhoAnna Cleta CroceMan Kyu ShimAnna CollMan Ryul LeeAnna Concetta BerardiManabu NatsumedaAnna CzubackaManabu NiimiAnna D. SerefkoManabu ShigeokaAnna DamanakiManar IbrahimiAnna De PalmaManasa K. NayakAnna Diez-EscuderoManash K. PaulAnna DittrichManasvi LingamAnna DrabczykManca Tekavčič PompeAnna DuarriMandana Haack-SørensenAnna Duda-MadejMandy PeffersAnna E. KoziołManel CampsAnna FialováMan-ho OhAnna FilipekManijeh PasdarAnna FlorczakManikandan RamasamyAnna Fogdell-HahnManish AdhikariAnna FranzManish BhomiaAnna Gajos-MichniewiczManish JainAnna GenescaManish KumarAnna GiovanettiManish Kumar PatelAnna GładyszMan-Kyo ChungAnna Gora-SochackaManlio VinciguerraAnna GreppiManmeet RavalAnna GvozdjakováManohar KodavatiAnna HejmejManoj B. MenonAnna HurstManoj GargAnna J. KorzekwaManoj KumarAnna Jakubska-BusseMansoore EsmailiAnna Jamroz-WiśniewskaMansoureh BarzegarAnna Jarosz-WilkołazkaManu KumarAnna JaśkiewiczManu PrasadAnna JirkovskáManu RangachariAnna Kablak-ZiembickaManuel A. Fernandez-RojoAnna KałużaManuel AurelianoAnna KawiakManuel Durán-PovedaAnna KędzioraManuel FernandezAnna KicińskaManuel García-JaramilloAnna KippManuel Gomez Del MoralAnna Klimaszewska-WiśniewskaManuel Gómez-GuzmánAnna KlintsovaManuel Hermida PrietoAnna KołtonManuel Jonas RichterAnna KonopkaManuel López-LópezAnna KostarevaManuel M. De SouzaAnna Kostecka-GugalaManuel Marques FerreiraAnna KozłowskaManuel MataAnna KulikManuel Melguizo-GuijarroAnna La TeanaManuel MoralesAnna LaskowskaManuel RamírezAnna LewinskaManuel RequenaAnna LipertManuel ScimecaAnna LopataManuela BanciuAnna Lucia TorneselloManuela ChiperAnna Luise GrabManuela CostanzoAnna M. MastrangeloManuela GariboldiAnna M. OsyczkaManuela GrimaldiAnna MachManuela KimAnna Machoy-MokrzyńskaManuela LabbozzettaAnna MalashichevaManuela LeriAnna ManiaManuela MalatestaAnna MarabottiManuela MoratoAnna Maria AlmericoManuela PiazziAnna Maria BassiManuela RabaglioAnna Maria CuffiniManuela SabatinoAnna Maria GhelliManuela StefanelliAnna Maria PaolettiManuela ViolaAnna Maria SalzanoMao-Lun WengAnna Maria SzekeresMar ÁlvarezAnna Maria WójcikMar Carrión CaballoAnna Martina BattagliaMar InfanteAnna MedyńskaMara A. BonelliAnna MertasMara CucinottaAnna Michalak-StomaMara GyöngyvérAnna MielańczykMara Madalina MihaiAnna Monica Rosaria BiancoMara MarongiuAnna N. BerlinaMara MazzoniAnna NegroniMāra PilmaneAnna NowinskaMara ZilocchiAnna OlejnikMarat MinlebaevAnna P. KryshchyshynMarat R. KhaliluevAnna Palko-LabuzMarc A. IliesAnna PannaccioneMarc C. A. StuartAnna Paradowska-StolarzMarc De La RocheAnna PartykaMarc DorenkampAnna ParusMarc EkkerAnna PawlikMarc GermainAnna PiaseckaMarc GuillonAnna PiccaMarc HendrikxAnna PilutinMarc HenryAnna R. MalikMarc MarescaAnna Rafało-UlińskaMarc PoirotAnna Renata DembskaMarc ReymondAnna RiccioliMarc-André LécuyerAnna Rita BizzarriMarcel AmichotAnna Rzepecka-StojkoMarcel BermudezAnna SanninoMarcel KöhnAnna SansoneMarcel Matei DudaAnna SaponeMarcel PârvuAnna Ścisłowska-CzarneckaMarcel TawkAnna ScomparinMarcel Van De WouwAnna SerefkoMarcel VergesAnna ShchennikovaMarcel ZamockyAnna Silvia PistocchiMarcela LizanoAnna Sroka-BartnickaMarcelina ParrizasAnna StaffasMarcella CantonAnna Stanislawska-SachadynMarcella Pecora MilazzottoAnna StarzyńskaMarcello CandelliAnna StojakowskaMarcello CasaAnna SurdackaMarcello CiaccioAnna SygudaMarcello CostaAnna SynakiewiczMarcello Francesco LinguaAnna TangMarcello MaddaloneAnna Tankiewicz-KwedloMarcello ManfrediAnna TheocharidouMarcello MocciaAnna TintiMarcello PintiAnna TrusekMarcello RomanoAnna TrybalaMarcello TagliaviaAnna V. TsyganovaMarcelo CorreiaAnna Vittoria CarluccioMarcelo D. CatarinoAnna WardowskaMarcelo Tigre MouraAnna WawruszakMarcia De FigueiredoAnna WawrzyńskaMarcia R. LeeAnna WieckowskaMarcin ArciszewskiAnna WilkaniecMarcin BryłaAnna WozniackaMarcin FeldoAnna WysokińskaMarcin HeljakAnna Zimoch-KorzyckaMarcin JozwikAnnabelle Reaux-Le GoazigoMarcin LipowczanAnnadurai AnandhanMarcin MaździarzAnna-Leena SirenMarcin MichalakAnna-Liisa KuboMarcin OpławskiAnnalisa ChiavaroliMarcin OzarowskiAnnalisa GrimaldiMarcin RapaczAnnalisa LonettiMarcin RozalskiAnnalisa MasiMarcin SamiecAnnalisa NatalicchioMarcin SkowronekAnnalisa OrentiMarcin WekwejtAnnalisa PastoreMarcin ZawrotniakAnnalisa SantucciMarcin ZielinskiAnnamaria CiminiMárcio RodriguesAnnamaria LocascioMarco A. Loza-MejíaAnnamaria ManciniMarco Agostino DeriuAnnamaria MassaMarco Alexander Van Den BergAnnamária PallagMarco AnniAnnamaria SandomenicoMarco AtteritanoAnnarita Di MiseMarco BettiAnnarita StringaroMarco BiggiogeraAnn-Christin BrorssonMarco BoAnn-Christin HauMarco BorsariAnne AlbrechtMarco BortolusAnne AndersonMarco BusnelliAnne Angelillo-ScherrerMarco CapogniAnne BernhardtMarco CarusoAnne BlaisMarco CasciaroAnne BordronMarco ChiaramonteAnne ImbertyMarco ChilosiAnne M. AlvarezMarco ConfalonieriAnne Mari RokstadMarco ContardiAnne Skaja RobinsonMarco CorazzariAnne StrohbachMarco CordaniAnne W. BeavenMarco DettoriAnne-Catherine HeuskinMarco Di AntonioAnne-Claire JacominMarco FerrariAnneke A. VeenstraMarco FiorentinoAnne-Laure Chateigner-BoutinMarco FraaijeAnnelaure DamontMarco FracassettiAnne-Laure FavierMarco G. AlvesAnnelie-Martina WeinbergMarco GebiolaAnnemarie KäsbohrerMarco GentileAnnemarie WeißenbacherMarco GerdolAnne-Sophie Vercoutter-EdouartMarco GiampàAnne-Sophie WoznyMarco GiorgioAnnett Dorner-ReiselMarco GrossiAnnette AignerMarco HerlingAnnette LisMarco InfanteAnnette NassuthMarco LolicatoAnnette SchürmannMarco MainardiAnnibale PucaMarco ManfrediniAnnick DuboisMarco MangiagalliAnnie JoubertMarco MarencoAnnie MayenceMarco MascittiAnnie RobicMarco Matucci CerinicAnni-Maija LindenMarco MazzoranaAnn-Kristin Östlund-FarrantsMarco MontagnerAnnunziata MauroMarco MorselliAnoop AlexMarco PessoaAnoop ArunagiriMarco PevianiAnoop KumarMarco PiccoliAnoop RawatMarco PonzettiAnselm HornMarco PortelliAnselm MakMarco PrunottoAnsgar PoetschMarco QuagliaAnshu AgrawalMarco RaffaeleAnshu AlokMarco RinaudoAnssi PeuronenMarco RohdeAnthony GuihurMarco RosinaAnthony HaighMarco SalerAnthony J. BaucumMarco SalernoAnthony KhongMarco ScarselliAnthony KusalikMarco SchiavoneAnthony MathironMarco SegattoAnthony MillarMarco SgarbantiAnthony MukwayaMarco SisignanoAnthony QuéroMarco TafaniAntía González PereiraMarco VincetiAntimo CutoneMarco WachtelAntimo Di MaroMarcos Arturo Martínez-BanaclochaAntje BanningMarcos Edel Martinez-MonteroAntoaneta TonchevaMarcos Egea Gutiérrez-CortinesAntoine DrieuMarcos López-HoyosAntoine H. ChaanineMarcos MoraesAntoinette Van Der KuylMarcus GrimmAntón Barreiro IglesiasMarcus H. HolschbachAnton G. KutikhinMarcus HorstmannAnton GisteråMarcus O. W. GrimmAnton H. Van Den MeirackerMardelle AtkinsAnton HartmannMarei SammarAnton JettenMareike Schallenberg-RüdingerAnton KiselevMarek HartlebAnton KutikhinMarek IsbrandtAnton ManakhovMarek K. DobkeAnton O. SvininMarek KieliszekAnton P. TyurinMarek KochańczykAnton PetrovMarek KonopAnton PopovMarek KoutnýAnton S. BychkovMarek KrzystanekAnton S. TsybkoMarek MajdanAnton ZavialovMarek MuriasAntonella ArgentieroMarek OrłowskiAntonella CalogeroMarek PawlikowskiAntonella CaniniMarek PruszynskiAntonella CurulliMarek SanakAntonella Di SottoMarek SkonecznyAntonella FerranteMarek SkrzypskiAntonella FrassanitoMarek SzostakAntonella GoriMarek VrbackýAntonella LiantonioMarek WisniewskiAntonella PaladinoMaren Ortiz-ZarragoitiaAntonella PantaleoMaren PodewitzAntonella PattiMargaret F. BennewitzAntonella Ragnini-WilsonMargaret L. BritzAntonella RosiMargaret OttavianoAntonella SgarbossaMargaret ParkAntonella SmeriglioMargareta P. CorreiaAntonella TomassettiMargarida EspadaAntonella VirgilioMargarita A. SazonovaAntonello Di PaoloMargarita IvanovaAntonello NovelliMargarita Jimenez-PalomaresAntoni BanaśMargarita Rodríguez-KesslerAntoni WiedlochaMargarita VigodnerAntonia Ávila-FloresMargaritis AvgerisAntonia CianciulliMargherita CarlettiAntònia Costa-BauzáMargherita EufemiAntonia PatrunoMargherita G. De BiasiAntonietta BernardoMargherita MaioliAntonietta LiottiMargherita MaranesiAntonietta MessinaMargherita TumedeiAntonietta StellavatoMargit BalázsAntonija Jurak BegonjaMargit L. H. RiisAntonina GiammancoMargot ZöllerAntonina SidotiMari DezawaAntonino GermanaMaria A. BaturovaAntonino ManiaciMaria A. BonifacioAntonino NatalelloMaria A. BrehmAntonino TuttolomondoMaria A. LongoAntonio AndresMaria A. VulfAntonio AntónMaria Addolorata BonifacioAntonio ApicellaMaria Amparo AssisAntonio Arnaiz-VillenaMaria Angela LosiAntonio ArtiguesMaría Ángela OlivaAntonio BaldiniMaría Ángeles Martínez-CuestaAntonio BauzáMaria Angeles RojoAntonio BellastellaMaria Antonietta De LucaAntonio BernadMaria Antonietta PanaroAntonio BiondiMaria AragonaAntonio BlancoMaría Asunción Campanero-RhodesAntonio CarettaMaria BabakAntonio CelliniMaria BagattiniAntonio ChecaMaria BartosovaAntonio Di MartinoMaria BatoolAntonio Di StefanoMaria Beatrice MorelliAntonio FacchianoMaría Begoña Cachón-GonzalezAntonio FerreiraMaria BitontiAntonio FortunatoMaria BloksgaardAntonio FrigeriMaria Borrell-PagesAntonio FronteraMaria BryszewskaAntonio G. SolimandoMaría C. ArufeAntonio GarciaMaría C. BolarínAntonio GiordanoMaria C. TanzerAntonio GiovinoMaría Camacho EncinaAntonio GomezMaría Carmen Blanco-LópezAntonio Gonzalez-BulnesMaria Carmen Iglesias-OsmaAntonio Henrique MartinsMaria Celeste DiasAntonio J. HerreraMaria Clara Vieira Dos SantosAntonio J. Leon-GonzálezMaria Concetta GelosoAntonio J. MotaMaria CondelloAntonio Jesus FernandezMaria ContaldoAntónio Jorge GuiomarMaria CorosAntonio José Ruiz-AlcarazMaria CosenzaAntonio L. SerranoMaria Cristina AngeliciAntonio LaghezzaMaria Cristina BudaniAntonio LucacchiniMaria Cristina De MartinoAntonio M. Pico AlfonsoMaria Cristina De RosaAntonio MaccioMaria Cristina MenzianiAntonio ManentiMaria Cristina PetraliaAntonio Mario ScanuMaria Cristina SiniAntonio MasMaría D. CouceAntonio MastinoMaria D. Giron-GonzalezAntonio Mendes-SousaMaria DalamagaAntonio MichelucciMaria De FalcoAntonio MirijelloMaria De GrandisAntonio Molina-CarballoMaria De La Cruz Cardenosa RubioAntonio NarzisiMaria De Los Reyes Gámez-BelmonteAntonio NennaMaría De Lourdes Moreno AmadorAntónio NogueiraMaría Del Amor HurléAntonio OlivaMaría Del Carmen Ortuño-CostelaAntonio OrlacchioMaria Do Carmo PeluzioAntonio PalazzoMaria Dolores LedesmaAntonio PalladinoMaria DomercqAntonio Palumbo PiccionelloMaria E. M. AraujoAntónio PauloMaria Elena MelicaAntonio PezoneMaria Elisa MancusoAntonio ReyMaria Elisabeth StreetAntónio RoldãoMaria Elisabetta ClementiAntonio RomerosaMaría EstebanAntonio RosatoMaría Eugenia GonzálezAntonio Ruiz CanalesMaria ExindariAntonio Sanchez-RuizMaría F. MontenegroAntonio SchiattarellaMaria Felice BrizziAntónio Sebastião RodriguesMaria FilekAntonio Simone LaganàMaria Filomena BotelhoAntonio TiberiniMaria Filomena CaeiroAntonio Victor Campos CoelhoMaria Francesca BaiettiAntonio ZuorroMaria Francesca CardoneAntonio-Javier ChamorroMaria Francesca MilazzoAntonios A. AugustinosMaria G. DetsikaAntonius PlaggeMaria G. KhrenovaAntony CooperMaria GalanAnttonio GiordanoMaria GazouliAnu ChackoMaria Giovanna FrancipaneAnuj AhujaMaria Giovanna LupoAnuj KumarMaria Giovanna ScioliAnúp PandíțhMaria Giuseppina MianoAnupam AichMaría Gómez-SerranoAnupam DhasmanaMaria Grazia BottoneAnuraag BoddupalliMaria Grazia GiansantiAnurag ParanjapeMaria Grazia ManeraAnurag SharmaMaria Grazia MolaAnush KosakyanMaria Grazia TozziAnusha AdityaMaria GreabuAnuska V. AndjelkovicMaría GuiradoAnwar RayanMaria H. UlvmarAnwesa KarmakarMaria Helena CasimiroAparajita BanerjeeMaria Helena FernandesAphrodite TsaballaMaría Hernández-SánchezApirat ChaikuadMaría Hortigón-VinagreApolonia SieprawskaMaria IkonomopoulouApostolos BeloukasMaría Iniesta CuerdaApostolos BossiosMaría Iniesta-CuerdaApostolos KalivasMaria Irene BelliniApostolos ZaravinosMaria Isabel Hernández-AlvarezApril DarlingMaría Isabel Torres LópezApril HastwellMaria IskraApurba Krishna BhattacharjeeMaría J. CalzadaAraceli CastilloMaría J. HortigüelaAram DavtyanMaria J. MatosArancha Llama-PalaciosMaría Jesús HerreroArantza InfanteMaría Jesús Lobo-CastañónAranzazu ManzanoMaría Jesús Núñez-IglesiasAránzazu MedieroMaria João FerreiraArash NavranMaria João MenesesAravind KshatriMaria João RamalhoArchna RaviMaria João RamalhosaArghya RayMaria João RodriguesAri KoivistoMaria Joao SilvaAria BaniahmadMaria João SousaAriadna BargielaMaría José AlcarazAriana HuditaMaria José AmengualArianeb MehrabiMaría José García-PolaArianna CalistriMaria José Gómez-TorresArianna Carolina RosaMaría Julia AlthabegoitiArianna GelainMaria K. DahleArianna GiacominiMaria K. SvenssonArianna ScuteriMaria KalivaArianna VigniniMaria KoukoulakiArieh MoussaieffMária KovalskáAriel KaminskiMaria KurańskaAriel RamírezMaria L. LozanoAriela Gordon-ShaagMaria L. MaceArijit BiswasMaria L. MateusArijit SenguptaMaria L. PerepechaevaArijita SarkarMaria L. SemenovaArimantas LionikasMaria Laura AllendeArindam BhattacharjeeMaria Laura ErminiArindam ChakrabartyMaria Laura GiuffridaAris GiannakasMaria Laura GomezAris TsalouchosMaria Laura IddaAristea K. BatsaliMaria LebedevaArjen E. Van ′t HofMaria Leilani TorresArkadij SobolevMaria Leite-De-MoraesArkadiusz ArtyszakMaría Linares GómezArkadiusz ChworosMaria Llanos CasanovaArkadiusz JózefczakMaría Llorens-MartínArkadiusz KosmalaMaria LucaArkadiusz MatwijczukMaria Luisa CalabròArkadiusz OrzechowskiMaria Luisa Louro MartinsArkadiusz UrbańskiMaria Luisa Savo SardaroArlyng GonzalezMaria Luisa TorreArman RahmanMaría Luz CouceArmanda E. SantosMaría M. Morales Suárez-VarelaArmando A. Rodríguez AlfonsoMaria M. ZanoneArmando Alexei RodríguezMaria ManconiArmando ZarrelliMaria Manuela GasparArmandodoriano BiancoMaria MarcheseArmin KrausMaria MargalefArmin ZebischMaría Martínez-NegroArmond DaciMaria MazzoniArnab ChinaMaria Miklasińska-MajdanikArnab GhoshMaria MirabelliArnaud BianchiMaria MohoraArnaud FironMaria MonsalveArnaud LombardMaría Moreno-del ÁlamoArnaud MachelartMaría MorosArnd BaumannMária MörtlArne RaasakkaMaria MoschoviArnon KarniMaria MrakovcicArokiyaraj SelvarajMaría Muñoz CaffarelAron B. FisherMaria NamwanjeArpád DobolyiMaria Navarro-MendozaÁrpád DobolyiMaria NikodemovaÁrpád Ferenc KovácsMaria NikolovaArpad KarsaiMaría Orosia Lucha-LópezArpan AcharyaMaria P. A. BaldassarreArrigo CiceroMaria P. GiovannoniArsen S. MikaelyanMaría P. PortilloArshi KhanamMaria P. RubtsovaArtem BlagodatskiMaria PapadakiArtem S. KasianovMaria PaparoupaArtem Yu ManyakhinMaria PassanteArtemissia-Phoebe NifliMaria Patrizia StoppelliArthur GriderMaria PerdomoArthur LustigMaría Pérez-FernándezArti V. ShindeMaria PerticoneArtini Paolo GiovanniMaria PetrosinoArto PulliainenMaria Pia AdorniArtur BanachMaria Pia FelliArtur GurgulMaria Pia GalloArtur J. M. ValenteMaria PilarskaArtur KasprzakMaria Pino-YanesArtur MezheyeuskiMaria PratArtur PałaszMaria RapaArtur RibeiroMaria Rapala-KozikArtur TurekMaria Rita FabbriziArturo BecerraMaria Rosaria De MiglioArturo BevilacquaMaria Rosaria RicciardiArturo CesaroMaria Rosaria VarìArturo Ibáñez-FonsecaMaría Rosario Isabel LuquínArturo López CastelMaria RuzzeneArturo Susarrey-ArceMaria Salomé GomesArulkumar NagappanMaría Sanz-De La GarzaArun Kumar KondadiMaría SerranoArun Kumar TharkeshwarMaria SkibińskaArun Prakash PeriyasamyMária ŠkrabišováArun RajamohanMaria SkrzyszowskaArun UpadhyayMaria SpaneddaAruna SharmaMaria SpletterArunas StirkeMaria Stefania SpagnuoloArvi RaukMária SzekeresÅsa Fex SvenningsenMaria Teresa CambriaÅsa K. WallinMaria Teresa ColominaÅsa PetersénMaria Teresa GentileAsadur RahmanMaría Teresa Gómez-MuñozAsaf AchironMaria Teresa MontagnaAsaf WilenskyMaria Teresa RocchettiAsako YamayoshiMaria TikhonovaAsha KumariMaria ValentiAshana PuriMaria Valeria CataniAsher BashiriMaria Valeria GiuliAsher OrnoyMaria Vazquez-HernandezAshfaque MemonMaria VenihakiAshim GuptaMaría Victoria Martínez-HuertaAshim PaulMária VilkováAshiqur X. RahmanMaria Vittoria CubellisAshish JainMaria Wolun-CholewaAshish RanjanMaria WrobelAshish RawsonMaria Zelinda RomanoAshit RaoMariachiara BuccarelliAshkan BighamMariachiara TrapaniAshlesh K. MurthyMariaelvina SalaAshley J. SniderMaría-Esperanza CerdánAshok AspatwarMariafrancesca ScaliseAshok BidwaiMaria-Giuliana VannucchiAshok KumarMaria-Ioanna ChristodoulouAshok MandalaMarialuigia RaimondoAshot SargsyanMarialuisa AppetecchiaAshraf El-KereamyMariam GaidAshraf Yusuf RangrezMarian BresticAshu JohriMarian EvingerAshutosh TripathiMarian SzebenyiAshwani SharmaMarian ValkoAsia Fernandez CarvajalMariana Eugenia MuresanAsif RazaMariana FerreiraAsif ShajahanMariana FioriAsim EjazMariana FloriaAsker KhapchaevMariana Petronela (Petra) HangaAsli OzmenMariana RochaAslihan TemelMariana SantosAsrar AhmadMariana Saucedo-GarcíaAssia EljaafariMariana Varna-PannerecAssunta BertacciniMariangela AgamennoneAssunta PozzuoliMariangela CentroneAssunta SellittoMariangela De RobertisAsta JuzenieneMariangela GarofaloAsuncion Garcia-SanchezMariangela ManciniAsuncion RocherMariangela MarrelliAtanu ChakrabortyMariangela ScaliseAtashi SharmaMarianna Flora TomaselloAthanasia PapazafiropoulouMarianna HagidimitriouAthanasia PrintzaMarianna SzczypkaAthanasios BikasMarianna TriantouAthanasios ChalkiasMarianna TzanoudakiAthanasios KoukounarasMarianne BrodmannAthanasios MavropoulosMarianne KollerAthanasios PapakyriakouMarianne SpalingerAthanasios SalifoglouMariano Martinez-VazquezAthanasios TragiannidisMariano OstuniAthar AnsariMariano Ponz-SarviseAthar AzizMariano VenanziAthina AngelopoulouMariarita BrancaccioAtiđa SelmaniMariarita GalbiatiAtsuhito A. NakaoMariarosaria ConteAtsuki NaraMaribel R. PortillaAtsushi FukushimaMarica B. EricsonAtsushi IwaiMarica BakovicAtsushi KoikeMarica CarielloAtsushi MakimotoMarie BaucherAtsushi P. KimuraMarie BillaudAtsushi SakaiMarie BrandtAtsushi SakamotoMarie CastetsAtsushi TakaiMarie Christine Morel-KoppAtsushi TanakaMarie DavídkováAtsushi TomokiyoMarie DufresneAtsushi YamashitaMarie FrimatAtte Von WrightMarie Hélène David CordonnierAttila AmbrusMarie Lue AntonyAttila AszodiMarie SisslerAttila BecskeiMarie UmberAttila BendeMarie-agnes Dragon-DureyAttila BenyeiMarie-Hélène Avelange-MacherelAttila G. VéghMarie-Helene DelvilleAttila Gábor SzöllősiMarie-José GoumansAttila HegedűsMariella DonoAttila L. ÁdámMarielle AfanassieffAttila ÖrdögMarielle SaclierAttila ReményiMarie-Louise HammarskjöldAtul B. MehtaMarie-Odile BaudementAtul Singh RathoreMarie-Paule Felder-SchmittbuhlAubrey WeigelMarie-pierre JunierAudrey RousseauMarie-Pierre MoisanAugust WrotekMarietta HerrmannAuguste FernandesMarietta Margit BudaiAugustin M. OfiteruMariëtte E. G. KranendonkAugusto OrlandiMariia AndrianovaAugusto StancampianoMariia NesterkinaAun RazaMarija HefferAura RusuMarija JozanovićAurel DiaconMarija MilanovićAurel MaximMarija-Magdalena PetrinovicAurel Popa-WagnerMarijana CurcicAurelia Magdalena PisoschiMarije BartelsAurelia Walczak-DrzewieckaMarijn SpeeckaertAurélie Gousset-DupontMarika CordaroAurelie RakotondrafaraMarika MassaroAurélie Tchoghandjian-AuphanMarika MokouAurélien DeniaudMarika PellegriniAurelio CiancioMarika QuadriAurelio LoricoMarike W. Van GisbergenAurora DanieleMariko SaitoAurora DiotalleviMariko SeishimaAurora MaurizioMariko Taniguchi-IkedaAurora StorlazziMarilena RonzanAusra MarcinkevicieneMarin MicutzAussie SuzukiMarin ŞenilăAvijit PramanikMarina ArrietaAvinash KadamMarina CaldaraAvinash WaghrayMarina CampioneAvishek MitraMarina CvetkovskaAviv ShaishMarina ElliAvri Ben-Ze′evMarina FericAvshalom LeibowitzMarina Garcia-MaciaAwdhesh MishraMarina I. GiannottiAxel HartkeMarina IoveneAxel KünstnerMarina KalyuzhnayaAxel LorentzMarina KarpenkoAxel MogkMarina KhodanovichAxel SteigerMarina Kleopatra BozikiAxel T. NeffeMarina KolomytsevaAya AkimotoMarina LauraAyae Sugawara-NarutakiMarina LesinaAyako MiuraMarina MariniAyako OhyamaMarina Montagnani MarelliAyako YamaguchiMarina MuzzaAyami MatsushimaMarina P. Arrieta DillonAyan SadhukhanMarina P. IsaevaAyataka FujimotoMarina PadkinaAydin GülsesMarina PaolucciAyelign AdalMarina PiscopoAyhan KocerMarina QuartuAylin ReidMarina SagnouAymara MasMarina SanticAyoung JeongMarina SpînuAyrat U. ZiganshinMarina Svetec MiklenićAysegül AksanMarina V. DunaevaAyumi SugiuraMarina Villanueva-PazAyush RanawadeMarine CouéAyyalusamy RamamoorthyMarine PotezAyyanar SivananthamMarinella TedescoAzzurra StefanucciMarino VilovićB. Bachiller-BaezaMario AbateB. Daan WestenbrinkMario Alberto Burgos-AcevesB. ZgardzińskaMario BortolozziBabak EslamiMario C. De TullioBabar MurtazaMario CioceBabasaheb MatsagarMario CostaBabhrubahan RoyMario D. ToroBa-Bie TengMario D′AcuntoBabken AsatryanMario De FeliceBahar PatlarMario DiazBahareh AzimiMario FotiBahram SamanfarMario G. HollomonBaiba JansoneMario InclánBaiba SvalbeMario LatendresseBaicus AndaMario LovrićBailin LiuMario MardirossianBailin ZhaoMario MikulaBal Krishna ChaubeMario Milco D′EliosBalaji KrishnamacharyMario OlleroBalaji NagarajanMario PiccioliBalamurali Krishna VasamsettiMario PicozzaBalapal S. BasavarajappaMario RangoBalasubrahmanyam AddepalliMario RaspantiBalázs BarnaMario RothbauerBalazs MargitMario TiribelliBalázs MayerMario U. MondelliBalazs MurnyakMario ValentinoBalazs SarkadiMario VerdicchioBalázs SarkadiMariola DutkiewiczBalik DzhambazovMariola HerbetBalint L. BalintMariola Świderek-MatysiakBalkrishna GhimireMarion BouchecareilhBanhi BiswasMarion CremerBao HongliangMarion CurtisBao-Jian DingMarion EhrichBaotong ZhangMarion MussbacherBaoye HeMarios G. KrokidisBaptiste BidonMarios KrokidisBaptiste PanthuMāris KlavinsBaran A. ErsoyMarisa BriniBarbara Ann AmbroseMarisa E. E. JaconiBarbara Blanco-FernandezMarisa MadridBarbara Borawska-JarmułowiczMarisa PassosBarbara BrognaMarisa ShiinaBarbara CelliniMarisela VélezBarbara CisternaMarisol López-LópezBarbara CorteseMaristella MastoreBarbara DarišMarit Nilsen-HamiltonBarbara De AngelisMarius BillBárbara FerreiraMarius DryschBarbara FrąszczakMarius MiricioiuBarbara GhinassiMarius ReglierBarbara GierlikowskaMarius UeffingBarbara Hawrylak-NowakMariusz Aleksander BromkeBarbara HenriquesMariusz DąbrowskiBarbara HissaMariusz FleszarBarbara J. FuegerMariusz HartmanBárbara J. HenriquesMariusz JasińskiBarbara JachimskaMariusz KlenckiBarbara JarząbMariusz KodaBarbara KorbeiMariusz KusztalBarbara ŁapińskaMariusz MitorajBarbara LiederMariusz MojzychBarbara LipińskaMariusz SacharczukBarbara ŁotockaMariusz StasiolekBarbara MaciejewskaMariusz TichoniukBarbara MarengoMariusz Tomasz DziadasBarbara MavroidiMariyana AtanasovaBarbara MiziakMarjaana RantalaBarbara MoeppsMarjan RupnikBarbara MognettiMark A. KleinBarbara MontiMark BerryBarbara MulloyMark BuzzaBarbara N. BorsosMark C. HarrisonBarbara NawrotMark C. HowellBarbara Pacholczyk-SienickaMark D. BakerBarbara PanunziMark D. DaniellBarbara PipanMark D. WilkinsonBarbara Przybylska-GornowiczMark DallasBarbara PucelikMark G. HamiltonBarbara RóżalskaMark G. HoogendijkBarbara RuaroMark G. LefsrudBarbara RuggieroMark GorrellBarbara Ruszkowska-CiastekMark J. DanielsBarbara S. StonestreetMark Klitgaard NøhrBarbara Sosnowska-PasiarskaMark L. GrimesBarbara SymanowiczMark LowryBarbara Szeiffova BacovaMark NicklausBarbara ToffoliMark NixonBarbara TokarzMark PraetoriusBarbara TorselloMark S. ScherBarbara WasilewskaMark SchultzBarbara WiszniewskaMark ZiemannBarbara ZdzisińskaMarkéta PernisováBarbora AugstenováMarketa SamalovaBarbora BrodskaMarko GerićBarbora DvorankovaMarko KnollBarbora VeseláMarko KumrićBarlin O. OlivaresMarko MarhlBarry D. KyleMarko SamardžijaBarry J. ParsonsMarkus BlaessBarry J. SessleMarkus DuechlerBarry MilavetzMarkus FendtBarry S. FlinnMarkus HaugBart CuypersMarkus HoffmannBart De GeestMarkus KippBart NieuwenhuisMarkus KrebsBart VandekerckhoveMarkus MandlBartłomiej GrygorcewiczMarkus NieberlerBartlomiej JankiewiczMarkus OppelBartlomiej KalaskaMarkus PirklbauerBartłomiej KostMarkus S. JördensBartlomiej KrazinskiMarkus StorvikBartłomiej PerekMarkus WolfienBartłomiej PotaniecMarla BerryBartlomiej TroczkaMarlena Gąsior-GłogowskaBartolome SabaterMarlena GodlewskaBartolomé VilanovaMarlena SzalataBartosz ForoncewiczMarlena ZyśkBartosz MałkiewiczMarlene CervantesBartosz PulaMarley J. BinderBartosz SekulaMarlies BallegeerBartosz TrzaskowskiMarloes DekkerBasant Kumar ThakurMarmagne AnneBashar AmerMarna E. EricsonBashayer R. Al-MubarakMarouane BaslamBashir LawalMarsha PierceBasil GrüterMarta A. Fik-JaskółkaBasilia ZingarelliMarta Aandres-MachBasini GiuseppinaMarta Anglada-HuguetBastian SchirmerMarta BaliettiBaubak BajoghliMarta BautistaBeata Bujak-GizyckaMarta CarvalhoBeata Butruk-RaszejaMárta CzakóBeata BystrowskaMarta D′AmoraBeata Da̧browska-BoutaMarta De ZottiBeata GabryśMarta F. EstradaBeata Greb-MarkiewiczMarta Fernández GalileaBeáta HolečkováMarta GarcíaBeata Kalska-SzostkoMarta GomarascaBeata KorchowiecMarta GrecoBeata KurcMarta GrodzikBeata M. Gruber–BzuraMarta HalasaBeata MalachowskaMarta Hałas-WiśniewskaBeata MyśkówMarta HenklewskaBeata OlejnickaMarta Hernández-GarcíaBeata SadowskaMarta J. FiołkaBeata SiekluckaMarta KarazniewiczBeata StaniszMarta KepinskaBeata SzczepanikMarta KiezunBeate M. KümmererMarta KwapiszBeatrice AraminiMarta L. DeDiegoBeatrice Cobucci-PonzanoMarta LaranjoBeatrice MacchiMarta MarmiroliBeatrice Mihaela RaduMarta MartínezBeatrice OehlerMarta MazurBeatrix DienesMárta MedveczBeatriz Buentello-VolanteMarta MellaiBeatriz I. Fernández-GilMarta MurgiaBeatriz LagunasMarta NapieralaBeatriz LimaMarta Nunes Da SilvaBeatriz MerinoMarta OlahBeatriz Sabater MuñozMarta OlejniczakBeatriz San-MiguelMarta OliveiraBeatriz SastreMarta Otero-VinasBeatriz Sevilla-MoránMarta PajaresBeatriz Vazquez BeldaMarta Pérez GonzálezBeatrycze NowickaMarta PrietoBedabrata SahaMarta R. RomeroBednarczyk DominikaMarta RachelBee K. TanMarta RoldoBeenu Moza JalaliMarta RusekBegoña CánovasMarta RybskaBegoña HerasMarta S. CarvalhoBegüm OkutanMarta SacchettiBehnam Asgari LajayerMarta SarkozyBehnam RashidiehMarta StojakBehnam VafadariMarta SzandrukBehzad Behzad FarahaniMarta SzekalskaBehzad M. ToosiMárta SzéllBekim SadikovicMarta ToczekBéla KissMarta TomaszkiewiczBela KocsisMarta TomczykBela OzsvariMarta VallinoBela PappMarta VerneroBelén Callejón LeblicMarta WojewodaBelinda Ann Di BartoloMarta WoźniakBen MontpetitMarta Wysocka-MincewiczBen WielockxMarta ZagrebelskyBen WilliamsMarta ZaràBendahmane MounirMarta Ziegler-BorowskaBendik C. BrinchmannMartha Lopez-CanulBenedetta DonatiMartha Patricia Gallegos-ArreolaBenedetta E. FornasariMartha SkerrettBenedetta FerraraMartí GimferrerBénédicte Brounais-Le-RoyerMartijn HoesBénédicte DurandMartijn J. J. FinkenBenedikt LinderMartin A. SchroerBenesh JosephMartin BakhtBeniamin Oskar GrabarekMartin BartasBenita WiatrakMartin BaumgartenBenito A. YardMartin ČernýBenjamí Oller-SalviaMartin ChanBenjamin BrigantMartin Christian MichelBenjamin D. BrooksMartin D. BergerBenjamin DelpratMartin DračínskýBenjamín Fernández-GutiérrezMartin FischerBenjamín FloránMartin GerickeBenjamin FreyMartin GustavssonBenjamin GantenbeinMartin HerzbergBenjamin J. WylieMartin HoheneggerBenjamin JensenMartin HundBenjamin KochMartin J. HoogduijnBenjamin L. KingMartin JaburekBenjamin LandMartin JungBenjamin NeumanMartin K. ThomsenBenjamin OrsburnMartin KabelácBenjamin PommerrenigMartin KelloBenjamin VerillaudMartin KotoraBenjamin VoellgerMartin OlssonBenno Ter KuileMartin P. PlayfordBenoit ChenaisMartin PallBenoit D′AutreauxMartin PaparellaBenoit GauthierMartin PecBenoit MiottoMartín Pérez-SantosBenoit PaquetteMartin PešlBenoît PourcetMartin PižlBenson Chellakkan SelvanesanMartin ReinerBeom Jin KimMartin RootBerit HippeMartin S. StaegeBerna C. ÖzdemirMartin SchmelzBernadeta SzewczykMartin SchmidtBernadett PappMartin StimpfelBernadette M. M. ZwaansMartin StoddartBernard HaendlerMartin StoneBernard J. JasminMartin SvobodaBernard KlonjkowskiMartin SztachoBernard Laurent SchneiderMartin ValisBernard LeungMartin Villalba GonzalezBernard PossidenteMartin VojtekBernard RoelenMartin W. BerchtoldBernardino ClavoMartin WittBernardo OrdasMartin ZenkeBernat CrosasMartina AlvarezBernat SoriaMartina BancirovaBernd HeinrichMartina C. Herwig-CarlBernd J. Schmitz-DrägerMartina CataniBernd Mueller-RoeberMartina ChiriacòBernd NürnbergMartina GentzschBernd StegmayrMartina HöcknerBernd StratmannMartina KleberBernd W. SiguschMartina KroppBernd WissingerMartina LeopizziBernhard BiersackMartina M. ZaninottoBernhard GibbsMartina MihaljBernhard H. RauchMartina PasquaBernhard HuchzermeyerMartina PeršeBernhard KrismerMartina PfefferBernhard LollMartina PiccoliBernhard ReschMartina PichrtovaBernhard SchusterMartina PigazziBernhard SpinglerMartina ŘezáčováBernhard ZelgerMartina SalákováBert LittleMartina SamiotakiBerta Cillero-PastorMartina SchmidtBertal H. AktasMartina Šeruga MusićBerthold DrexlerMartina ŠpundováBerthold HuppertzMartine CrosetBertold RennerMartine CultyBertram FlehmigMartino PengoBertrand D. E. RaynalMarvin DiazBertrand LiagreMarwa MatboliBerzenji LawekMary Anna VenneriBeth A. BouchardMary BeilbyBeth MallardMary Cloud B. AmmonsBethany A. Buck-KoehntopMary Frances McMullinBettina BlaumeiserMary GulumianBetty C. A. M. Van EschMary KavurmaBetul Aldemir DikiciMary Lou CutlerBeverly A. TeicherMary N. SheppardBhagirath ChaurasiaMaryam ArdalanBhagwat NawadeMarye Boers-SonderenBhagyaraj EllaMaryline Magnin-RobertBhakti PrinsiMary-Lorène GoddardBhama RamkhelawonMaryna BasalayBharani SrinivasanMaryna Krawczuk-RybakBharathiraja SubramaniyanMaryna SkokBhasker RadaramMarzena LaskowskaBhaswati BhattacharyaMarzena MackowiakBhawik Kumar JainMarzenna BlonskaBhesh Raj SharmaMarzia BianchiBhola PradhanMarzia Di DonatoBhola Shankar PradhanMarzia OgnibeneBhupesh SinglaMarzia PucciBhupinder SinghMarzia SeguBiagio RaponeMarzia VergineBiagio SantellaMaša Skelin KlemenBianca ColonnaMaša ŽdralevićBianca FurduiMasaaki ItoBianca NitzscheMasaaki KonishiBianca R. AlbuquerqueMasafumi NodaBianca SclaviMasafumi TanakaBianca VezzaniMasahiro FunahashiBiancamaria CiascaMasahiro IkedaBiancamaria SenizzaMasahiro ImamuraBiao ZengMasahiro IwamotoBibhash C. PariaMasahiro KonoBibi KhanMasahiro MaruyamaBice ContiMasahiro MiyashitaBidhan BandyopadhyayMasahiro SeikeBidisha RoyMasahiro YamadaBieneke JanssenMasahito IshikawaBijan GhalehMasakatsu WatanabeBijay DhungelMasakazu AtobeBijayalaxmi MohantyMasakazu IshiiBiju ThomasMasakazu NamihiraBikash PattnaikMasakazu SugishimaBilal Alashkar AlhamweMasaki KimuraBilha FischerMasaki KobayashiBiliana Lozanoska-OchserMasaki MogiBiljana Đ. GlišićMasaki UenoBiljana MojsoskaMasami NakajimaBill DennyMasami NozakiBimal JanaMasami SekineBimal KoiralaMasami YoshimuraBin GuMasamichi IshiaiBin LinMasamichi KamihiraBin QiuMasamichi NagaeBin WangMasanobu MinoBindu ChandrasekharanMasanobu NannoBing SunMasanori AritaBing ZhuMasanori FukushimaBingxian XieMasanori KawakamiBinu Kandathilparambil SasiMasanori KuriharaBiplab SarkarMasao KodaBirandra K. SinhaMasaomi IkedaBirol CabukustaMasaru MatsudaBiserka RelicMasaru TanakaBishal PaudelMasashi MizuguchiBisheng ZhouMasataka AmisakiBishnubrata PatraMasato SatoBishoy El-AaragMasato T. KanemakiBi-Shuang ChenMasatoshi MakiBiswarup SahaMasatoshi SuzukiBjarke FollinMasayasu KuwaharaBjoern TitzMasayasu MieBjorn BaseletMasayuki FujitaBjörn KarlssonMasayuki ItohBjörn KoosMasayuki SatakeBjörn TampeMasayuki TakahashiBlake HildrethMasayuki TsujimotoBlanca Elí Ocampo-GarcíaMasood SepehrimaneshBlanca MolinsMasoumeh OzmaianBlaž LikozarMassimiliano BonifacioBłażej KudłakMassimiliano CadamuroBo DongMassimiliano EspositoBo SunMassimiliano FambriniBo WangMassimiliano ManciniBo Young ChungMassimiliano RuscicaBoas PuckerMassimiliano TognoliniBoaz BarakMassimo AureliBob LubambaMassimo BergerBodhisattwa BanerjeeMassimo BottiniBodnar RichardMassimo BusinBodo LinzMassimo CarossaBodo PhilippMassimo ColettaBo-Eun YoonMassimo ConeseBogdan BatkoMassimo Delle PianeBogdan BujnickiMassimo LibraBogdan CatalinMassimo MaccagnoBogdan DorofteiMassimo MarielloBogdan Gabriel ȘlencuMassimo MariottiBogdan Ionel TambaMassimo MarrelliBogdan IorgaMassimo ReconditiBogdan KazmierczakMassimo ReverberiBogdan KontekMassimo RussoBogdan O. PopescuMassimo SalviBogdan Silviu UngureanuMassimo TrottaBoguslawa LuzakMassimo UbaldiBogusława PietrzakMassimo VendittiBogusz TrojanowiczMassimo ZeraniBohumil FafilekMasumi IshibashiBohuslava TremlováMáté VargaBojan KnapMatea Nikolac PerkovicBojan KozlevčarMateja GermBojana VidovicMateusz DaśkoBojin BojinovMateusz KarniaBojjibabu ChidipiMateusz LabuddaBokyung SonMateusz MaciejczykBolesław KalickiMateusz MajdaBoleslaw KarwowskiMateusz MikiewiczBolisetty SreenathMateusz PsurskiBomi RyuMateusz PuśleckiBonaccorsi Di Patti Maria CarmelaMateusz SikoraBongju KimMateusz SpałekBonnie FiresteinMathew BloomfieldBonnie L. FiresteinMathews VargheseBony De KumarMathias MontenarhBoon Seng WongMathias TessonBoris ErshovMathieu Di MiceliBoris I. KurganovMathieu EdelyBoris MargulisMathieu GautierBoris ShivachevMathieu J. F. CrupiBoris SkryabinMathilde Body-MalapelBoris TenchovMathu C. MalarBoris VauzeillesMati KarelsonBorislav AngelovMatias AvilaBorislav KovačevićMatias MorinBorja Belda PalazonMatías MorínBorja Belda-PalazónMatias MosqueiraBorja Herrero De La ParteMatic PavlinBor-Show TzangMatjaz RokavecBorutaitė VilmantėMatouš GlancBoryana PetrovaMats H. T. TroedssonBorys OśmiałowskiMats LeifelsBöttcher BettinaMats-Olof MattssonBouchta SahraouiMatt D. JohansenBoudewijn Van Der SandenMatteo A. SurdoBoya WangMatteo AudanoBoyan K. GarvalovMatteo BiaforaBoyong KimMatteo BrucoliBożena BukowskaMatteo Bruno LodiBožena CukrowskaMatteo ButiBożena Futoma-KołochMatteo CalcagnileBrad BarnettMatteo Cotta RamusinoBradford K. BergesMatteo FabbriBradley A. FrekingMatteo FerroBradley BakerMatteo FlorisBradley ElliottMatteo GiovarelliBradley FergusonMatteo GoldoniBradley LaunikonisMatteo GuidottiBradley TaylorMatteo MorettiBrajesh Kumar SinghMatteo NardinBrandon HendersonMatteo PaciniBrandon Lucke-WoldMatteo SorgeBrandon Mark GassawayMatteo TegoniBranislav BrutovskyMatthäus FelsensteinBranislav KuraMatthew BeardBranislav ŠilerMatthew D. BensonBranka MarinovicMatthew D. BergBranka Salopek-SondiMatthew E. CallBrankica JankovicMatthew F. RouhierBrankica MravinacMatthew G. K. BeneschBranko ZevnikMatthew GageBratislav MarinkovićMatthew J. AndersonBratko FilipičMatthew L. FaronBrázda VáclavMatthew Lewis BendallBrenda KwakMatthew LincolnBrenda M. AlexanderMatthew McIntoshBrenda Y. LeeMatthew P. PadulaBrendan C. Stack, Jr.Matthew R. MauldinBrendan HoustonMatthew StewartBrent A. WilliamsMatthew TaylorBreznik BarbaraMatthias BacheBrian Andrew Van TineMatthias BoentertBrian BeliveauMatthias CarlBrian CeresaMatthias Christian VoggBrian D. AdamsMatthias David ErlacherBrian D. WisendenMatthias DerwallBrian DixonMatthias GatzBrian GabrielliMatthias Hardtke-WolenskiBrian J. AneskievichMatthias LauthBrian J. MitchellMatthias MeinhardtBrian J. NorthMatthias MillesiBrian J. ScottMatthias NeesBrian McKayMatthias PortBrian PiperMatthias ReigerBrian S. CummingsMatthias SchumacherBrian SuttonMatthias SteinBrigitte HantuschMatthias ThalmannBrigitte Le Magueresse-BattistoniMatthias UllrichBrigitte MarianMatthias WilmBrijeshkumar PatelMatthias WolfBritt OpdebeeckMatthieu CaruelBritt WildemannMatthieu SainlosBrittany Allen-PetersenMattia BartoliBrittney HarringtonMattia BernettiBrock HumphriesMattia BraminiBronisława Skrzep-PoloczekMattia D′AgostinoBruce DurieMattia Di PaoloBruce SpittleMattia Falchetto OstiBruna PanizzuttiMattia ManicaBrunetti DarioMattia MoriBrunno Rocha LevoneMattia QuattrocelliBruno BeaumelleMattia RiefoloBruno Bueno-SilvaMattia Russel PantaloneBruno ConstantinMattias AnderssonBruno Costa GomesMatyas FendrychBruno CozziMátyás MeggyesBruno DallagiovannaMaud A. W. HermansBruno DusoMaud DumouxBruno FaveryMaud FriedenBruno FiondaMaura R. Z. RuzhnikovBruno FonsecaMaurice J. B. Van Den HoffBruno ImbimboMauricio Alcolea PalafoxBruno Jorge ColaçoMauricio ErbenBruno L. CadilhaMauricio MoraisBruno LaganàMaurizio BadianiBruno Manuel Galinho HenriquesMaurizio BrigottiBruno MeloniMaurizio CavalliniBruno Miguel Reis Da FonsecaMaurizio EliaBruno PaganoMaurizio ForteBruno R. B. PiresMaurizio MargaglioneBruno TassoMaurizio MartiniBruno VictorMaurizio MulasBruno VilloutreixMaurizio NordioBryan BaileyMaurizio OnistoBryan BjorkMaurizio OrlandiniBryan D. CrawfordMaurizio PaciBryan FullerMaurizio PolanoBryan GonzálezMaurizio RagniBryan J. MathisMaurizio ScarpaBryn D. MonneryMaurizio SoriceBuddhadev LayekMaurizio TaurinoBuHyun YounMaurizio VentreBulat RamazanovMauro AlaibacBülent ErginMauro BelliBurkhard BrandtMauro ColucciaBurkhard H. Burkhard H. PoeggelerMauro DacastoBurkhard KleuserMauro FinicelliBurkhard MöllerMauro Francesco SgroiBurkhard SchulzMauro GiorgiBuyun TangMauro GiuffrèByeong Hwa JeonMauro MontalbanoByeong-Ui MoonMauro PatroneByeong-Wook SongMauro SalviByeongwoon SongMauro VaccarezzaByoung Hun LeeMax E. JoffeByron BaronMax K. LeongByron CaugheyMax PiffouxByung Eui KimMax RyadnovByung Seok MoonMaxim L. BychkovByung Soo KimMaxim S. KokoulinByunghee HanMaxim Stanislavovich MakarenkoByung-Hoon JeongMaxime KlausenByungsuk KwonMaximilian SeidlC. Galindo-RomeroMaximilian WenigerC. Henrique AlvesMayank Anand GururaniC. Leigh BroadhurstMayank ChoubeyC. PedrazaMayra PaolilloCaitlyn BowmanMays TalibCale D. FahrenholtzMaytham HusseinCălin CăinapMayumi KomineCalin Mircea GhermanMayur ChoudharyCalla M. GoekeMayura MeerangCalogero FioricaMayura VeeranaCambón AdrianaMazahar MoinCamelia QuekMd A. HakimCamelia Sorina StancuMd Abdul HannanCamelia UngureanuMd AdnanCameron MuraMd Ashik UllahCameron P. BrackenMd Ashraf HossainCamila D. Madeira CamposMd Ashraful Islam MollaCamilla MargaroliMd AshrafuzzamanCamilo López-AlarcónMd Habibur RahmanCamilo López-CristoffaniniMd JakariaCan CuiMd Jamal UddinCandela CuestaMd Kausar RazaCandice Z. UlmerMd Mamunul HaqueCândida Teixeira TomazMd Moshfekus Saleh-e-InCankui ZhangMd Nazmul IslamCankut ÇubukMd Shiblur RahamanCara J. WestmarkMd Tabish NooriCarina BalcoşMd Tahjib-Ul-ArifCarina MalmhällMd Tohidul IslamCarine MichielsMd Toufiqur RahmanCarine TisnéMeaurio EmilianoCarl D. BortnerMedha PriyadarshiniCarl ErbMeehyein KimCarl JohnsonMeena JaggiCarla BoitaniMeerambika MishraCarla CaffarelliMegan BestwickCarla CannizzaroMegan E. Rosa-CaldwellCarla CicalaMegan J. PuckelwartzCarla CruzMegan KeniryCarla GasbarriMegan L. StaniferCarla MucignatMegan PennoCarla PeregoMegan SpurgeonCarla PollastroMeganne ChristianCarla RaggiMeghali NighotCarla S. SantosMeghan RebuliCarla SantosMehboob SheikhCarla SilvaMehdi BenkahlaCarla VerriMehdi DamaghiCarles ArúsMehdi ShakibaeiCarles Díez-LópezMehmet M. AltintasCarlie De VriesMehran DastmalchiCarlo AprileMehul VoraCarlo Augusto MallioMei Lan KoCarlo C. VersacéMei Shan LiewCarlo Cosimo CampaMei-Chin LuCarlo DiaferiaMeicun YaoCarlo Ettore PellicciariMei-hwa LeeCarlo GalliMeik NeufurthCarlo GaniniMeira MachadoCarlo IraceMeiyun FanCarlo MorelliMejía CarmenCarlo MusioMelania MelisCarlo PozziMelanie DeutschCarlo ReggianiMélanie IkehCarlo SantambrogioMelanie MärklinCarlo TicconiMelchor Sanchez-MartinezCarlo ViscomiMelinda Erzsébet TóthCarlos A. MazzaMelinda KolcsárCarlos A. SalgueiroMelinda SzilágyiCarlos AydilloMelis OlcumCarlos CaroMelissa Agsalda-GarciaCarlos CondeMelissa BarberCarlos EscalanteMelissa BowermanCarlos EscobedoMelissa D. CantleyCarlos FarinhaMelissa MaczisCarlos Fernando MourãoMelissa MagerøyCarlos Fontes RibeiroMelissa MarianaCarlos GarridoMelissa PerreaultCarlos González-FernándezMelissa SantiCarlos GuardiaMelissa VosCarlos Guerrero-SanchezMelkam KebedeCarlos Gutierrez-MerinoMelvin LeowCarlos LeonMendell RimerCarlos M. CorreiaMeng LiuCarlos M. MartínezMeng MaoCarlos Martins GomesMeng ZhuCarlos MedinaMengcheng ShenCarlos Miguel MartoMenghsiao MengCarlos OsorioMeng-Tsan ChiangCarlos PalmeiraMengyuan ChenCarlos Perez-StableMenno HoekstraCarlos Plaza-SirventMeran Keshawa EdiriweeraCarlos RamosMercè Pallàs LliberiaCarlos RosadoMercedes Del-Rio CelestinoCarlos Sanz-GarciaMercedes FernandezCarlos VillalobosMercedes Garcia-GilCarlotta PontremoliMercedes JiménezCarme FabregaMeritxell García-QuintanillaCarmela FuscoMeritxell MunmanyCarmela ParentiMeritxell PerramónCarmela RicciardelliMetello InnocentiCarmela SantangeloMetis HasipekCarmela SaturninoMetka NovakCarmelo BellarditaMia HorowitzCarmelo BernabéuMiao FeiCarmelo Carmona-RiveraMicaela GliozziCarmelo GurnariMicaela MorelliCarmelo Maria MusarellaMichael A. FreitasCarmen AaneiMichael A. ShestopalovCarmen AdriaensMichael AngastiniotisCarmen CapelMichael ArulCarmen CerchiaMichael B. PapahCarmen ChifiriucMichael BanfCarmen Cristina DiaconuMichael BoschmannCarmen De MiguelMichael BowmanCarmen DinizMichael BukrinskyCarmen EspinósMichael C. BurgerCarmen Fiuza-LucesMichael CangkramaCarmen Guillén-PonceMichael CarrollCarmen J. Pastor-MaldonadoMichaël CerezoCarmen Lăcrămioara ZamfirMichael CharetteCarmen MannellaMichael ChvanovCarmen Maria García-PascualMichael CrossCarmen Nussbaum-KrammerMichael D. LockshinCarmen RamosMichael DoudCarmen RinaldiMichaël DussiotCarmen RivasMichael E. BoultonCarmen VillagrasaMichael E. JohnsonCarmine FinelliMichael E. SternCarmine IzzoMichael E. SymondsCarmine MerolaMichael E. VaydaCarmine SelleriMichael EllisonCarol WadhamMichael FasulloCarola BerkingMichael FieldCarola J. MaturanaMichael FraserCarole HéniqueMichael GrantCarole SousaMichael H. PerlinCarolin KolmederMichael HesseCarolin MoglerMichael HowardCarolina ArmengolMichael HsaioCarolina BalbiMichael HutterCarolina HagbergMichael IbbaCarolina MehaffyMichael J. B. KutrykCarolina Munos-CamargoMichael J. McclureCarolina PellegriniMichael Jacob IoelovichCarolina Susana CerrudoMichael John SchuellerCaroline DunkMichael KlutsteinCaroline Le MaréchalMichael KravchikCaroline MarconMichael KreisslCaroline S. MurphyMichael LalkCaroline SmithMichaël LaurentCarrie Mae LongMichael LenhardCarrie Ris-StalpersMichael LichtenauerCarrie ShawberMichael MakCarsten CarlbergMichael MildnerCarsten GrötzingerMichael NiederwangerCarsten HoffmannMichael OlaussonCarsten HöltkeMichael PeresCarsten SachseMichael PerlinCarsten SchulteMichael PuschCarsten TheissMichael R. DoresCasas FrançoisMichael R. FettiplaceCasey NestorMichael R. LadomeryCasey R. VickstromMichael RisnerCaspar Grond-GinsbachMichael RossCassandra KohMichael RothCassie S. MitchellMichael ShtutmanCatalin LazarMichael ShurinCătălin MaximMichael SigalCatalin ZahariaMichael StearCatalina Cuellar-GempelerMichael TellierCatalina Hernández-SánchezMichael TranterCatalina Natalia Cheaburu YilmazMichael TsangCatalina PislariuMichael VolnyCatalina Valdés-BaizabalMichael W. BeckCatarina E. HioeMichael WiggsCatarina RamosMichael WilsonCatarina SeabraMichael X. HendersonCatarina SilvaMichael X. ZhuCate SpeakeMichaela FiliouCatello Di MartinoMichaela RumlováCaterina CiacciMichaela Schulz-SiegmundCaterina LiciniMichaela ŠiklováCaterina MancarellaMichail KlontzasCaterina MannaMichail MichailidisCaterina Maria GambinoMichail RovatsosCaterina PeggionMichał ArabskiCaterina PipinoMichał BiernackiCaterina SoldàMichał BijakCaterina SquillaciotiMichal BittnerCatherine CahillMichal BoreckiCatherine CoiraultMichał BulcCatherine DamervalMichał BurdukiewiczCatherine DavisMichał CaputaCatherine DuezMichał CegłowskiCatherine J. WalshMichal HetmanCatherine KernMichal J. DabrowskiCatherine LéonMichal JesetaCatherine O′NeillMichal JurášekCatherine RayonMichal K. StachowiakCatherine TeyssierMichal KaradyCatherine ThorntonMichal Krystian OklinskiCatherine ViguiéMichal KuncCatherine YzydorczykMichal LetekCathleen R. CarlinMichal LinialCatia LambertucciMichał MączewskiCatia ScassellatiMichal MegoCaudai ClaudiaMichal NiemczakCécile MartinatMichal NowickiCécile RaynaudMichał OczkowskiCécile VignalMichal OrdakCecilia BucciMichał OtrębaCecilia GelfiMichał PopowCecilia ManciniMichal ŘezankaCecilia Matilde Fernández PonceMichal SantockiCecilia OseraMichal SmahelCecilia PozziMichał SzlisCecília R. A. SantosMichal WozniakCecilia VecoliMichał ZałȩckiCédric DelevoyeMichal ZemanCees A. SwenneMichał ZimeckiCees NoordamMichalis GeorgiouCeferino Carrera FernándezMichalis LiontosCélia Kun-RodriguesMicheal A. McLellanCélia Maria Cássaro StrunzMichel CamploCélia MiguelMichel HananiaCeline BerthierMichel HanssCeline FisetMichel J. A. M. Van PuttenCeline GongoraMichel KranendonkCerrone CabanosMichel LongyCésar A. LopezMichel R. PopoffCésar Augusto Correia De SequeiraMichel RohmerCésar Calvo-LoboMichel RonjatCesar CatalánMichel SèveCesar Lopez-CamarilloMichel SimonCésar Márquez-QuirozMichela BattistelliCésar MatteiMichela CastagnaCesar NunezMichela CorsiniCesare AccinelliMichela Di NottiaCesare IndiveriMichela FerrucciCéu CostaMichela GabelloniCezary CzaplewskiMichela IannoneCezary SempruchMichela M. GiulianoChadwick C. ProdromosMichela PozzobonChaeuk ChungMichela RigoniChaido SirinianMichela RossiChaitanya ValivetiMichela RugoloChakradhara Rao UppugunduriMichela SchiavonChalermchai KhemtongMichela ZuffoChalid AssafMichelangelo La SpinaChan Kuan RongMichele ArcopintoChan Yeong HeoMichele BenedettiChan Young ShinMichèle CaragliaChanchal MandalMichele CrottiChanchao LorthongpanichMichele Crozatier-BordeChandler L. WalkerMichele D′AngeloChandra BoosaniMichele FabrazzoChandra Sekhar BathulaMichele FornaroChandra Shekhar PareekMichele Francesco Maria SciaccaChandrakala Aluganti NarasimhuluMichele GiulianiChandrakanth EdamakantiMichele GraciottiChandrashekhar VoshavarMichele LaiChang GuoMichele LanzaChang Ho KangMichele MaffiaChang Hoon LeeMichele MalaguarneraChang LiaoMichele MondiniChang Pyo HongMichele NavarraChang Young JangMichele OrtolaniChang-Gue SonMichele P. LambertChang-Han ChenMichele PaolantonioChang-Hao YangMichele PapaChanghong WangMichele PinelliChanghoon ChoiMichele SamajaChang-Hoon WooMichele SavianoChanghwan AhnMichele TonelliChang-Hyu BaeMichele VecchioChangjong MoonMichelle DaCostaChangling HuMichelle GarciaChang-Ro LeeMichelle GruninChangseok HanMichelle K. Y. SiuChangseon RyuMichelle L. ManniChang-Shen LinMichelle M. Martínez-MontemayorChang-Sik OhMichelle McClementsChang-Suk KongMichelle Starz-GaianoChang-Tze YuMichiharu NakanoChanKyu KangMichinari KohriChanpreet SinghMichio AsahiChantal HoueeMichio SzukiChantal VauryMichiyo SuzukiChao LiMicholas Dean SmithChao lien LiuMick WellingChao TanMickael BlaiseChao ZhaoMieczyslaw MakoszaChao-Hui YangMiguel A. AlonsoChao-Lin LiuMiguel A. Garcia-GonzalezChaoling ChenMiguel A. OrtegaChaoqun YaoMiguel A. PrietoChao-Yie YangMiguel AlcarazChaozhong ZhangMiguel Angel Alcántara-OrtigozaChara SimitziMiguel Ángel Piris PinillaCharalampos SiristatidisMiguel Ángel PrietoCharalampos SpilianakisMiguel Ángel SolerCharat ThongprayoonMiguel Armengot-CarcellerCharikleia StefanakiMiguel BurgosCharith Raj Adkar-PurushothamaMiguel Cacho TeixeiraCharles Antwi-BoasiakoMiguel CarracedoCharles Bou-NaderMiguel HuertaCharles C. LeeMiguel HuesoCharles H. OppermanMiguel IdoateCharles HocartMiguel MontoroCharles L. WilkinsMiguel MoutinhoCharles PiletteMiguel PrietoCharles RedwoodMiguel SantosCharles RinaldoMiguel Toro GonzalezCharles S. HiiMiguel Vicente-ManzanaresCharles ShulerMiha MoškonCharles T. HunterMihaela AvadaneiCharles W. SchindlerMihaela BalasCharlotte DahlemMihaela Bustuchina VlaicuCharlotte PoschenriederMihaela GhitaCharlotte Von GallMihaela MoscaluCharng-Cherng ChyauMihaela R. PopescuCharu KothariMihaela TurtoiChathura SuraweeraMihaela TurturicăChee Wah TanMihai CenariuChe-Hsin LeeMihai MoldovanCheil MoonMihai Stefan MuresanChe-Kun James ShenMihail Lucian BirsaChellappagounder ThangavelMihail MitovChen Huei LeoMihajlo EtinskiChen-Chi WuMihnea DragomirCheng WangMihnea-Alexandru GamanCheng-An LinMiika MartikainenChengheng LiaoMika SalmiCheng-Huan ChenMika SugaCheng-I LeeMikael BroscheChengji ZhouMike BarbeckChenglei FanMike DoddChenglin MoMike DrewCheng-Maw HoMike LeVineChengmin JiangMike SathekgeCheng-Ping YuMikel SanchezCheng-Shun LuMikelis KirpluksChengwen SunMiķelis KirpļuksCheng-Yang ChouMikhail A. FilyushinCheng-Yang HuangMikhail A. LiskovykhCheng-Yen KaoMikhail AlexeyevChengyue WuMikhail ChumakovChenjiang YouMikhail DivashukChenshuang LiMikhail DrobizhevChenyu HuangMikhail DubininChenyu SunMikhail G. AkimovChen-Yuan LinMikhail GantsevichCheolju LeeMikhail I. KusaykinCheol-Su KimMikhail KarushevCheong Rae RohMikhail KislinCher-Rin ChongMikhail M. MaslovCheryl CeroMikhail M. Vorob′evCheryl GariepyMikhail MaslovCheryl KlaimanMikhail N. KolotChetan MeshramMikhail PyatnitskiyChetasi TalatiMikhail ShamoninChetna SoniMikhail SolovievCheuk Lun LiuMikhail V. DubininChi Bum AhnMikhail Y. InyushinChi Chun WongMiki KushimaChi Keung LamMikihisa UmeharaChi MaMikihito KajiyaChi ZhouMikihito TakenakaChia-Che HoMikio HayashiChia-Chen ChangMikko O. LaukkanenChia-Chi LiuMiklós ErdélyiChia-Chia ChaoMiklós KecskésChiachung ChenMiklós LengyelChia-Hsien FengMiklós NagyChia-Hung ChouMiklós PoórChia-Hung LeeMilad HadidiChia-Hwa LeeMilad PourbaghiChia-Jung ChangMilagros BuenoChian Ju JongMilan JakubekChiang-Ting ChienMilan Kumar LalChianju JongMilan SencanskiChianshiu ChienMilana Frenkel-MorgensternChiao-En WuMilena CavicChiara ArrigoniMilena DeptułaChiara BardellaMilena G. GeorgievaChiara BarisioneMilena GeorgievaChiara BattistiniMilena NasiChiara BazzichettoMilena PanticChiara BiselliMilena PawChiara BrulloMilena PetriccioneChiara CervettoMilena ŚciskalskaChiara CipollinaMilena ŚwitońskaChiara CortiMilica BorovcaninChiara Di LorenzoMilica BozicChiara Di RestaMilki RahatChiara Di TucciMillie Rincón-CortésChiara FerraciniMilorad VujicicChiara GaiMilos FilipovicChiara GamberiMiloš HricovÍniChiara GiacomelliMiloslava FojtováChiara Maria MottaMilva PepiChiara MartinelliMin Gi JoChiara PagliaraniMin ParkChiara PlatellaMin Sang KwonChiara PorroMin XueChiara PosarelliMin Young LeeChiara RossoMina LeeChiara RuoccoMina PopovicChiara SorberaMinas M. StylianakisChiara ValoriMîndra Eugenia BadeaChiara VillaMindy I. DavisChiara VitaleMindy LevineChiara VolaniMinee ChoiChia-Wei ChengMinerva Codruta BadescuChia-Wen TsaiMing ChenChia-Yang LiMing Feng LiaoChia-Yih WangMing Hsien ChanChia-Yu ChuMing Jay DengChi-chang JuanMing LiChichun HoMingbo WangChien-Chin ChenMing-Che LiuChien-Chung HuangMing-Cheng ShihChien-Chung YangMing-chieh MaChien-Feng LiMinghua WuChien-Jui HuangMing-Ju HsiehChien-Liang ChenMing-Kuei ShihChien-Min ChiangMingqing LiChien-Min LinMingsheng ChenChien-Ming ChaoMing-Shiu HungChien-Sen LiaoMing-Tsun TsaiChien-Yuan LinMing-Tzu TsaiChi-Fen ChangMingxing QianChih Hung LoMing-Yan CheungChiharu OtaMing-Yi ShenChih-Chen HsiehMingzhuo LiChih-Cheng ChenMinh-Thu NguyenChih-Chia SuMinh-truong DoChih-Chin YangMin-Hua ChenChih-Ching LinMinhui ChenChih-Feng HuangMinjung SongChih-Hao LuMinkui LuoChih-Hao WangMinli XingChih-Hsin TangMinoo RassoulzadeganChih-Hsun LinMinoru KobayashiChih-Hung GuoMinoru MiyazatoChih-Jen YangMinoru TakasatoChih-Li LinMinoYang YangChih-Liang WangMin-Soo KimChih-Min WangMinyu ChungChih-Po ChiangMioara LarionChihuei WangMiquel MuleroChih-Wei ChenMiquel NadalChihyang WangMira A. M. BehnamChih-Yao HouMiran RadaChih-Yen ChienMiranda MeleChih-Yen KingMiranda Sin-Man TsangChii-Ming LeeMircea LeabuChi-Ju KimMircea TampaChi-Ming WongMireia AlcaldeChin LiMireia López-SilesChin Yan LimMireia MallandrichChin-Chuan ChenMireille VerdierChin-Chuan HungMirela Baus LončarChing Feng WengMirela Cleopatra TomescuChing-Hsi HsiaoMirela MatićChing-Hu ChungMirella GiovarelliChing-Jin ChangMiri KrupkinChing-On WongMiriam AyusoChing-Yi TsaiMiriam EchevarriaChing-Yu LeeMiriam HoeneChing-Yun ChenMiriam PotronyChinh Tran-To SuMiriam SciaccalugaChin-Kun WangMiriam-Rose MenezesChin-Man WangMirjam BrackhanChinmay SatamMirjam Jeanette EberhardtChin-Mu HsuMirjana Babić LekoChin-Wei HuangMirjana JerkicChi-ping DayMirjana LiovicChirag GajjarMirka SaarelaChirag VasavdaMirko ManchiaChiranjeevi KorupalliMirko ManettiChi-Rei WuMirko VoelkersChirstopher HanleyMiroslav BarancikChisato KinoshitaMiroslav KvasnicaChitra MandyamMiroslav SrbaChiu-jung HuangMiroslava KačániováChi-wai KanMiroslava ZhiponovaChi-Yu LuMirosław GiurgChloé JournoMirosław KwiatkowskiChloe MartensMirosław S. BonekCho Yeow KohMirosława ChwilCho-Hao LeeMiroslawa CichorekChong-Tai KimMiroslawa DabertChoong-Gu LeeMirosława Ferens-SieczkowskaChoong-ill CheonMirta MilicChoyi ChenMiruna StanChris BurtMiryam Criado GonzalezChris BusbyMiryam MullerChris J. L. M. MeijerMirza BojićChris JonesMirza HasanuzzamanChris MacDonaldMisa HayashidaChris MichielsMisa TakahashiChris Van Der PoelMi-Suk SeoChris YoungMiszalski ZbigniewChrishan RamachandraMithilesh JhaChrista BuechlerMitra Safavi-NaeiniChristel MarquetteMitsuhiro MoritaChristel VangestelMitsuko MasutaniChristelle Adam-GuillerminMitsuru NakazawaChristelle CauffiezMitsuru TashiroChristelle CorauxMitsutaka KitamuraChristelle DantecMittal JasoliyaChristelle GuibertMitura ZbigniewChristelle Le FollMiwako Kato HommaChristelle MarminonMiwako NishioChristelle MonvilleMiyako TanakaChristiaan HenkelMizuho NakayamaChristian Albrecht MayMizuki YamamotoChristian AlzheimerMladen JergovicChristian BaillyMladen ParadžikChristian BeltonMobashwer AlamChristian BleilevensMobeen Ur RehmanChristian D. YoungModesto Redrejo RodriguezChristian De La FeMoe ThuzarChristian Di BuduoMohamad AssiChristian EhltingMohamed A. El-EsawiChristian FercherMohamed Al-FatimiChristian GorzelannyMohamed AliChristian HiepenMohamed El-MeseryChristian KosanMohamed HassanChristian LehmannMohamed M. Abdel-DaimChristian M. JulienMohamed MutalibChristian MandryckyMohamed Raafat El-GewelyChristian MargreitterMohamed-Amine HassaniChristian MayerMohammad Alinoor RahmanChristian NapoliMohammad AlzrigatChristian OpländerMohammad Amjad KamalChristian P. MüllerMohammad Asif SherwaniChristian PehamMohammad El KhatibChristian PiflMohammad Faujul KabirChristian ReynoldsMohammad Ferdous MehbubChristian RudolphMohammad HasanChristian SahlmannMohammad Hassan BaigChristian Schmitz-LinneweberMohammad Hassan HodrojChristian SchwerkMohammad Hojjat-FarsangiChristian SmolleMohammad Imran KhanChristian Sordo-BahamondeMohammad IrfanChristian StockMohammad K. HassanChristian TantardiniMohammad Shah JahanChristian Tellgren-RothMohammad Tavakkoli YarakiChristian WirajaMohammadreza NaeimiradChristian WrightMohammadreza ShokouhimehrChristiane BranlantMohammed A. RohaimChristiane Charriaut-MarlangueMohammed BarigouChristiane KlecMohammed GagaouaChristiane Laranjo SalgadoMohammed K. HankirChristiani AmorimMohammed L. AbbaChristilla Bachelot-LozaMohan AmarasiriChristina B. KarstenMohan MaheshChristina C. TamMohanraj SadasivamChristina CastellaniMohaseen TamboliChristina E. KostaraMohd AsgherChristina GrimmMohd SaeedChristina LamersMohd Saleem DarChristina LobergMohd ShahbaazChristina MertensMohit BibraChristina OrrúMohsen Falahati AnbaranChristina PacakMohsen HesamiChristina SchroederMohsen KarimiChristina TangMohsen MazidiChristine BeuckMohsen SheikholeslamiChristine E. EngelandMohsin JafriChristine E. GeeMohsin KhanChristine HappelMoises Di SanteChristine HeberdenMoises Leon-JuarezChristine HellwegMoisés Rodríguez-MañeroChristine KriegerMojca Čakić SemenčićChristine Le RoyMojca KržanChristine MehnerMojmir MachChristine PourcelMojtaba VaismoradiChristine RanggerMokarram M. HossainChristine RondaninoMona FothChristine SchmidtMona I. ShaabanChristine WhiteMona M. FreidinChristo KoleMonglien WangChristof BernemannMonia PeruginiChristof LenzMónica Álvarez-FernándezChristoforos NikolaouMonica CalasansChristoph BurckhardtMonica CanepariChristoph CastellaniMonica CantileChristoph GarbersMonica CarmosinoChristoph JochumMonica Cartelle GestalChristoph KaetherMonica CiveraChristoph ReinhardtMonica CurròChristoph RiethmüllerMonica De CaroliChristoph SchwarzerMonica HârțaChristoph ViebahnMónica Lamas MaceirasChristophe BoscMonica MencarelliChristophe CauxMonica MoniciChristophe CullinMonica MontagnaniChristophe DeroanneMonica MottesChristophe GaillochetMonica MusteanuChristophe HanoMonica SalamoneChristophe OrssaudMónica SerranoChristophe PellefiguesMonica ValentovicChristophe RavaudMonika BartekováChristophe ThirietMonika Christine Brunner-WeinzierlChristopher A. WassifMonika CzerwinskaChristopher CampbellMonika Ehling-SchulzChristopher ClarkeMonika FurkoChristopher D. LinkMonika GawałkoChristopher D. ZahmMonika GjorgjievaChristopher DawsonMonika HämmerleChristopher DonnellyMonika HertenChristopher H. EskiwMonika HolubovaChristopher HenstridgeMonika JaneczkoChristopher J. AlteriMonika Kadela-TomanekChristopher J. RidoutMonika KijewskaChristopher KobierzyckiMonika KoziełChristopher Lyon BrownMonika NaumowiczChristopher MakaroffMonika Ostaszewska-BugajskaChristopher MoertelMonika PawłowskaChristopher P. ToselandMonika PruensterChristopher PeacockMonika Sakowicz- BurkiewiczChristopher R. M. AsquithMonika SkorupaChristopher VeldkampMonika SobočanChristopher WassonMonika SzturmowiczChristos ChadjichristosMonika TrzcińskaChristos ChatzidoukasMonika TulejaChristos GeorgiouMonika WojciechowskaChristos K. KontosMonique Courtade-SaïdiChristos KissoudisMonique M. A. VerstegenChristos PapaneophytouMonirul IslamChristos StournarasMon-Juan LeeChrong-Reen WangMonokesh Kumer SenChryssostomos ChatgilialogluMonserrat López-SanmartínChuan Chiang-NiMonssef Drissi-HabtiChuan LiMonsurat LawalChuanbin MaoMontecucco FabrizioChuang-Rung ChangMontserrat ColillaChuan-Lin ChenMontserrat Esteve RàfolsChuanqi PengMontserrat GarcíaChuanxiong NieMonzurul RoniChulhun KangMood MohanChul-Su YangMoo-Ho WonChun ChenMoon Il KimChun-Chi WuMoonkyoo KongChunchia ChengMootaz SalmanChunfu XuMorag LewisChung WongMorgan HamonChung-Der HsiaoMori ShuichiChung-Feng HwangMorifumi FujitaChungho LauMorihito TakitaChung-Hsi HsingMoriyama MitsuakiChung-Hsin WuMorris MaduroChung-Hwan ChenMorris ManolsonChung-Jiuan JengMorshed KhandakerChung-Jung LiuMortaza KhodaeiaminjanChung-Ming ChenMorten Arendt RasmussenChung-Sung LeeMorten GrauslundChung-Yuan HsuMorteza Hasanzadeh KafshgariChun-Han ChenMoshe ReuveniChun-Hsin ChenMoshi GesoChun-Jung ChenMostafa A. HussienChunlei LiMostafa Kamal SarkerChunliu ZhuoMotaher HossainChunming LiuMotilal MatheshChun-Peng LiaoMotohiko KondoChunye ZhangMotohiro TaniChunyi WuMotoki KanekatsuChunying LiMotoki TominagaChunzhang YangMotoki UedaCiara M. MurphyMotomu ShimaokaCicero ArrigoMotonari KondoCiesla MaciejMotonori OtaCindy BarnigMoumita DattaCinja KappelMounir MensiCinzia AntognelliMounira ChakiCinzia AuritiMourad Ben SaidCinzia BerteaMoussa Antoine ChalahCinzia FabriziMozaniel OliveiraCinzia FiondaMuck-Seler DoroteaCinzia FranchinMudasir Bashir GugjooCinzia GiacomettiMudit MishraCinzia GiagulliMuhammad Ahsan SaeedCinzia La RoccaMuhammad Amjad NawazCinzia MasperoMuhammad Ayaz AnwarCinzia PaganoMuhammad NomanCinzia ParoliniMuhammad NumanCinzia VolontéMuhammad QasimCiprian RezusMuhammad Saad KhanCiprian TomuleasaMuhammad Sohail ZafarCirino BottaMuhammad Umar KhanCiro De LucaMuhammad Usman GhoriCiro IaccarinoMuhammad Wajid UllahCiro Leonardo PierriMuhammed AtamanalpClaire A. DessallesMukesh Kumar SriwastvaClaire AcquavivaMukhtar AhmedClaire CarterMunari FabioClaire E. Kendal-WrightMunekazu KomadaClaire H. StevensMuneyoshi OkadaClaire HillsMunitta MuthanaClaire LugnierMura Jyostna DeviClaire RoddieMurakami TatsuyaClaire Rodriguez-LafrasseMurtaza TambuwalaClaire SmithMurugesan ChandrasekaranClaire Stines-ChaumeilMustapha Cherkaoui-MalkiClaire WhyteMuthia ElmaClara BaldinMuthu ThiruvengadamClara De PalmaMuthuramalingam PandiyanClara E. UrzìMuzamil Yaqub WantClara IannuzziMyeong-Cheoul ChoClara MeanaMyeong-Hyeon WangClara TangMyeounghoon ChaClarissa A. HenryMykhaylo PotopnykClarissa BracciaMyong-Hee SungClarissa Fantin CavarsanMyoung-Chong SongClarissa P. FrizzoMyoung-Hwan ParkClarisse BrígidoMyra VillarealClaude TaillefumierMyriam BrugnaClaudia AltomareMyron R. SzewczukClaudia CavaMyung-Bok WieClaudia CicioneN/A AbdullahClaudia ContiniNada LabajovaClaudia CristianoNadal-Sala DanielClaudia CrociniNadezhda GolubkinaClaudia CurciNaga Vara Kishore PillarsettyClaudia DellaviaNagendra Kumar KaushikClaudia DittfeldNaji KharoufClaudia DurantiNam V. HoangClaudia FerroniNamkwon KimClaudia GhignaNancy SpanòClaudia Herrera-de GuiseNândor ÉberClaudia KitzmüllerNaoki AsanoClaudia KowolikNaoki KataseClaudia Luevano-ContrerasNaoki MoritaClaudia MaierNaoki TakizawaClaudia Maria RaduNaoki TanimizuClaudia MartiniNaoki WatanabeCláudia MirandaNaotake KonnoClaudia MorgantiNaoto KawakamiClaudia PeitzschNaoyuki KataokaClaudia PennaNaoyuki KawaoCláudia PereiraNaozumi IshimaruClaudia PisanuNapione LuciaClaudia Rangel-BarajasNarayanaganesh BalasubramanianClaudia RiccardiNarcisa VrinceanuClaudia SchneiderNariman BattulinClaudia StrafellaNariyoshi ShinomiyaClaudia Uhde-StoneNatalia FilippovaCláudia VicenteNatalia LinkovaClaudine S. BonderNatalia MiękusClaudine SeeligerNatalia RakislovaClaudio BernardazziNatalia SizochenkoClaudio BonghiNatalia StozhkoClaudio BucoloNatalia V. VasilyevaClaudio CostantiniNataliya A. BabenkoClaudio FoschiNataliya KolosovaClaudio GambardellaNataliya SaninaClaudio GrassiNatalya PavlovaClaudio LuchiniNatarajan RanganathanCláudio MaiaNathan JohnsonClaudio MarascaNathan TannerClaudio O. GualerziNatsuko MiuraClaudio OrtolaniNawab DarCláudio RoqueNectarios VidakisClaudio RossiNediljko BudisaClaudio ScuoppoNehal ElsherbinyClaudio TaloraNessy JohnClaudio ZuccaNéstor Ventura-AbreuClaudiu N. LunguNguyen Minh DucClelia MadedduNguyen Quoc Khanh LeClémence CheignonNiamh CawleyClémence RichettaNicholas Agung KurniawanClemens HinterleitnerNicholas B. LarsonClemens K. WeissNickolai A. BarlevClemens PeterbauerNicola Biagio MercuriClemens Von SchackyNicola OrigliaClemens ZwergelNicolai BovinClément CarréNicolas TremblayClementina ManeraNicole L. BatenburgCleo GoyvaertsNicole SchuppClévio NóbregaNidhi Jalan-SakrikarClizia VillanoNiels HansenClotilde LauroNika LovšinClotilde PolicarNikhil AwatadeClovis ChabertNikki ReinemannCoco SilviaNikolaos AntonakopoulosCodruta SoicaNikolaos DiakakisCodruta VarodiNikolay A. KolchanovCoeli M. B. LopesNikolay A. PyataevCoen C. PaulusmaNima AbbasianColin A. B. JahodaNing LiColin BonduelleNing QuColin C. SeatonNiraj LodhiColin CampbellNisha SinghColin GatesNishikawa Shuh IchiColin P. JohnsonNitin KumarColin W. MacDiarmidNobutaka SomeyaColm O′RourkeNobutake OzekiColm RyanNobuyuki KimuraCon SullivanNobuyuki MaruyamaConcepció MarinNorbert LihiConcepción Gómez-MenaNorbert WikonkálConcetta AltamuraNorifumi ShiodaConcetta FedericoNorihiro InagakiConcetta ScimoneNoriko TakegaharaConcettina La MottaNorio SuzukiCongBao KangNoriyoshi UsuiCongrui JinNuc PrzemysławCongshan SunNuno SantaremCongyue PengO. V. PershinaConor M. HaneyOadi MatnyConrad NieduszynskiObul Reddy BandapalliConsolato SergiOfer BinahConstantin ApetreiOfer PragerConstantin CaruntuOfir WolachConstantina AggeliOka AkihikoConstantino MartínezOksana SherstnevaConstantinos StathopoulosOksana SintsovaConstantinos TsitsilianisOksana V. SalomatinaConsuelo AmantiniOle PetersenConsuelo SerresOleg BelovCoralie Di ScalaOleg SerovCord M. BrundageOleg TimofeevCordelia BolleOlena GoncharukCorina Marilena CristacheOlesya V. StepanenkoCorina NagyOlga A. AleynovaCorina PienarOlga A. GlazunovaCorina S. PopOlga A. SnytnikovaCorinna GrasemannOlga E. AndreevaCorinna SeligerOlga J. BakerCorinne LionneOlga LuzinaCorinne MercierOlga MorozovaCorinne Pau-RoblotOlga OstrovskyCorinne PrévostelOlga PagonopoulouCormac MurphyOlga RedinaCornelia BraicuOlga S. FedorovaCornelia KoyOlga ShamovaCornelia MirceaOlga TarasovaCornelis Johannes Forrindinis Van NoordenOlga TsaveCorneliu TanaseOlga TuraCornelius KraselOline K. RonnekleivCorrado RomanoOliver OttmannCorrado TagliatiOliver SanderCorreia Joana SofiaOlivera TopalovicCostantino PaciollaOlivier HuckCostanza Maria CristianiOlivier PierreeicheCostas BatargiasOlivier VallonCostas C. PapagiannitsisOlivier Van WuytswinkelCostas DelisOluwatosin OluwadareCostea TeodoraOm Parkash DhankherCotas JoãoOmar F. KhanCourtney McDonaldOmar TlibaCovadonga HuidobroOmar ValssonCraig BlackstoneOmaththage PereraCraig G. BurkhartOmkar Vijay ByadgiCraig J. MedforthOndrej BernatikCrescenzio Francesco MinerviniOndrej CehlárCrescenzio SciosciaOndrej HolasCriscuolo ElenaOndrej KodetCristi L. GalindoOndrej MachonCristian A. Lasagna-ReevesOndřej PlíhalCristian A. Moreno GarcíaOndřej ŠedaCristian BonviciniOndřej SochaCristian MunteanuOndrej VasicekCristian Pablo PennisiOndrej ZitkaCristian ScheauOnofrio LaselvaCristian StătescuOpeyemi LawalCristian V. A. MunteanuOphélia Le ThucCristiano BombardiOrfeo SbaizeroCristiano SoaresOriana Lo ReCristian-Radu JecanOriana TrubianiCristina AntinozziOriol RangelCristina ArrigoniOrit UzielCristina AvontoOrlando CastellanoCristina BarbagalloOrlando Torres-FernándezCristina BenattiOrly Lacham-KaplanCristina CapatinaOrnella CalderiniCristina CarresiOrnella MaglioCristina CarucciOrso FrancescaCristina CarvalhoOrsola Di MartinoCristina CasalouOrsolya BiroCristina ChircovOrsolya BorsaiCristina ClementOrsolya DobayCristina CozziOrsolya Eszter MolnárCristina CrosattiOsamu KumanoCristina DaiaOsamu YoshinoCristina E. MolinaOsborne AlmeidaCristina EguizabalOscar CampuzanoCristina FasolatoÓscar Cordero-LlanaCristina FerrerasOscar LeeCristina Guardia-LaguartaÓscar LópezCristina M. RamírezÓscar M. Bautista-AguileraCristina MaccalliniOscar Malvar VidalCristina MallorOscar NunezCristina ManferdiniÓscar PozoCristina Mas-BarguesOscar WiklanderCristina Mihaela GhiciucOskar SachenkovCristina MinnelliOskar ZakiyanovCristina Montiel-DuarteOsman El-MaarriCristina NadaluttiOstaizka AizpuruaCristina NocellaOsvaldo LanzalungaCristina P. R. XavierOta FuchsCristina P. VicenteOto MelterCristina PapayannidisOurania KostopoulouCristina PeñaOvidio BussolatiCristina PopOvidiu MituCristina PotrichOvidiu TitaCristina PuyOxana KlementievaCristina RomeiÖzlem Güzeloǧlu KayişliCristina Rosell-ValleOzohanics OliverCristina SatrianoOzren JovićCristina ScielzoP. Hemachandra ReddyCristina SegoviaP. W. Physick-SheardCristina SobacchiPabitra Kumar SahooCristina Tomas-ZapicoPablo BascuñanaCristina VassallePablo BlancoCristina VicenzettoPablo G. JambrinaCristina-Maria TrandafirescuPablo Garcia De FrutosCristina-Mihaela LăcătușuPablo Hernansanz-AgustínCristobal MirandaPablo Loza-AlvarezCristoforo ComiPablo Martín-VasalloCristoforo SilvestriPablo Ramos-GarciaČrtomir PodlipnikPachiappan ArjunanCrystal R. ArcherPadma MurthiCrystal S. ShinPadmanabhan PattabiramanCrystal SweetmanPádraig D′arcyCrystel BonnetPadraig StrappeCsaba ÉvaPäivi PeltomäkiCsaba HegedűsPakieli H. KaufusiCsaba HetényiPalani ElumalaiCsilla KallayPalanisamy RavichandiranCsilla TothovaPalanivel KandasamyCsongor KissPalma ScolieriCurtis FrenchPalmieri LuigiCynthia BamdadPalmieri MichelleCynthia DamerPaloma LitonCynthia GleasonPaloma PérezCynthia HaseltinePaloma Tejera NevadoCynthia Marie-ClairePaloma TruebaCynthia RosenthalPamela LeinCyrielle BillonPamela RossoCyril MessaraaPamela TozzoCyril SobolewskiPan Dong RyuCyrille De JoussineauPan Kuan-TingCyrille GrandjeanPan LiaoCyrus MotamedPanagiota XaplanteriCzang-Ho LeePanagiotis J. VlachostergiosD. S. VerbeekPanagiotis KarakaidosDae Ho LeePanagiotis KarrasDae Youn HwangPanagiotis KatinakisDae-Hee LeePanagiotis LiakosDaesung ShinPanagiotis MallisDafne BongiornoPanagiotis MistriotisDafne Campigli Di GiammartinoPanagiotis PapoutsoglouDag Henrik ReikvamPanagiotis PeitsidisDagmar JanovskaPanagiotis SimitzisDagmar Meyer Zu HeringdorfPanagiotis TheodorakisDagmar RiemannPanagiotis TsapogasDagmar StoiberPanagiotis TsiakanikasDagmara BaczyńskaPanagiotis TsikourasDagmara Szmajda-KrygierPanayiota L. PapasavvaDagmara WiniarczykPanayiotis A. TheodoropoulosDaichi MorimotoPanayiotis KouisDaiga KishimotoPandurangan RamarajDaigaku HasegawaPanisadee AvirutnanDaiji EndohPanita MaturavongsaditDaiji KawanamiPankaj AhluwaliaDaijiro YanagisawaPankaj AttriDainis RungisPankaj PandeyDaishi YamakawaPanos G. KalatzisDaisuke FujitaPanyue ZhangDaisuke InouePaola AngeliniDaisuke KodamaPaola BagnoliDaisuke KomuraPaola BellostaDaisuke MikiPaola CappelloDaisuke MiyoshiPaola CartaDaisuke NakanoPaola CorsettoDaisuke SatoPaola CostelliDaitoku SakamuroPaola De LucaDaiva TauraitePaola Di BonitoDakeun LeePaola Di PietroDalal Hammoudi HalatPaola FabbriDali WangPaola FaraoniDalia AlansaryPaola FossaDalia M. KopustinskienePaola GavazzoDalia MedhatPaola GiordanoDalia ZaliunienePaola GiussaniDa-Liang OuPaola GrimaldiDalila MangoPaola LenziDalius ButkauskasPaola LondeiDalton DietrichPaola ManiniDaman KumariPaola Maura TricaricoDamian BartuziPaola MaycotteDamian C. GenetosPaola NavarreteDamian Jozef FlisPaola PalumboDamian PociechaPaola PaoliDamiana LeoPaola ParenteDamiana ScuteriPaola PatrignaniDamiano D′ArdesPaola PierobonDamiano FantiniPaola PiomboniDamien ArnoultPaola QuarantaDamien F. MeyerPaola RivaDamijan KnezPaola RognoniDamu TangPaola RotaDan LuoPaola SarchielliDan SongPaola SavoiaDan TchernovPaola SemeraroDan VoiculescuPaola SimeoneDan ZhaoPaola TogniniDana CucuPaola UngaroDana DlouháPaola VendittiDana Gabriela FestilaPaola ZigrinoDana JurkovicovaPaolina CroccoDana Lucia StanculeanuPaolo AbondioDana Maria CopoloviciPaolo ArosioDana NiculaePaolo BoffanoDanail PavlovPaolo CameliDanay CibrianPaolo CapparèDancho DanalevPaolo CascioDan-Dan ZhangPaolo CavorettoDandan ZhuPaolo CeppiDane WolfPaolo ClavenzaniDania MoviaPaolo D. PigattoDaniah TrabzuniPaolo DolzaniDanial RoshandelPaolo DorettoDaniel A. BailkovPaolo FagoneDaniel AbankwaPaolo GabrielliDaniel Alvarez-FischerPaolo GiuffridaDaniel B. StovallPaolo GrumatiDaniel BäckerPaolo GualtieriDaniel BourquainPaolo GuglielmiDaniel BouyerPaolo LimoliDaniel Carneiro MoreiraPaolo MagniDaniel ColladoPaolo MarianiDaniel DobslawPaolo MeneguzzoDaniel DuerschmiedPaolo MignattiDaniel EllederPaolo NataleDaniel G. BichetPaolo OnoriDaniel GackowskiPaolo PelosiDaniel García SoutoPaolo RagoneseDaniel GibbsPaolo RiccioDaniel GrinbergPaolo RuzzaDaniel HilgerPaolo SantambrogioDaniel J. ShiwarskiPaolo ScudieriDaniel JancuraPaolo SilacciDaniel John VerdonPaolo TondiDaniel JohnsonPaolo TrucilloDaniel KronenbergPaolo TucciDaniel KytyrPaolo ZambonelliDaniel L. VoisinPapale MariaDaniel LarkParagi GáborDaniel LiedtkeParameswaran G. SreekumarDaniel LópezParameswaran Grace LalitkumarDaniel Lopez-MaloParameswari NamasivayamDaniel MachinParamita BasuDaniel MartinParamita ChakrabortyDaniel MatulićParamjit TappiaDaniel MeltersParas Singh MinhasDaniel MenendezParaskevi HeldinDaniel MertensParaskevi KarousiDaniel MorseParesh PrajapatiDaniel NeureiterParés-Matos ElsieDaniel OtzenParimal MisraDaniel P. PotaczekParimal SamirDaniel P. UraPark Hyun-jungDaniel Penteado Martins DiasPark SolipDaniel PletzerParra Maria TeresaDaniel R. BarredaParskson Lee Gau ChongDaniel Ricardo DelgadoParthasarathy A. MadurantakamDaniel SascaParthiban MarimuthuDaniel SchindlerPascal ArnouxDaniel SmržPascal ColosettiDaniel Sobrido-CameánPascal DarbonDaniel StraumePascal GamasDaniel SzulczykPascal H. FriesDaniel TaillandierPascal IckrathDaniel Torres-LagaresPascal LaforêtDaniel VaimanPascal RatetDaniel W. NixonPascal RowartDaniel WefersPascal TouzetDaniel WolffPascal VaudinDaniel YorkPaschalis TheotokisDaniela ArosioPaschalis-Thomas DouliasDaniela BraconiPascual García-PérezDaniela C. VazPasi KankaanpääDaniela CaccamoPaško KonjevodaDaniela CalinaPasquale CrupiDaniela CassolPasquale EspositoDaniela DamianiPasquale MarrazzoDaniela De ToteroPasquale PiconeDaniela De VitaPasquale ScarciaDaniela DolcePasquale SimeoneDaniela DragomanPasqualina D′UrsiDaniela ElettoPasupathi SundaramoorthyDaniela FioccoPatil ShankargoudaDaniela FreitasPatric DelhantyDaniela GabbiaPatrice CoteDaniela GiulianiPatrice GaurivaudDaniela GnaniPatrice N. MarcheDaniela IannazzoPatrice NaudDaniela ImpellizzeriPatrice X. PetitDaniela IsolaPatricia Agudelo-RomeroDaniela Janina BakulaPatrícia AlvesDaniela JežováPatricia BogdanovDaniela KalafatovicPatricia De FranciscoDaniela MarascoPatricia Diaz-RodriguezDaniela MarazzitiPatricia GiraldoDaniela MelnikPatricia HeynDaniela MennerichPatricia J. AllenDaniela MiricescuPatricia J. LardoneDaniela MokraPatricia J. McLaughlinDaniela MontesarchioPatricia Jumbo-LucioniDaniela PedicinoPatricia K. CoyleDaniela PerronePatricia L. Lakin-ThomasDaniela PintoPatricia LalorDaniela PredoiPatricia LeightonDaniela QuaglinoPatricia MartinDaniela RegazzoPatricia Ondine LucaciuDaniela RovitoPatricia PriceDaniela RussoPatricia Seoane-CollazoDaniela SarnataroPatricia Van Oosten-HawleDaniela TalhadaPatricio Fernández-SilvaDaniela ValensinPatricio Iturriaga-VasquezDaniela ValentiPatricio Orellana-PalmaDaniela Valeria MinieroPatrick BradshawDaniele ArmocidaPatrick C. BaerDaniele BellaviaPatrick CelieDaniele BosoPatrick DutartreDaniele CastigliaPatrick F. DowdDaniele D′alonzoPatrick GalloisDaniele La RussaPatrick HannaertDaniele LinardiPatrick HerrDaniele MantionePatrick J. BednarskiDaniele MasaronePatrick JudeinsteinDaniele NosiPatrick KüryDaniele RinaldiPatrick LajoieDaniele VergaraPatrick NarbonneDanielle E. DyePatrick ProvostDanielle H. W. VleckenPatrick VideauDanielle KamatoPatrick Vourc′hDaniel-Valentin SavatinPatrick WeydtDaniil N. BratashovPatrina ParaskevopoulouDaniil NikitinPatrizia BottoniDanijel SikicPatrizia BovolinDanilo CimadomoPatrizia CancemiDanilo MilardiPatrizia Dell′EraDanilo Roman-CamposPatrizia FalabellaDanka GrčevićPatrizia GaspariniDanko BakarčićPatrizia GenaDanko MiloševićPatrizia GiannoniDanlong JingPatrizia LeoneDanmeng LuoPatrizia Nadia HaniehDanni HarrisPatrizia PignattiDanon Clemes CardosoPatrizia Polverino De LauretoDanuta JantasPatrizia ProiaDanuta Januszkiewicz-LewandowskaPatrizia ZaramellaDanuta Lietz-KijakPatrycja KleczkowskaDanuta PentakPatrycja MakośDanuta WitkowskaPatrycja WińskaDanuta WojcikPatryk LipinskiDaoquan XiangPaul A. SievingDaphne AutranPaul ComanDaphné Dupéré-RicherPaul D. BoudreauDarci TraderPaul DalhaimerDaria AugustyniakPaul F. WilliamsDaria BortolottiPaul Francis MorrisDaria NurzynskaPaul G. FurtmüllerDaria PoshinaPaul GagniucDaria SicariPaul GooleyDaria SkuratovskaiaPaul GouguetDaria V. VasinaPaul HeathDarija CörPaul J. LucassenDar-In TaiPaul KosmaDarin ZertiPaul LucassenDarío Acuña-CastroviejoPaul MozdziakDario BalestraPaul MurrayDario BonettaPaul PirteaDario BruzzesePaul ProostDario Domenico LofrumentoPaul QuaxDario PaoloPaul ReynoldsDario RuscianoPaul RiskaDario SiniscalcoPaul RosenbergDarius KalasauskasPaul SchoenhagenDariusz BoguszewskiPaul SchofieldDariusz BorońPaul SquiresDariusz DzikiPaul Takam KamgaDariusz GrzebelusPaul TownsendDariusz JedrejekPaul VasosDariusz KokoszyńskiPaul VeithDariusz KucharczykPaula A. OliveiraDariusz KulusPaula Alexandra A. B. LopesDariusz M. LebensztejnPaula Daza-NavarroDariusz MlynarczykPaula HoffDariusz PańkaPaula I. MoreiraDariusz PawlakPaula LudovicoDariusz PiesikPaula M. FerreiraDariusz RakusPaula MeleadyDariusz SzczepanikPaula MuloDariusz T. MlynarczykPaula PauloDarja LavoginaPaula PeixeDarko BožićPaula PereiraDarko PreinerPaula V. MonjeDarlene DarttPaula VerissimoDarryl B. HoodPaulina CzaplewskaDarya NovopashinaPaulina GlazińskaDaumantas MatulisPaulina KasperkiewiczDau-Ming NiuPaulina KazimierczakDave SpeijerPaulina Kosikowska-AdamusDavid A. BenfieldPaulina KrawiecDavid A. BereiterPaulina MajewskaDavid A. GellPaulina Niedźwiedzka-RystwejDavid A. JacobsonPaulina PecynaDavid ActonPaulina TokarzDavid B. AlexanderPaulina ŻelechowskaDavid BalayssacPauline ColombierDavid BerstenPaulino Gómez-PuertasDavid BrindleyPaulo BettencourtDavid BurnsPaulo CoelhoDavid C. IrwinPaulo Correia-de-SáDavid C. StuckeyPaulo J. PalmaDavid ChakravortyPaulo KofujiDavid Changli WeiPaulo MatosDavid ClosePaulo N. MatafomeDavid ColecchiaPaulo SantosDavid ConversiPauloke ShowDavid CoquerelPaulus RommerDavid CouvinPavana RottiDavid CuberoPavel A. ZykinDavid DaudéPavel BashtrykovDavid DayPavel DráberDavid DixonPavel FeduraevDavid DolivoPavel HasalDavid E. VancePavel IzakDavid EcclesPavel LobachevskyDavid EscorsPavel MalyDavid FangPavel MelnikovDavid Fernandez-RemolarPavel PadnyaDavid G. MiguezPavel PashkovskiyDavid GallPavel RozsivalDavid GraingerPavel SpirinDavid GreenhalghPavel ŠtarhaDavid H. SherrPavel StopkaDavid HarmanPavel V. AvdoninDavid HervasPavel VítámvásDavid HerzogPavla JendelovaDavid HicksPavla Manaskova-PostlerovaDavid HillPavlina DolashkaDavid HokszaPavol ZelinaDavid HughesPawan Kumar MishraDavid J. ClarkPawel Antoni KolodziejskiDavid J. McgeePawel BieganowskiDavid Jesse SanchezPaweł BrzuzanDavid KennedyPawel DabrowskiDavid KomatsuPaweł GacDavid KonkinPaweł GrzmilDavid L. CarawayPawel KafarskiDavid LambPaweł KordowitzkiDavid LeavesleyPaweł KowalczykDavid LibichPawel KozielewiczDavid LutzPawel LipinskiDavid M. BrownPawel MakDavid MaciasPawel NakielskiDavid MagnePaweł NawrotekDavid MagnusonPaweł OlczykDavid MandelbaumPaweł P. PomastowskiDavid Martín-HidalgoPaweł PietkiewiczDavid MedinaPaweł PiszkoDavid Medina-CruzPaweł PomorskiDavid MengelPawel RubisDavid MillsPaweł SiudemDavid MonchaudPaweł ZalewskiDavid MurrayPawel ZmoraDavid OrnellesPayam EmamiDavid PadrosaPayam SadeghiDavid PoppersPediaditakis IosifDavid R. BeersPedro A. Jiménez GómezDavid R. CalabresePedro AciénDavid RamsdenPedro BrugarolasDavid RoquisPedro CasadoDavid S. P. CardosoPedro Cosme RedondoDavid S. SalomonPedro De Barros FernandesDavid SandquistPedro De-la-TorreDavid SanterPedro EscollDavid ŠilhaPedro FerreiraDavid SloukaPedro Gonzalez-MenendezDavid SmithPedro HornaDavid SteffenPedro Humberto CastroDavid T. StuartPedro M. DomingosDavid Van IJzendoornPedro Martínez-GómezDavid Varillas DelgadoPedro MartinsDavid VaudryPedro Miguel RodriguesDavid VirshupPedro MorouçoDavid WelchPedro NietoDavide BarbagalloPedro Ojeda-MayDavide BarrecaPedro PedrosaDavide BorroniPedro PiedrasDavide CavagnettoPedro Rebelo-GuiomarDavide CoràPedro ReisDavide CossuPedro RoblesDavide CristoforiPedro TalhinhasDavide FerrigoPeep PalumaaDavide Giuseppe RibaldonePeeyush RanjanDavide GuerraPeggy StockDavide LauroPei Feng LiDavide MedicaPei-Cheng HuangDavide MugettiPei-Chun LiaoDavide RissoPei-Chun WongDavide TagliazucchiPeiguo YuanDavina Camargo Madeira SimoesPei-Li YaoDavood KharaghaniPei-Ling HsiehDavor MargetićPei-Luen LuDawid MikulskiPei-Ming ChuDawid PrzystupskiPeipei WangDawid SzczepankiewiczPei-Ra LingDawon KangPei-Shi YenDax Aaron HoffmanPei-Wen ChenDe La Fuente Alerie GuzmanPellegrino MazzoneDea SladePenélope AguileraDean KavanaghPenelope Kay-FedorovDean KonjevicPeng LiDeanna Funnell-HarrisPeng LiaoDebabrata BanerjeePeng WangDebarati BasuPengcheng WangDebasish BandyopadhyayPer Jr GreisenDebasish BasakPerco PaulDebasish BoralPere PuigdomènechDebasish Kumar DeyPericacho MiguelDebbie C. CransPeriyasamy SivalingamDebbie C. ThurmondPerla PucciDebmalya BarhPernille SarupDebolina BiswasPer-Olof SyrénDebopam ChakrabartiPerrine BortolottiDebopam GhoshPetar LambrevDebora CarpanesePetar OzretićDébora FerreiraPeter A. KeyelDebora I. SinnerPéter Ábrányi-BaloghDebora LanznasterPéter ÁcsDebora Lo FurnoPeter AngeleDebora NapoliPeter B. GahanDeborah A. SarkesPeter BaasDéborah ChicharroPeter BališDeborah CroteauPeter BrizaDeborah GalpertPeter BrossDeborah P. FischerPeter ChibaDeborah QuaglioPeter Christopher KonturekDeborah RudinPeter David ZalewskiDeborah StancoPeter De CosterDebottam SinhaPeter EngelmannDeclan DevinePeter GronesDeclan P. McKernanPeter HauDeepak ChandrasekharanPeter HauserDeepak DeshpandePeter HeisigDeepak KumarPeter HemmerichDeepak SinghPeter HerseyDeepti ChaturvediPeter HlivakDeGaulle I. ChigbuPeter IllesDegeng WangPeter Ivanov HristovDeivendran RengarajPeter J. ChoiDejan BudimirovicPeter J. EggenhuizenDejan JakimovskiPeter J. HolstDejan StojkovićPeter J. SiskaDejan VidovicPeter JarcuskaDejana PankovicPeter John JervisDe-Li ShiPeter JonesDelia HorhatPeter KaplanDelia Mihaela RaţăPeter KoulenDelia Mirela TitPeter KrenekDelia PiconePeter KubatkaDelisha StewartPeter KubiszDelphine BohlPeter L. A. M. VosDelphine LarrieuPeter LindemannDelphine MaurelPeter LuntDelphine QuénetPeter M. AndersonDémène HélènePeter MantleDemeter TzeliPeter MikušDemetra S. M. ChatzileontiadouPeter MonkDemetrios ArvanitisPeter N. SteinmetzDenes TothPeter OelschlaegerDeng-Ho YangPeter P. LinDenis B. ZolotukhinPeter Palove BalangDenis C. BauerPeter ParsonsDenis D. de KeukeleirePeter PensonDenis GaroliPeter PolčicDenis KorneevPéter PoórDenis LafontainePeter R. LaityDenis MottetPeter R. MertensDenis MurphyPeter RiedererDenis NazarovPeter RumneyDenis PolancecPeter ScharffDenis RychkovPeter SchemmerDenis S. KudryavtsevPeter SchlenkeDenis SerenoPeter SetlowDenis SilachevPeter SmithamDenisa MarginaPeter SolárDenise BellisarioPéter SzentesiDenise CorneliusPéter SzolgayDenise HaroldPeter T. RuaneDenise SchramaPeter ThompsonDenise ToscaniPeter TimmermanDennis BrownPeter TomčíkDennis GerloffPeter TothDennis GreerPeter UppstuDennis HwangPeter Van DamDennis J. WormPeter Van VeldhuizenDennis MetzgerPeter WehlingDenys A. KhaperskyyPeter ZhelevDeo SinghPēteris TretjakovsDerek McMahonPetr ChlapekDerek NarendraPetr HodekDerek S. BoeldtPetr HumpolíčekDerek T. A. LamportPetr KomínekDerin Benerci KeskinPetr MalýDerry K. MercerPetr MlejnekDerval Dos Santos RosaPetr NachtigalDer-Yen LeePetr O. IlyinskiiDer-Yuan ChenPetr SergievDesai SanjayPetr ŠimekDésirée TampePetr SmykalDespina SamakovliPetr TomekDessislava TodorovaPetr TvrdikDevadas KrishnakumarPetra DunkelDevasena PonnalaguPetra HrubáDeven PatelPetra I. LorenzoDevis BenfaremoPetra ImhofDevoy AnnyPetra Ina PfefferleDeyang XuPetra KoraćDhamodharan VenugopalPetra KosutovaDhananjay YadavPetra LiskovaDhananjaya KulkarniPetra LutterDhanasekaran VikramanPetra M. KlingeDhanendra TomarPetra M. WiseDhanush HaspulaPetra MarhavaDharmendra ChoudharyPetra MrozkovaDharmendra K. YadavPetra PallagiDharmendra Pratap SinghPetra Peharec ŠtefanićDhifaf SarhanPetre IonitaDhivya SudhanPetrice M. CogswellDhriti SinhaPetro Onys′koDhruvajyoti RoyPetronila PenelaDi LiuPetros ChristopoulosDi Miceli MathieuPetros KolovosDi RenPetros P. PetrouDi ShiPetros PapadopoulosDiaga DioufPetros PatsaliDiana AntalPetros T. DamosDiana Camelia NuţǎPetrov VesselinDiana CiolacuPham Thu-HuyenDiana Cláudia PintoPhaneendra K. DuddempudiDiana Constantinescu-AruxandeiPhil. WhitfieldDiana Cunha-ReisPhilip Brandon BusbeeDiana DonatellaPhilip C. TrackmanDiana GuallarPhilip CalderDiana KolbePhilip HublitzDiana MechtcheriakovaPhilip KitchenDiana MieliauskaitėPhilip Marx-StoeltingDiana PintoPhilip OwensDiana PortanPhilip ProcterDiana RafaelPhilip SerwerDiana RamasauskaitePhilip TedburyDiana SalikhovaPhilip W. WertzDiana SavuPhilipp ArnoldDiana TilevikPhilipp ErbenDiandra De AndradesPhilipp KolbDianne WaltersPhilipp OrekhovDidac SantesmassesPhilipp S. LangeDidier DevaursPhilipp StarklDidier F. PisaniPhilippe A. RobertDidier GonzePhilippe AndreyDiego AlbaniPhilippe BuletDiego Angosto BazarraPhilippe De DeurwaerdereDiego CotellaPhilippe De MoerlooseDiego De MiguelPhilippe DelannoyDiego De Miguel-PérezPhilippe DumasDiego Dos Santos FerreiraPhilippe EtienneDiego F. CalvisiPhilippe GasqueDiego F. TiradoPhilippe GeorgelDiego Franco JaimePhilippe GossetDiego García-GonzálezPhilippe GuerreDiego GuidolinPhilippe GuilpainDiego La MendolaPhilippe JeandetDiego ManavellaPhilippe LabelDiego QuirogaPhilippe LabruneDiego RaimondoPhilippe LecomteDiego Romano PerinelliPhilippe LesnikDieter FinkPhilippe LewalleDieter SchmollPhilippe M. OgerDietmar AbrahamPhilippe MarmottantDietmar EnkoPhilippe NgheDietmar KrautwurstPhilippe UrbanDietmar ZaissPhotini MylonaDijana ŽilićPhuong T. N. HoangDiletta AmiPhu-Tri TranDilfuza EgamberdievaPia GiovannelliDilip ChallaPia HartmannDimitar A. DobchevPickrell AliciaDimitra MichaPier Camillo ParodiDimitri PestovPier Carlo RicciDimitri PoddighePier Francesco FerrucciDimitrios FanourakisPier Luigi StefànoDimitrios GiliopoulosPier Nicola SergiDimitrios KaragiannakisPiera Anna MartinoDimitrios KazisPierantonio De LucaDimitrios KiortsisPierfrancesco FrancoDimitrios KlaoudatosPiergiorgio La RosaDimitrios PanagopoulosPierluigi CaboniDimitrios PapadimitriouPierluigi PlastinaDimitrios StakosPiero BaroneDimitrios StellasPiero RicchiutoDimitrios TsaopoulosPierpaolo CeciDimitrios TzeranisPierre Alexandre KaminskiDimitrios ZeugolisPierre BarretDimitris EmfietzoglouPierre BongrandDimitris KardassisPierre CornelisDimitris L. BouranisPierre Damien CoureuxDimitris TsikasPierre De MeytsDimitry Arkadievich AfonnikovPierre DuquetteDimitry SchepetovPierre JacobDimitry VasilyevPierre KochDimosthenis KizisPierre LapaquetteDina AbusamraPierre RougéDina K. GaynullinaPierre VincentDina YarullinaPierre-Antoine BonnetDinender SinglaPierre-Aurélien BeuriatDinesh Chandrasekaran DhurvasPierre-Olivier AngrandDinesh Kumar VermaPiersandro PallaviciniDineshkumar SengottuveluPieter-Jan D. GunsDing WangPiétrick HudhommeDinko NovoselPietro AmeriDino LuethiPietro CacialliDinora F. González EsquivelPietro CampigliaDiogo FonsecaPietro MesircaDionysia PapagiannopoulouPietro NapoliDionysios ChartoumpekisPietro RoversiDionysios WatsonPil Soo SungDipa PatiPilar HoyosDipak BanerjeePilar López-CornejoDipak KathayatPinar Uysal-OnganerDipankar RoyPinelopi ArvanitiDiptiman BosePinelopi I. ArtemakiDirk De RooijPing K. YipDirk DobritzschPing SongDirk HenrichPing ZhouDirk MontagPing-Chieh PaoDirk SchepmannPing-Chih HsuDirk SpitzerPing-Hsing TsaiDirk TischlerPing-Hui TsengDisha SharmaPingping HanDishary BanerjeePingyuan WangDita MaixnerovaPin-jung ChenDivanizia Do Nascimento SouzaPin-Kuang LaiDivya BhagirathPio ContiDivya KalraPiotr BalczewskiDivya MurthyPiotr BełdowskiDivya RamchandaniPiotr BonarekDivya SharmaPiotr BrągoszewskiDjenisa RochaPiotr ChmielarzDmitri PervouchinePiotr DobrowolskiDmitri SobolevPiotr DobrzynskiDmitri V. NikitinPiotr DziegielDmitrii KorzhevskiiPiotr F. J. LipińskiDmitris XirodimasPiotr GolecDmitriy BerilloPiotr Henryk SkarzynskiDmitriy NiyazovPiotr KapustaDmitriy S. BlokhinPiotr KawczakDmitry A. GorbachevPiotr KonopelskiDmitry A. MaslovPiotr KulinowskiDmitry A. SuplatovPiotr MarszalekDmitry EnikeevPiotr MichelDmitry G. KvashninPiotr MuchaDmitry I. OsmakovPiotr OgrodowiczDmitry KarpovPiotr OstaszewskiDmitry KhochenkovPiotr OwczarzDmitry KostyushevPiotr PoznanskiDmitry LoginovPiotr RychahouDmitry PenkovPiotr ŚwiątekDmitry RodionovPiotr SzulcDmitry TelyshevPiotr SzwedaDo Ha Thi ThuPiotr WlaźDobrila NesicPiotr WychowańskiDobrochna Adamek-UrbańskaPiotr ZielenkiewiczDohyun HanPipitsa N. ValsamakiDokyun NaPiter Jabik BosmaDolores Ortiz-MasiáPiyali DasguptaDolores SubiráPiyush BaindaraDomenica Donatella Li PumaPiyush Sindhu SharmaDomenica LoveroPiyusha PagareDomenico Azarnia TehranPiyushkumar MundraDomenico BuonavogliaPlacido BruzzanitiDomenico CassanoPlácido LlanezaDomenico CiavarellaPlacido MineoDomenico DalessandriPlacido NavasDomenico De PaolaPlamena R. AngelovaDomenico IacopettaPo-Chen ChuDomenico LioPo-Ching ChengDomenico MaisanoPo-Han ChenDomenico MajolinoPo-Hsien LiDomenico MattoscioPo-Lin ChiuDomenico PignonePo-Lin LiaoDomenico RaimondoPolina PerelmanDomenico RussoPolina Yu. VolkovaDominic Chih Cheng VoonPoliraju KalluruDominic EvangelistaPolona ZigonDominic WellsPoming ChowDominik DłuskiPons-Tostivint ElvireDominik FuhrmannPontus AspenströmDominik PoradowskiPooja SabhachandaniDominik S. SkibaPothana SaikumarDominik SaulPoulami SarkarDominika Drulis-FajdaszPoushali DasDominika GłąbskaPouya DiniDominika MalińskaPouya JavadianDominique BourgeoisPovilas KavaliauskasDominique DebannePrabaha SikderDominique LerouetPrabhakaran SoundararajanDominique LunterPrabhu PonnandyDominique ModrowskiPrabhu Srinivas YavvariDomitilla MandatoriPrabodh SatyalDomizia VecchioPrachi UmbarkarDon GreenPradeep Kumar BollaDonald AnthonyPradeep Kumar SinghDonald CameronPradeep PaudelDonald E. SprattPradeep VaradwajDonald JacobsPradip DasDonald LucasPraful R. NairDonald RonningPrajish IyerDonat AlparPrakash DoddapattarDonata CafassoPrakash GangadaranDonata FigajPrakash RaiDonatella CarusoPrakash S. BisenDonatella Delle CavePramod SubediDonatella Serafini FracassiniPramod YadavDonatella StilliPranabananda DuttaDonatella VerbanacPranay SrivastavaDonato AngelinoPraneeti PathipatiDonato D′AgostinoPranjal SarmaDonato SantovitoPranshu SahgalDong ChenPrasad TrivediDong HanPrasannavenkatesh DuraiDong Hun LeePrasanta K. SubudhiDong Jun LimPrasanth PuthanveetilDong ZhangPrasenjit SahaDongbao ChenPrashant KaushikDongchul KangPrashanth Kumar Bhusetty NageshDongheon LeePrashanth SuravajhalaDong-Hoon JeongPratheepa RasiahDonghwa KimPratik DhakalDong-Hwee KimPratima LabrooDong-Hyun ShinPraveen AwasthiDongin KimPraveen Kumar KorlaDongjian HuPraveen P. N. RaoDong-Kuk AhnPredrag NovakDong-Kyu KimPreetish MurthyDongmei LiPrem L. BhallaDongsheng JiangPrema Latha MallipeddiDongshi WanPrihardi KaharDongying LiPrimoz RozmanDonika G. IvanovaPriscilla GuglielmoDon-Kyu KimPrisco MarinaDon-Marc FranchiniPritam GangulyDonna GoldhawkPritam SadhukhanDóra K. MenyhárdPritam SinharoyDora KovacsPritha MajumderDorcas OrengoPriya BalasubramanianDoreen LauPriya VoothuluruDoreen SchmidlPriyakshee Borpatra GohainDorianna SandonàPriyamvada JayaprakashDorien ClarissePriyanka KulkarniDorien SchepersPriyanka MishraDoris F. OberlePriyanka PanwarDoris Rosero-GarciaPriyanka RayDoris RusicPriyanka SharmaDoron ShkolnikPriyankar DeyDoron TeperPromita BhattacharjeeDorota BabilasProspero CivitaDorota Bonarska-KujawaPrzemko TylzanowskiDorota ChudobaPrzemysław DudaDorota Gołąbek-SulejczakPrzemyslaw GagatDorota Iwaszkiewicz-GrzesPrzemyslaw J. KotylaDorota KwiatkowskaPrzemysław KopećDorota NieoczymPrzemyslaw MielczarekDorota RybaczekPrzemysław PłocińskiDorota SulejczakPrzemysław TomasikDorota Swiatla-WojcikPui Wah ChoiDorota Tichaczek-GoskaPullagurala Venkata Laxma ReddyDorota Tomaszewska-ZarembaPumtiwitt McCarthyDorota WłogaPuneet AgarwalDorota WrześniokPung Pung HwangDorota Zarębska-MichalukPunit P. SethDorota Zięba-PrzybylskaPuppo FrancescoDorothee KieferPuran PandeyDorothy M. SuppPurushoth EthirajDorothy VittnerPurushothaman NatarajanDouglas C. HooperPurusotam BasnetDouglas C. WeiserPushp Sheel ShuklaDouglas G. WalkerPushpa Raj JoshiDouglas LaurentsPushpamali De SilvaDouglas LeamanPyotr Tyurin-KuzminDouglas P. ThewkePyrear Suhui ZhaoDouglas RootPyung-Hwan KimDouglas Silva DominguesQaisar MahmoodDowning Charles AndrewQaisar MaqboolDragana D. Vasić AnićijevićQamar U. ZamanDragana GabrićQammer ZaibDragana Samardžija NenadovQari Muhammad ImranDragana TrifunovicQasim AlhadidiDrago BešloQazi Mohd Rizwanul HaqDragoljub GajicQi ChenDragomir MilovanovicQian DuDragos Catalin JianuQian QinDragos CretoiuQiang FuDragos SerbanQian-Hao ZhuDragutin SevicQie ShuoDrazen PetrovQifei LiDrozdstoj St. StoyanovQing DengDuaa DakhlallahQing JiangDuane HallQingbin CuiDubravka Švob ŠtracQingbo LiuDuc-Thang VoQinghang MengDulce Maria Palmerin-CarreñoQinghui MuDumitru Constantin-TeodosiuQinghui WangDunja BandeljQingming FangDunja Maria Baston-BüstQing-Rong LiuDunja ŠamecQingxia ZhaoDuo ZhangQingyu ZhouDurdica UgarkovićQinzhe WangDurga ParajuliQiong YangDušan CmarkoQiong ZhangDušan DimićQiuyang ZhangDušan LazárQizhou LianDusan TurkQu DengDušan ŽitňanQuan ZhangDusana MajeraQuanah J. HudsonDushyant MishraQuan-Bin HanDylan Jack RichardsQuanlong LuDzmitry ShcharbinQuentin VicensE. A. SmirnovaQuintino Giorgio D′AlessandrisE. D. GomesQutuba G. KarwiE. H. HoefslootQuynh Nhu DinhE. Mark WilliamsR. DefezE. Sylvester ViziR. KocyłowskiE. V. SchmalhausenR. Martin VabulasEberhard GrambowR. Nicholas LaribeeEberhard HildtR. Rama SureshEberhard KorschingR. Thomas BoydEberhard SchlickerRaafat El-NamakyEdel M. HylandRabban MangatEdgar De AlmeidaRabia AyubEdith BonnelyeRachana D. ShahEdjis VaversRachana TrivediEdmund CarvalhoRachel A. KochEdmund KoziełRachel Bar-ShavitEdoardo BertoliniRachel K. MillerEdoardo MaghinRachel McQuadeEdoardo SalladiniRachel N. LippertEdorta Santos VizcaínoRachel PalinskiEdouard EvangelistiRachel SherrardEduard BardajíRachel ThijssenEduardo Andrés-LeónRachel Wah Shan ChanEduardo Arellano-RodrigoRachel WallerEduardo CiresRachele GarellaEduardo CostaRachele IsticatoEduardo Esteban-ZuberoRachele TamburinoEduardo GuzmánRachid BouharroudEduardo H. Sánchez-MendozaRachid LahlilEduardo LazarowskiRachna ShahEduardo López-UrrutiaRachy AbrahamEduardo Mateo-BonmatíRade M. BabićEduardo OliverRadek IndraEduardo PuellesRadek PohlEduardo SanteroRadek ŠindelkaEdurne Avellanal ZaballaRadha GopalEdurne San JoséRadhakrishnan PanatalaEdvard EhlerRadhia M′kacherEdvard SmithRadhika AmaradhiEdvardas DanilaRadim HavelekEdward A. Ruiz-NarváezRadim VrzalEdward F. ConnorRadka GorejováEdward H. SharmanRadka PodlipnáEdward J. PavlikRadka SymonovaEdward L. BoltRadka VaclavikovaEdward LauRadojica DjokovicEdward Lee-RuffRadoslaw BednarekEdward NarayanRadosław ChaberEdward P. C. LaiRadosław DembczyńskiEdward P. CarterRadoslaw DrozdEdward R. SauterRadosław KempińskiEdward SacherRadosław KujawskiEdward WagnerRadoslaw M. SobotaEdwards A. ParkRadosław RadzkiEdwige VoissetRadosław RolaEdwin D. LephartRadosław ZagożdżonEdyta BiskupRadostina GeorgievaEdyta Boros-LajsznerRadu AlbulescuEdyta BrzóskaRadu Claudiu FierascuEdyta Gendaszewska-DarmachRadu Dorin AndreiEdyta MądryRadu Liviu SumalanEdyta MakuchRadu MineaEdyta Marta BorkowskaRadu Silaghi-DumitrescuEelco De BreeRaelene PickeringEfrat Shavit-SteinRafael A. CasusoEfrosyni ParaskevaRafael BaptistaEfstathia K. KapsogeorgouRafael CoveñasEfstathios D. PagoureliasRafael Garcia TavaresEfstratios StratikosRafael GozalbesEfstratios Stratos NikolaivitsRafael Guillén-BejaranoEfstratios-Stylianos PyrgelisRafael Maria PrietoEfthymios MotakisRafael Prado-GotorEgeria ScodittiRafael Salto-GonzalezEgidio CelentanoRafael SeoaneEglantina IdrizajRafael V. DavalosEgoitz AstigarragaRafal ArchackiEgor A. TuróvskyRafal BaranskiEgor B. ProkhortchoukRafał ButowtEgor PlotnikovRafał FrańskiEhsan FeizollahiRafał LatajkaEhsan KarimiRafal MoszynskiEhud CohenRafal OgorekEhud RazinRafał StarzyńskiEiichi KumamotoRafal ZielinskiEiichi SatoRaffaele CapassoEiichi ShoguchiRaffaele Dello IoioEiichi WatanabeRaffaele FrazziEiichiro NagataRaffaele GalieroEija NissiläRaffaele IevaEiji KawamotoRaffaele NatellaEiji MiyoshiRaffaele NuzziEiji NambaraRaffaele OrnelloEiji YoshiharaRaffaele PillaEijiro JimiRaffaele SerraEikan MishimaRaffaella CascellaEike Benjamin BrauneRaffaella ComitatoEileen BrantleyRaffaella LombardiEirini KitsiouliRaffaella Maria BalestriniEirini PanagopoulouRaffaella SoaveEisa Tahmasbpour MarzouniRaffaella SoletiEishi AshiharaRafik NaccacheEitan FibachRafiou AgoroEizo MarutaniRaghavendra YadavalliEkaitz Errasti-MurugarrenRaghu VannamEkaterina A. LitvinovaRaghupathy VengojiEkaterina BaryshnikovaRahim AbdurEkaterina DadachovaRahul DeshpandeEkaterina FinkinaRahul GawriEkaterina I. MakarovaRahul KamathEkaterina KoltsovaRahul Mahadev ShelakeEkaterina N. GorshkovaRaimund TenhakenEkaterina N. LyukmanovaRaimund W. KinneEkaterina SilinaRaimundo GargalloEkaterina YurchenkoRaina RamnathEkram HossainRainer DetschElaine C. D. GonçalvesRainer NikolayElaine DunlopRainer SchreiberElaine EmmersonRaj BhullarEleana KontonasakiRaj KumarEleanor LeungRaj YadavEleanor P. WilliamsRaja Chandra ChakinalaEleanor TownsendRaja Gopal Reddy MooliEleftherios ChatzellisRaja VeerapandianEleftherios I. PaschalisRajaa El BekayElena A. KubarevaRajan AdhikariElena AlexandrovaRajan ChoudharyElena ApostolovaRajanikanth VangipurapuElena BertelliRajarshi ChakrabartiElena BoggioRajashekhar GangarajuElena BonciuRajbir SinghElena BonoraRajeev K. MehlotraElena BottaRajender S. VarmaElena BrunoRajendra KurapatiElena CampelloRajendra MehtaElena CancianiRajendran J. C. BoseElena CastroRajesh BhardwajElena CerradaRajesh DurairajElena ChiricozziRajesh DuttaElena CicheroRajesh KumarElena Crehuá-GaudizaRajesh MishraElena D. BazhanovaRaji AtchudanElena De FalcoRaji Paul GrewalElena De FeliceRajinder S. MannElena De MarchiRajiv DahiyaElena DeligianniRajiv Kumar KarElena Di PierroRajko KušecElena DiazRajkumar VenkatadriElena EfremenkoRajkumar VutukuriElena FicoRajmohan DharmarajElena G. GovorunovaRajneesh JaswalElena G. VarlamovaRajni VermaElena Gallo MacfarlaneRajnish SahuElena Garcia-FruitosRajtilak MajumdarElena GazzanoRaju AdhikariElena GiordanoRaju KhanElena GubarevaRakesh K. UpadhyayElena Guillén-GómezRakesh MajhiElena HilarioRakesh SrivastavaElena I. RyabchikovaRakkiyappan ChandranElena KaschinaRalf A. ClausElena KashubaRalf Anton BenndorfElena KirilovaRalf BlosseyElena KosenkoRalf KetterElena KozlovaRalf-Erik WellingerElena LaakmannRalph D. HectorElena LeychenkoRalph FrancesconeElena Lo PrestiRalph G. DepalmaElena Lopez-RodriguezRalph GräfElena LoretiRalph HübnerElena Maria VaroniRalph KnöllElena MariottoRalph Rolly GonzalesElena McBeathRalph TannerElena MontiRaluca IsopescuElena MourelatouRaluca Nicoleta Darie-NitaElena N. IkkonenRaluca Van StadenElena OrtonaRam BhusalElena PacellaRam PrasadElena PavlovaRama PulicharlaElena PeevaRamachandran PrakasamElena PiacenzaRamalingam Venkat Kalyana SundaramElena PiccininRamanathan SrinivasanElena PompiliRamces Falfan-ValenciaElena PopugaevaRamesh Krishnan RamasamyElena PrietoRamesh PothurajuElena R. SchiffRamesh S. YadavaElena S. BelykhRamganesh SelvarajanElena SaccoRamireddy BommireddyElena SalviRammohan V. RaoElena Sánchez LópezRamon EritjaElena SavvateevaRamon LorcaElena ScarpaRamon PamiesElena SommarivaRamón SantamaríaElena SvirshchevskayaRamon SunElena TchetinaRamona KuhnElena TenediniRamona PalomboElena TirròRamzi AjjanElena ToschiRan JingElena TsourdiRana Al-SadiElena TyuterevaRana El RawasElena V. TchetinaRanajay SahaElena V. ZemlyanskayaRandall BrennemanElena VallespinRando TuvikeneElena Victorovna DeinekoRandy NelsonElena ZenaroRanhua XiongEleni AnastasiadouRania AbutarboushEleni GolomazouRania HathoutEleni MavrogonatouRanjana MitraEleni PapanikolaouRanjit DeEleni VergadiRanko GacesaElenilson De Godoy Alves FilhoRaphael Jay WitorschEleonora AricòRaphael PlassonEleonora CominelliRaphael ThuillierEleonora GrespanRaphaël ThuillierEleonora LoiRaphaela FritscheEleonora MazzuccoRappa FrancescaEleonora PozziRaquel AlmeidaEleonora SocoRaquel AlvesEleonora TerreniRaquel BoiaEleonora VanniniRaquel ChavesEleonora VecchioRaquel CortésEleonore FröhlichRaquel De Melo BarbosaElettra BarberisRaquel Fernandez-GarciaElga BandeiraRaquel Guillamat PratsElia BariRaquel L. BernardinoElia GattoRaquel Martin VenegasElia GruesoRaquel Moreno LoshuertosElia RanzatoRaquel P. F. GuinéEliana AlvesRaquel RodriguesEliana Dell′olmoRaquel RomarEliana LacerdaRaquel RuivoEliana NutricatiRaquel SanchezElias A. KouroumalisRaquibul HasanElias AdriaenssensRasha Mohamed El NasharElias AlisaacRashid ManzoorElias ChristoforidesRasmus KjøbstedElias El-HabrRatnadeep MukherjeeElias SakellisRaudah LazimElide FormentinRaúl Capote-PuenteElide MantuanoRaúl Carrera CerritosEliette BonnefoyRaul DomínguezEliezer E. GoldschmidtRaúl EstévezElihu Aranday-CortesRaúl González-DomínguezElijah PetersenRaul MachadoElikanah Olusayo AdegokeRaul PironaElin ChorellRaul Sanchez-MuñozEline Pecho-VrieselingRavi Chakra TuragaElisa BelluzziRavi KumarElisa BenettiRavi Kumar CheedaralaElisa CabiscolRavi Kumar KomaravoluElisa CairrãoRavikanth NanduriElisa CeccheriniRavikumar ManickamElisa HerraezRavikumar PatelElisa MazzoniRavinder GoyalElisa OltraRavindra KumarElisa PanzariniRavindran BalasubramaniElisa RossiRavindran Caspa GokulanElisa Stellaria GrassiRavindranadh KoutavarapuElisa VergariRaviraj VankayalaElisa ZavattaroRay IlesElísabet Alcocer-GómezRaymond O′KeefeElisabet Stener-VictorinRaymond WongElisabete FernandesRazieh KebriaeiElisabeth Busch-NentwichRazmik MirzayansElisabeth ChristiansRazvan SocolovElisabeth GénotRebeca BustoElisabeth PinartRebeca MejíasÉlisabeth TraiffortRebecca A. DrummondElisabetta AldieriRebecca AlexanderElisabetta CapriniRebecca BaillieElisabetta CatalaniRebecca J. OrmsbyElisabetta CerbaiRebecca MetzlerElisabetta CianiRebecca WalkerElisabetta DonzelliRebeka ZsoldosElisabetta GrilloRebekah E. WhartonElisabetta MurruRebekah R. HeltonElisabetta OddoRebekka Vibjerg JensenElisabetta RazzuoliRedha TaiarElisabetta TabolacciReem T. AtawiaElisabetta VersinoReena KarthaElisabetta VolpeReet KurgElise DumontReetobrata BasuElise FouquerelRegina KasperekEliseo EugeninRegina M. DayElissavet NtemouRegina PodlasinElite PossikRegina R. ReimannEliyahu DremencovReginald GorczynskiEliza OpreaReginaldo G. BastosElizabete CândidoRegine Schneider-StockElizabeth A. BlaberRegis BobeElizabeth A. ThomasReidun ØvstebøElizabeth Ann L. EnningaReilly R. KayserElizabeth BrettReinaldo Sousa Dos SantosElizabeth C. CottrellReinhard DeppingElizabeth C. LedgerwoodReinhard GrisshammerElizabeth Carvajal-MillanReinhard KoppElizabeth JohnstoneReinhard LacknerElizabeth M. MartinReinhard MillerElizabeth Pavez LorieReinhard Schweitzer-StennerElizabeth R. GilbertReinhold J. MedinaElizabeth R. LusczekReinier AkkermansElizabeth R. VogelRéjean CoutureElizabeth Rendina-RuedyReka BarabasElizabeth RheaRéka MizseiElizabeth S. MaywoodRemco J. MolenaarElizabeth SokolRemenyik ÉvaElizabeth ThomasRemigiusz BąchorElizabeth VafiadakiRemigiusz WorchElizabeth W. BradleyRemington L. NevinElka R. GeorgievaRemo CampicheElke BarbezRemy ColinElke ButtRemy NicolleElla KimRémy PedeuxEllen H. KangRen WeiEllen SidranskyRenald DelanoueElli KoskelaRenan DanielskiElliott MufsonRenata CiccarelliElmar TräbertRenata GajElmar WolfRenata GrzywaElmina Mammadova-BachRenata JurkowskaElmira JalilianRenata KazimierczakElod NagyRenata KrupaElodie LafontRenata Matraszek-GawronElodie Olivares LouxRenata MikstackaEloisa RomanoRenata Novak KujundžićElona JuozaitytėRenata StawerskaElpidius J. RukambileRenáta SzabóElpis MantadakisRenata TeparićEls VerhoeyenRenata VaraljaiElsa ArcalisRenata Walczak-JędrzejowskaElsa C. ChanRenata ZajączkowskaElsa Cortés-MonteroRenate GehwolfElsayed ZakiRenato Alberto Rodrigues-PousadaElvira IngleseRenato BassanElvira RozhinaRenato BernardiniElwira SieniawskaRenato CoelhoEly Oliveira-GarciaRenato FroidevauxElżbieta BudziszRenato GennaroElżbieta Gumienna-KonteckaRenato SantosElżbieta KamyszRenato SeracchioliElzbieta KlewickaRenato Simões GasparElzbieta KolaczkowskaRenato V. La RoccaElżbieta Łodyga-ChruścińskaRenaud LegouisElżbieta Łopieńska-BiernatRene CorteseElżbieta MenaszekRené CsukElżbieta MillerRene G. FeichtingerElżbieta PatkowskaRené HägerlingElżbieta PłuciennikRene HuberElżbieta Rudolphi-SzydłoRene KoeffelElzbieta SalinskaRene St-ArnaudElzbieta SarnowskaRene VerdonkElżbieta SpeinaRene VidalElżbieta Wałajtys-RodeRené WenzlElżbieta WieczorekRenee GardnerElżbieta WierzbickaRenee JohnsonEmad Al-DujailiRenée L. EriksenEman A. AkamRenée M. Van Der SluisEmanuel SchneckRengasamy RamamoorthyEmanuel VamanuRenier Velez-CruzEmanuela BerrinoRen-Jun HsuEmanuela BottaniRenu SrivastavaEmanuela ChiarellaRenuka N. AttanayakeEmanuela CollaRenzo CorderaEmanuela PanniaReuben McGregorEmanuela StamponeReza HaghbakhshEmanuele AmadioReza KiaEmanuele GiurisatoReza MalekiEmanuella GrassilliReza ShafieiEmerson KrockRhiannon E. LloydEmese MezõsiRhimi MoezEmese PálovicsRhonda Bassel-DubyEmi HaladjovaRiad HaddadEmidio ScarpelliniRibhav MishraEmiel JanssenRicard AlbalatEmiel Van Der VorstRicard FarréEmil AlexovRicardo AraujoEmil BulatovRicardo Castro AlvesEmil CarlssonRicardo José BoschEmil KhisamutdinovRicardo Reyes RodríguezEmil MalucelliRicardo TejosEmil MladenovRicardo VardascaEmil N. SobolRiccardo ConcuEmil PaluchRiccardo Di CoratoEmil RudolfRiccardo Di MambroEmil ZakRiccardo FerraciniEmili BesalúRiccardo LeinardiEmili CidRiccardo ManganelliEmilia BigaevaRiccardo NevolaEmilia BramantiRiccardo PapaEmilia CirilloRiccardo RigoEmilia GalperinRiccardo Zenezini ChiozziEmilia KozlowskaRicha SachanEmilia ManoleRichard ArmEmilia Rita SzabóRichard BrightEmilia VassilopoulouRichard CrippsEmilia WilmowiczRichard D. NoyesEmilia ZgorzynskaRichard DahlEmilian Florin MoșneguțuRichard E. HonkanenEmiliana GiacomelloRichard EgelEmiliano DallaRichard FairlessEmilie LeguéRichard FinnellEmilie Talagrand-ReboulRichard FunkEmilie WidemannRichard GallonEmilie WientjesRichard HamplEmilija ZdravevaRichard HrabalEmilio CervantesRichard Inho JohEmilio CusanelliRichard JonesEmilio J. VélezRichard KeepEmilio LeconaRichard Kumaran KandasamyEmilio VareaRichard LebaronEmily M. RochaRichard MeehanEmily P. McCannRichard P. FahlmanEmily W. SunRichard PestellEmma GachomoRichard R.-C. WangEmma MarczyloRichard SchasfoortEmma R. JamesRichard SchulzEmma RybalkaRichard SheardyEmma SiereckiRichard SindenEmmanouil D. TsochatzisRichard Steven PappasEmmanouil KapetanakisRichard TomasiniEmmanouil P. BenisRichard TrohmanEmmanuel BourdonRichard Van KriekenEmmanuel J. FavaloroRichard W. BrillEmmanuel M. PapamichaelRichard WebbEmmanuel PanterisRichard WebsterEmmanuel PayenRichard ZimmermannEmmanuel TaillebourgRick I. MeijerEmmanuel TetaudRick KampsEmmanuele De VendittisRidwan P. PutraEmmanuelle BourneufRie Shimizu-InatsugiEmmanuelle LeratRigini PapiEmoke PallRigumula WuEmpar ChenollRiikka MartikainenEmyr BakkerRiikka PeltomaaEn MengRikhav P. GalaEneli HärkRikio SuzukiEnes AkyuzRiku KawasakiEng Guan ChuaRina BandopadhyayEng Kuan MooRina KamenetskyEnikő BaloghRina RosenzweigEnikő BodóRinaldo PellicanoEnikö KállayRinkaj GoyalEnni MarkkanenRintaro NoroEnnio ProsperiRinu KooliyottilEnoch A. MensahRio BoothelloEnquan XuRio Ryohichi SugimuraEnric EspelRishi Man ChughEnric I. CanelaRita B. SantosEnrica StrettoiRita BenítezEnrica TorrettaRita CasadioEnrica UrciuoliRita CasadonteEnrico BaruffiniRita CowellEnrico BodoRita De MatteisEnrico BraccoRita FragosoEnrico ConservaRita KissEnrico GiordanRita MarreirosEnrico LucarelliRita MorigiEnrico Maria DaldelloRita NastiEnrico Maria SuraceRita ParoniEnrico MarinelliRita Payan-CarreiraEnrico MilloRita PuglisiEnrico PerrinoRita SattlerEnrico RadælliRita ShiangEnrico RizzarelliRita TurnaturiEnrico UllmannRita ValenzuelaEnrico Vito PerrinoRitesh SrivastavaEnrique BalderasRitesh TandonEnrique Domínguez-ÁlvarezRitesh ThekkedathEnrique Gomez GomezRitwik DattaEnrique Gomez-PiñeroRiyazuddin RiyazuddinEnrique J. CobosRizwan RomeeEnrique LanuzaRob A. GrutersEnrique Meléndez-HeviaRob C. I. WüstEnriqueta AnticóRob FowkesEnza LonardoRob HoebenEnza PalazzoRob WhiteEnzo BerardescaRobert A. BudinskyEnzo IerardiRobert A. HorowitzEphrem EngidaworkRobert Arthur KratzkeEran Van VeldhuisenRobert B. PernaEric A. AlbrechtRobert B. RuckerEric A. EppingRobert BajgarEric A. P. HerleniusRobert BatoriEric B. KmiecRobert BestEric BattagliaRobert BlanvillainEric BlommeRobert BrinsonEric BoncompagniRobert C. FoehringEric C. LongRobert ChiltonEric D. JensenRobert CzarnomysyEric D. RossRobert EckelEric DefrancqRobert EckenstalerEric DorisRobert EferlEric GalianaRobert EkiertEric GhigoRobert F. CasperEric GilsonRobert G. BestEric GontierRobert GilkersonEric HajduchRobert GouldEric HervouetRobert Haensel-HertschEric HuetRobert HorvathEric KuhnertRobert I. SeedEric LargyRobert J. BarrettEric Le BourgRobert J. PalmerEric MasRobert J. ScarboroughEric MetzenRobert JohnsonEric PasmantRobert KammererEric RuellandRobert KasprzakEric S. HoRobert KellyEric S. PeeplesRobert KissEric Santoni-RugiuRobert KleszczEric Scott SillsRobert KrysiakEric W. OttesenRobert L. CarloneEric WesthofRobert L. MedcalfEric YagerRobert L. MeiselErica BazzanRobert L. RubinErica GentilinRobert LindnerErica M. SparkenbaughRobert LukowskiErica NovoRobert M. BadeauErica SalvatiRobert M. DietzErica StaurenghiRobert M. LafrenieErich PiovanRobert N. HelsleyErich SchwarzRobert NashErick S. LebrunRobert PhilibertErick Sierra-CamposRobert PhillipsErik A. C. WiemerRóbert PókaErik BehringerRobert PosseeErik GoormaghtighRobert R. CrichtonErik JohnsonRobert RoškarErik KudelaRobert S. YoungErik T. YuklRobert SempleErik VijgenboomRobert StawskiErika A. TaylorRobert T. MeansErika CioneRobert TureskyErika Di BiaseRobert Van DomselaarErika JorgeRobert Van WaardenburgErika López-ArribillagaRobert W. CoppockErika PalmieriRobert WardErika RicciRobert WeinzierlErika SabellaRobert WeissEriko KatsutaRobert WojtyczkaErin AlmandRoberta AngioniErin McClellandRoberta BenfanteErin RehrigRoberta BortolozziErjin ZhengRoberta CazzolaErkko YlösmäkiRoberta ChiaraluceErlinda TheRoberta Di PietroErmenegilda VitaleRoberta ForlanoErnani PintoRoberta FuscoErnestina Marianna De FrancescoRoberta GonnellaErnesto Martínez-MartínezRoberta ImperatoreEros Di GiorgioRoberta OkamotoErrol HassanRoberta PireddaErshuai ZhangRoberta RigolioErwan DelbarreRoberta RizzoErwan MortierRoberta SaleriErwin BrosensRoberta SchellinoErwin D. SchleicherRoberta SqueccoEryn L. WerryRoberta TaralloEsa KorpiRoberta TassoEsmaeil AmiriRoberta ToroEsmaeil EbrahimieRobert-Alain ToillonEsmail LutfiRoberto A. BotoEsmeralda Ramirez-PeñaRoberto AiolfiEsmerilda G. DelicadoRoberto BaroneEsperanza DíazRoberto BenelliEsperanza Martín-SánchezRoberto CampagnaEspinosa Martín Juan CarlosRoberto CannataroEstanis NavarroRoberto Cardenas ZunigaEsteban Obrero-GaitánRoberto CastiglioneEstéfani García-RíosRoberto CesareoEstefanía MorenoRoberto ChiesaEstefania NuñezRoberto ChimenzEstefany Ingrid Medina-ReyesRoberto CiarciaEstela JacintoRoberto ColasantiEstela Maldonado BautistaRoberto ColluEster BoixRoberto CostaEster Leno-DuránRoberto Da RosEster Marco-NoalesRoberto De La Torre-MartinezEstera RintzRoberto FeliceEsterina FazioRoberto Fernandez-LafuenteEsther García-MoralesRoberto FiorenzaEsther LlopRoberto GermanoEsther Serrano-PertierraRoberto GiovannoniEsther StoeckliRoberto GramignoliEsther TamayoRoberto La RoccaEstíbaliz AlegreRoberto LandeEswar ShankarRoberto LigroneEszter DuczaRoberto MarottaEszter Mária HorváthRoberto Martínez BeamonteEtana PadanRoberto OleariEthan David CohenRoberto PiluEthan L. MorganRoberto PizzalaEthan MorganRoberto RamoniEthan StrattanRoberto RizzoEtienne ChalletRoberto RonaEtienne DucrotRoberto RoncaEtsushi KurodaRoberto SalaEttayapuram Ramaprasad Azhagiya SingamRoberto SitiaEttore VarricchioRoberto SorrentinoEudald CasalsRoberto Vicente Gozalbo RoviraEugen GheorghiuRoberto ZefferinoEugen Mircea AnitasRobin CurtisEugene G. MaksimovRobin KettelerEugene J. BarrettRobin Lewis CooperEugene KucharzRobin SebastianEugene S. KandelRobin StanleyEugene SverdlovRobyn HallEugenia BezirtzoglouRobyn RebbeckEugénia GallardoRocchetti VincenzoEugenia KarousouRocco Di GirolamoEugenia Marqués-LópezRoche JaneEugenia V. GurevichRochelle C. J. D′SouzaEugenijus ŠimoliūnasRochelle TixeiraEugenio BertelliRocio Alejandra Chavez-SantoscoyEugenio MaioranoRocio BautistaEugenio MorelliRocío BenitoEugenio VilanovaRocío González-SolteroEugeny Yu. TalanovRocío Guzmán RuizEui-Bae JeungRocío Martínez De PablosEuikyung KimRocío Ruiz LazaEui-Seok LeeRodica BalasaEulalia BazánRodica ZavoianuEulàlia GenescáRodica-Mihaela DinicaEun Jeong ParkRodolfo GattoEun Seong HwangRodolfo NegriEunate Gallardo-VaraRodolfo ZentellaEun-Bin BaeRodrig MarculescuEunbit KimRodrigo A. LoiolaEung-Kwon PaeRodrigo CasasnovasEunhee KimRodrigo Coutinho De AlmeidaEun-Hee KimRodrigo De AlmeidaEunice CarrilhoRodrigo FerreiraEunju KangRodrigo Herrera-MolinaEunju ParkRodrigo Moore-CarrascoEunkyoo OhRodrigo ValenzuelaEun-Kyung LimRoger AbounaderEun-Mi HurRoger BiringerEun-Woo LeeRoger ChongEusebio ChiefariRoger GrandEustace Y. FernandoRoger LengEustachio TarascoRoger LiEva A. TurleyRoger Sanchis-GualEva Alicja Rog-ZielinskaRoger ThilmonyEva AndreuzziRoger WilliamsEva Budisova ŠvandováRoh HyeongJinEva BussalleuRohit KumarÉva CsőszRohit SrivastavaEva FenyvesiRohita SinhaEva Fischer-FodorRohitash JamwalEva Garcia-RuizRok GaspersicEva GijbelsRok KosirEva HorvathovaRoksana MarkiewiczEva KatonaRoland BückerEva KnuplezRoland KlassenEva KudovaRoland LangEva M. Medina-RodríguezRoland MalliEva María Rivero-BucetaRoland P. BouretteEva Mischak WeissingerRoland StauberEva Nozik-GrayckRoland Wedlich-SöldnerEva ObermayrRolando PasquarielloEva PluhařováRolf A. BrekkenEva RealiRolf HeumannEva ŠatovićRolf SokildeEva SykováRolf TeschkeÉva SzabóRolf TurkEva SzokeRomain GastineauÉva SzökőRomain GuinamardEva TvrdaRomain Veyron-ChurletEva VarallyayRomain Vuillefroy De SillyEva ŽerovnikRoman B. LesykEvagelos GikasRoman DeevEvaldo FaviRoman GardlikEvangelia AvramidouRoman HrstkaEvangelia ChronopoulouRoman LesykEvangelia KoutelouRoman LysiukEvangelia LivaniouRoman SmolarczykEvangelista LauraRoman SurmenevEvangelos CholongitasRoman TrochimczukEvangelos D. KarousisRomana FatoEvangelos HristoforouRomana SchirhaglEvangelos KaragiannisRomana SlamberovaEvangelos KoustasRomana VulturarEvangelos ZoidisRomano DemicheliEve PécheurRomeo T. CristinaEvelyn OrsoRomina Alina MarcEvgenia GerasimovskayaRomina NassiniEvgenia Korzhikova-VlakhRomualda Bregier-JarzębowskaEvgenia MitsouRon KedemEvgenia PapakonstantinouRon OrbachEvgenia PechkovaRon SaulnierEvgeniy S. BalakirevRonald B. BrownEvgeniya PushchinaRonald J. TrottaEvgeniya V. EfimovaRonald M. A. KnegtelEvgeny E. AndronovRonald P. TaylorEvgeny E. BezsonovRonald WandersEvgeny ErmakovRonan MurphyEvgeny ShirshinRong HuangEvgeny ZatulovskiyRong ZhouEvguenii VinogradovRongcong LuoEvnouchidou IriniRongkung TsaiEvzen BouraRong-Kung TsaiEwa A. GrzybowskaRong-Nan HuangEwa BartnikRonit MandalEwa Brzezińska-BłaszczykRonny HaenoldEwa ChudzińskaRony SegerEwa DudzińskaRory KoenenEwa Gibuła-TarłowskaRosa AlduinaEwa GlowinskaRosa DireitoEwa GrzybowskaRosa Figueroa-BalderasEwa IgnatowiczRosa FontanaEwa Iżycka-ŚwieszewskaRosa M. BlancoEwa Joanna Hanus-FajerskaRosa MancinelliEwa KorzeniewskaRosa María ChicaEwa KurczyńskaRosa Maria De Sá PerestreloEwa M. FurmańczykRosa Maria Mateos BernalEwa M. KalembaRosa NogueraEwa M. UrbańskaRosa PascaleEwa MarcinkowskaRosa PerestreloEwa Miller-KasprzakRosa PurgatorioEwa NazarukRosa SacedónEwa Nowakowska-ZajdelRosa SorrentinoEwa OleńskaRosa TundisEwa PochećRosalba SiracusaEwa Rajpert-De MeytsRosales-Hernández Martha C.Ewa RopelewskaRosalia BertorelliEwa SikoraRosália Mendez-OteroEwa StodolakRosalin MishraEwa SwiezewskaRosalind DalefieldEwa Szczepanska-SadowskaRosalind Lindy JeffreeEwa TomaszewskaRosanna CapparelliEwa TotońRosanna Di PaolaEwa WaszkiewiczRosanna InturriEwa Wiesik-SzewczykRosanna MallamaciEwa WolińskaRosaria AcquavivaEwelina Czyzewska-DorsRosaria Anna PiccaEwelina DratkiewiczRosaria ArconeEwelina GrywalskaRosaria MeccarielloEwelina HallmannRosaria SantoroEwelina KrólRosaria ScudieroEwelina Warzych-PlejerRosario AmatoEwoud B. CompeerRosario BaroneEzgi Eylül BankogluRosario DonatoF. Raquel MaiaRosario Francesco DonatoF. Scott HallRosario GulinoFabian IlleRosemary UnderhillFabian ItelRosemary VukovicFabián Martínez-HernándezRoshan SatangeFabian MohrRoshini RamachandranFabian SchumacherRoss CaganFabiana ConciatoriRoss ChelohaFabiana CrispoRossella BrunoFabiana LabancaRossella CannarellaFabiana PandolfiRossella Di GiaimoFabiana SupertiRossella LianiFabiao YuRossella MieleFabien Fontaine-ViveRossella PaoliniFabien ForestRossella PariniFabien SalaünRossella SvigeljFabienne E. PoulainRossella TomaiuoloFabio Arturo IannottiRossella VendittiFabio CoppedèRoustem KhazipovFabio Di NardoRoxana Cristina PopescuFabio GanazzoliRoxana JijieFabio GrizziRoxana Maria NemeșFabio GuoloRoxana RacoviceanuFabio MonzaniRoxana VidicanFabio NicoliniRoxana-Maria AmărandiFabio PasinRoxane PouliotFabio PolticelliRoy LarsenFabio RizzoRuan Hai BinFabio RomerioRuben C. PetreacaFabio SallustioRubén CalotoFabio VivarelliRubén Cebrián CastilloFabiola GiudiciRubén Darío Castro-TorresFabiola VilasecaRubén G. ContrerasFabrice AntignyRubén Gómez-SánchezFabrice CognasseRuben Hernandez-AlcocebaFabrice DarcheRuben Manuel Luciano Colunga BiancatelliFabrice HombléRubina JibranFabrice LecuruRubina ShaikhFabrice SaezRuby LawFabrizia BonacinaRuchika AnandFabrizio AmbrosinoRuchika BajajFabrizio AngiusRuchir PriyadarshiFabrizio AnniballiRuchira ChatterjeeFabrizio BertelloniRuchira SinghFabrizio CarboneRuczyński JarosławFabrizio CartaRüdiger HorstkorteFabrizio CilloRüdiger KlapdorFabrizio DamianoRüdiger LandFabrizio FontanaRudimar Luiz FrozzaFabrizio GardoniRudolf A. WernerFabrizio GrassiRudolf Hans TinnebergFabrizio ManettiRudolf HergesheimerFabrizio MontecuccoRudolf LikarFabrizio PinRudy J. ValentineFabrizio SantoroRuediger RudolfFabrizio SignoreRuediger ThulFabrizio TommasoRuggero CadossiFabrizio VeticaRuggero La RosaFabrizio VincenziRugivan SabaratnamFabrizio VitaleRui ChenFacundo RuizRui ChengFaheem Shahzad BalochRui FalachoFahim AhmadRui FaustoFahimeh ShahinniaRui HenriqueFaith A. KwaRui M. Borges Dos SantosFalco ReissigRui M. Gil Da CostaFalk LiebnerRui M. S. CruzFalk NimmerjahnRui PereiraFalko LangeRui SongFamulari AntoninoRui VitorinoFan ZhangRui WangFang (Rose) ZhuRuifeng HuFangfeng YuanRuilong ShengFani ChatzopoulouRuisheng WangFanny LegrandRujuan DaiFaouzi SalibaRumen IvanovFarah R. ZahirRumyana KarlovaFarhad HossainRumyana MarkovskaFarhad Masoomi-AladizgehRun ZhangFarhat KhanimRundk HwaizFarid El-KasmiRune MatthiesenFarid RahimiRupendra ShresthaFarida BenhadouRupert TscheliessingFaris MujezinovićRupesh DeshmukhFarjana RowtherRupesh KariyatFarnaz ShamsiRupesh Kumar SinghFarooq SyedRupesh TayadeFarrukh AqilRusan CatarFarzad PakdelRustem ZairovFarzana SabirRuta MucenieceFasil Tekola AyeleRuth A. SchwalbeFatemah Sadeghpour HeraviRuth HalabanFatima CvrčkováRuth HemmersbachFátima GärtnerRuth KnüchelFatima MacedoRuth ParksFatima MartinsRuth PrasslFátima NogalesRuud A. W. VeldhuizenFátima O. MartinsRuud P. M. DingsFatima O. SmagulovaRuwei LinFawzia Bardag-GorceRuzanna DjavadianFaxing WangRwik SenFaye MorrisRyad ZemouriFazlurrahman KhanRyan A. HenryFederica CampoloRyan AshleyFederica CirilloRyan C. GimpleFederica D′AmicoRyan D. SullivanFederica De LiseRyan J. AndresFederica Di SpiritoRyan LalumiereFederica DomatiRyan SeipkeFederica FerroneRyhanen SamppaFederica FiliceRyo FujimotoFederica FogacciRyo HamaiFederica FregnanRyo MaekawaFederica LopesRyo MisakiFederica MagagnoliRyoichi AsakaFederica MoraniRyoko SaitoFederica SangiuoloRyoma KamikawaFederica SarnoRyong Nam KimFederica SodanoRyota MasuzakiFederica SpinaRyota MatsuoFederico FogolariRyota ShizuFederico FontanaRyousuke SatouFederico LazzaroRyszard AmarowiczFederico LussanaRyszard KuligFederico ManaiRyszard LobinskiFederico Manuel GiorgiRyszard PlutaFederico N. SoriaRyuji HamamotoFederico Paolini PaolettiRyuma HaraguchiFederico PercheRyuya FukunagaFederico PozzoRyza A. PriatamaFederico VerdeS. Priya NarayananFederico VitaS. RangelovFedor NovikovS. S. SimakovFee KluppS. Srivatsan EriFei WangS. T. J. TsangFeifei AnSaad A. AmerFeilong LiSaad El-Din HassanFeiting HsuSaak V. OvsepianFelice LorussoSaba TabasumFelice MastroleoSabato D′AuriaFelicia AntoheSabbatini MaurizioFeliciana Real-FernándezSabina GaliniakFeliczak-Guzik AgnieszkaSabina GalusFelipe Cesar Ferrarezi BeckedorffSabina M. JanciauskieneFelipe E. Reyes-LópezSabina RanjitFelipe Martinez-PastorSabina SevcikovaFelipe ParedesSabine IvisonFelipe PasseroSabine Jung-KlawitterFelipe Penagos-TabaresSabine KässmeyerFelipe Vadillo-OrtegaSabine Kuchler-BoppFelisa Reyes-OrtegaSabine MaiFelix AmissahSabine MazerbourgFelix BehlingSabine PellettFelix ClanchySabine Sator-KatzenschlagerFelix GoeserSabine WindhorstFelix Javier Jiménez-JiménezSabino Aurelio BufoFelix Jimenez-RondanSabrina BattistaFélix Juan Martínez RivasSabrina BréchardFelix LockerSabrina CorbettaFelix NitschkeSabrina EhnertFélix PicazoSabrina HuppFelix SchmittSabrina LisiFelix WezelSabrina ManniFemi J. OlorunnijiSabrina Rahman ArchieFemke HeindryckxSabrina TaitFeng LiSabrina ValenteFeng WangSabryna Barbara JunkerFeng XuSabyasachi DashFeng XueSacha CoeselFengchao LangSacha EscamezFeng-Chou TsaiSachiko SekiyaFeng-Lin YenSachiko Toma-FukaiFengzhi LiSachin K. SamuchiwalFerdinando BrancaSachin TeotiaFerdinando Carlo SassoSachio TakenoFerdinando CercielloSachio TsuchidaFerdinando FebbraioSadanand PandeyFerdinando FiorinoSadanori AkitaFerdinando MannelloSae-Byuk LeeFerdinando NicolettiSaeid GhavamiFerdinando PaternostroSafeer AhmedFerdy R. Van DiemenSafra Rudnick-GlickFerenc BogárSagrario Martin-AragonFerenc DomokiSahdeo PrasadFerenc ErdődiSahib ZadaFerenc GallyasSai ChittiFerenc LezsovitsSaid OulghaziFerenc PappSaiful IslamFerenc SiposSaigopalakrishna S. YerneniFerenc TóthSaikat BalaFernández-Ocaña AnaSaima ImranFernando BartolomeSaivageethi NuthikattuFernando C. BaltanásSaji UthamanFernando Capela e SilvaSajid ShokatFernando CardonaSajjad AhmadFernando CardosoSakina Mhaouty-KodjaFernando De CastroSaktimayee Mitra RoyFernando EcheverriSalah AmashehFernando FerreiraSalar ShaafFernando G. VieiraSalih VezirogluFernando GallardoSalman GoudarziFernando Gómez-HerrerosSalman IslamFernando López-GatiusSalvador Francisco AliñoFernando Lopitz OtsoaSalvador IborraFernando M. Araujo-MoreiraSalvador PérezFernando MonroySalvatore ChiantiaFernando ParenteSalvatore ChirumboloFernando ReyesSalvatore D. BlairFernando RodríguezSalvatore EspositoFernando Shintate GalindoSalvatore FeoFernando SotoSalvatore FiorinielloFernando Vidal-VanaclochaSalvatore GalloFernando VonhoffSalvatore Giovanni De SimoneFernão D. MagalhãesSalvatore ManganoFerreira FernandoSalvatore Nicola BertuccioFerri Elida NoraSalvatore PassarellaFerry MelchelsSalvatore PiroFiammetta PiersigilliSalvatore SantamariaFidel Gonzalez TorralvaSalvatore ScaccoFidel Lozano-ElenaSalvatore SotgiaFilimon AncaSamantha C. KarunarathnaFilip ŠeniglSamantha DonsanteFilipa GonçalvesSamantha S. SoldanFilipa MarceloSamantha YeligarFilipe ElvasSambuddha BasuFilipe Hobi Bordón SosaSameer ChavanFilipe PereiraSameer VarmaFilipe PintoSameera WickramasingheFilippo CiabrelliSamer HaidarFilippo CottigliaSamia A. ElseginyFilippo DragoSamikshan DuttaFilippo Maria SantorelliSamir BarghoutFilippo MarianoSamir GorasiyaFilippo MiglioriniSamo KraljFilippo MolicaSampurna ChatterjeeFilippo PieriniSamrita DograFilippo RossignoliSamsad RazzaqueFilippo TorielloSamuel BruFilippo TorrisiSamuel E. AdunyahFilippos KoinisSamuel E. SchrinerFilippos TriposkiadisSamuel Fernández-ToméFillipe Luiz Rosa Do CarmoSamuel FrankFillipos SofosSamuel HudsonFilomena AdegaSamuel M. SilvestreFilomena De NigrisSamuel Peña-LlopisFilomena FezzaSamuel SilvestreFilomena MottolaSamuel X. ShiFilomena NapolitanoSamuele IesariFina Martínez-SolerSamuele MaramaiFinn Edler Von EybenSamuil UmanskyFiona E. HarrisonSamuli RautavaFiona HenriquezSanaa EissaFiona HoustonSanchari BhattacharyyaFiona Jane RadcliffSanda AndreiFiona UkkenSanda Mihaela PopescuFiore Pasquale NicolettaSandeep AmetaFiorella Lo SchiavoSandeep Kumar BarodiaFiorenza RancanSandeep Kumar MishraFiras KobeissySandeep RajputFlavia BiamonteSandeep SinghFlávia Leão BarbosaSandeep ThannaFlavia MerigoSandesh PanthiFlavia RadognaSandip A. GhugeFlavia Souza-SmithSandip Ashok SonarFlavia V. GouveiaSandipan DattaFlavio FigueiraSandor BenyheFlavio GiordanoSándor Kunsági-MátéFlavio LicciulliSandra AlvesFlavio MainaSandra Beer HammerFlavio RizzolioSandra CorreiaFleming Martinez RodriguezSandra DonniniFlora N. BalievaSandra FilippiFlóra SzeriSandra Garcia-GallegoFlorence BesseSandra GhelardoniFlorence BuseyneSandra LeiszFlorence Mahuteau-BetzerSandra LejnieceFlorence MusSandra Lia Do AmaralFlorenci V. GonzálezSandra Lopez-AvilesFlorencia CavodeassiSandra M. Martin GuerreroFlorent MorfoisseSandra MarmiroliFlorentia PapastefanakiSandra PintoFlorentina GateaSandra PucciarelliFlorentina MateiSandra RugonyiFlorentina Monica RadulySandra SanchoFlorentina PluteanuSandra SkujaFlorentina-Daniela MunteanuSandra SousaFlorian BeaumatinSandra TorresFlorian BockSandra TuyaertsFlorian DuclotSandra V. FernandezFlorian FreudenbergSandrina Nóbrega-PereiraFlorian GesslerSandrine BarbauxFlorian NachonSandrine MarchettiFlorian PrüllerSandrine MorelFlorian SennlaubSandro La VigneraFlorian WeinbergerSandro OrrùFloriana VolpicelliSandro RengoFlorin George HorhatSandro SonninoFlorin OanceaSanford I. BernsteinFlorin OnisorSang Ah YiFlorin TriponSang Hoon KimFlorina TeodorescuSang SuhFloris BosveldSang Won ParkFodor MariettaSang Woo KimFoivos PerakisSangBae GiForough JahandidehSangbum ParkFoteinos-Ioannis DimitrakopoulosSangeetha HareendranFotini SkopouliSang-Han LeeFotis BaltoumasSang-Ho JoFranca AnglaniSangho RohFranca EspositoSangmi LeeFranca FagioliSang-Mok LeeFranca MarinoSangram SamalFranca R. GueriniSang-Ryong LeeFrances M. LeslieSangsu BaeFrances T. YenSang-Tae KimFrancesc García GonzaloSang-We KimFrancesc Jiménez AltayóSangwon KimFrancesc MoresoSang-Wook KangFrancesc ViñalsSanja Kostadinovic VelickovskaFrancesc Xavier AvilésSanjairaj VijayavenkataramanFrancesc Xavier GuixSanjana DayalFrancesca AgriestiSanjay MarasiniFrancesca ArfusoSanjay PatelFrancesca BianchiniSanjoy BhattacharyaFrancesca BosciaSanjoy PaulFrancesca Boscolo SesilloSankar JayanthiFrancesca CalabreseSanoj RejinoldFrancesca CantiniSanta CirmiFrancesca CasciaroSantanu MondalFrancesca ChiariniSantanu MukherjeeFrancesca CianiSante Di GioiaFrancesca De FeliceSanti SpampinatoFrancesca De LucaSantiago BonanadFrancesca De SantisSantiago Garcia-GrandaFrancesca FumagalliSantiago Garcia-VallveFrancesca GadoSantiago HaaseFrancesca GattoSantiago J. BallazFrancesca GranucciSantiago Ramón-MaiquesFrancesca GrazianoSantina BruzzoneFrancesca GuarinoSantina CutrupiFrancesca IommelliSanto Raffaele MercuryFrancesca LeonardiSantosh BashyalFrancesca Luisa ConfortiSantosh DhakalFrancesca LunardiSantosh GhoshFrancesca MackenzieSantosh Kumar SinghFrancesca MagheriniSantosh LamichhaneFrancesca MalvanoSantosh MisalFrancesca ManciantiSantosh PandaFrancesca MancusoSantosh PanditFrancesca MontaroloSantosh UpadhyayFrancesca MoretSao-Jose CarlosFrancesca MottarliniSaowaluck TibprommaFrancesca NazioSara AlbarellaFrancesca PalladinoSara BachillerFrancesca PeruzziSara BelloliFrancesca PesciniSara BeneditoFrancesca PrestoriSara BernardiFrancesca ReggianiSara BernardoFrancesca ResentiniSara BoenziFrancesca RicciSara CaceresFrancesca RizzelloSara Casado ZapicoFrancesca Romana MariottiSara De MartinFrancesca S. ForiniSara Duarte-SilvaFrancesca SalamannaSara FateixaFrancesca SantilliSara Franco OrtegaFrancesca SciandraSara FulignatiFrancesca SciontiSara GagoFrancesca SerioSara González-RodríguezFrancesca SilvagnoSara GoodacreFrancesca SistoSara Guerrero-AspizuaFrancesca TrojsiSara HamzelouFrancesca VasileSara Huerta-YépezFrancesca ViazziSara J. CooperFrancesca ZitoSara LindenFrancesco AcerbiSara N. RichterFrancesco AgostiniSara NavaFrancesco AlbanoSara PączekFrancesco AsnicarSara PagottoFrancesco BaschieriSara PerteghellaFrancesco BellantiSara R. Martins-NevesFrancesco BellinatoSara ReisFrancesco BellomoSara Remuzgo-MartínezFrancesco BennardoSara RezzolaFrancesco BertoliniSara S. McCoyFrancesco BiancoSara SalucciFrancesco BoccellatoSara SimoniniFrancesco CacciolaSara TramontiniFrancesco CadarioSara TucciFrancesco CammarataSarabjit MastanaFrancesco CanonicoSarada Preeta KalainayakanFrancesco ChemelloSaradha VenkatachalapathyFrancesco ChianconeSarah B. GittoFrancesco D′AmicoSarah BeckFrancesco De FrancescoSarah DerksFrancesco De RosaSarah E. FritzFrancesco De SanctisSarah E. JacksonFrancesco Del GiudiceSarah HedtrichFrancesco Di GennaroSarah Hurtado-BagèsFrancesco FeoSarah K. WestburyFrancesco FerragutiSarah MazzottaFrancesco FiniSarah SerticFrancesco FornaiSarah ShigdarFrancesco FusoSarah WatsonFrancesco LeonettiSarang TarteyFrancesco LodolaSaravanan JanakiramFrancesco LopsSarbjit SinghFrancesco MaininiSareh ZhandFrancesco ManfrediSarel HalachmiFrancesco MannelliSarfraz ShafiqFrancesco MantegazzaSari MattilaFrancesco MassariSarit LarischFrancesco MercatiŠárka LehtonenFrancesco MerlinoSarmistha TalukdarFrancesco MiceliSarmiza Elena StancaFrancesco MoraSaša ĐurovićFrancesco MorenaSaša FrankFrancesco MorraSascha WaidmannFrancesco NappiSashka KrumovaFrancesco NicotraSaska IvanovaFrancesco NozzoliSaskia FreytagFrancesco OrienteSathiyaseelan AnbazhaganFrancesco OrziSatish DhingraFrancesco Paolo FanizziSatish KumarFrancesco Paolo TambaroSatish RainaFrancesco PesceSatish ToteyFrancesco PirainoSatoru KaseFrancesco PollariSatoru SuzukiFrancesco RuggeriSatoru TsugawaFrancesco RusconiSatoshi FukushimaFrancesco S. DioguardiSatoshi InoueFrancesco Saverio SorrentinoSatoshi IreiFrancesco ScavelloSatoshi KameshimaFrancesco SessaSatoshi KishigamiFrancesco SignorelliSatoshi KomatsuFrancesco StellatoSatoshi KumakuraFrancesco StomeoSatoshi MigitaFrancesco StratiSatoshi MitaraiFrancesco SunseriSatoshi SaitoFrancesco TampieriSatoshi SanoFrancesco TavantiSatoshi TakahashiFrancesco TovoliSatoshi TanakaFrancesco VanziSatu HepojokiFrancine Perrine-WalkerSatu KuureFrancis J. OsongaSaturnino Marco LupiFrancis K. YoshimotoSatyabrata NandaFrancisca DiasSaul Herranz-MartinFrancisca Ferrer-MarínSaulius GrigaleviciusFrancisca Pereira De MoraesSaulius RočkaFrancisco A. ArrebolaSaulo TintinoFrancisco A. TauskSaurabh ChaudharyFrancisco AlvesSaurabh SrivastavaFrancisco BustosSaurav Kumar JhaFrancisco Carrillo-SalinasSavalan Babapoor-FarrokhranFrancisco CentenoSavin TreantaFrancisco Eliseo Olucha BordonauSavina ApolloniFrancisco EstévezSavita SharmaFrancisco Fernandez-CamposSavvas ChristoforidisFrancisco Fueyo GonzálezSavvas LampridisFrancisco Gabriel Vázquez-CuevasSawsan A. ZaitoneFrancisco García-CózarSayan ChakrabortyFrancisco García-del PortilloSayan MondalFrancisco Garcia-SanchezSayantan JanaFrancisco Guillen-GrimaSayyeda Marziya HasanFrancisco J. BlancoScavello FrancescoFrancisco J. EnguitaSchedel MichaelaFrancisco J. VizosoSchreiner WolfgangFrancisco Javier CuberoScott A. HolmesFrancisco José González-MineroScott C. SchuylerFrancisco José Ostos MarcosScott ForthFrancisco Lázaro-DiéguezScott I. SimonFrancisco LeonScott McCombFrancisco LlaveroScott PlafkerFrancisco M. Martin-SaavedraScott RussellFrancisco M. VegaScott T. WoodFrancisco Martínez-AbarcaSe Jin ParkFrancisco MeijideSe Kyoo JeongFrancisco MesaSeah LimFrancisco MuruzabalSean BlamiresFrancisco RiveroSean BulleyFrancisco SolanoSean C. NewtonFrancisco TorrensSean ChiaFrancisco Triana MartinezSean Chun Chang ChenFrancisco Vasques-NóvoaSean D. KodaniFrancisco VerdeguerSeán O′LearyFranciska Tóthné BogdányiSebastiaan MeijsingFranck GallardoSebastian BeyerFranck MartinSebastian BjörklundFranck PanabieresSebastián Demyda-PeyrásFranck VerrecchiaSebastian GnatFranco BassettoSebastián HormigoFranco FulcinitiSebastian HuthFranco MutinelliSebastian IbenFrancois Alhenc-GélasSebastian KirchnerFrançois CasasSebastian KrugFrançois ChevalierSebastian P. NischwitzFrancois CoutleeSebastian PiłsykFrançois GuerineauSebastian PohlFrançois KrierSebastian PonsFrançois NiyonsabaSebastian RademacherFrançoise ArnaudSebastian SchloerFrançoise Jacob-DubuissonSebastian SchnaubeltFrancoise MartzSebastian SpringerFrançoise PorteuSebastian TancoFrançois-Xavier LegrandSebastian WerneburgFrane PaicSebastiano CiccoFrank BernhardSebastiano RecalcatiFrank BraunSebastiano TorrisiFrank KoSébastien GouardFrank KrauseSébastien J. DumasFrank KruytSebastien JacquelinFrank OechslinSébastien PenninckxFrank PaulsenSébastien Sébastien WieckowskiFrank PetersenSebastien VielFrank PfriegerSebastjan BevcFrank Richard SchubertSebnem Ece EksiFrank SchnütgenSeda KoyuncuFrank SuhrSeddik HammadFrank TaraziSeema GuptaFrank Van BelSeethalakshmi HariharanFrank ZauckeSeetharama D. S. JoisFranka Klatte-SchulzSefan AsamitsuFranklin NobregaSeh-Hoon OhFranklin W.-N. ChowSe-Hwa KimFranklin Wang-ngai ChowSeigo SanohFrans CremersSeiichi IshidaFrantisek BaluskaSeiichi KitagawaFrantišek KrčmaSeiji FukudaFrantišek ŠtaudSeisuke AtaFranz JakobSeisuke KimuraFranz L. DickertSekar VijayakumarFranz RoedelSekyoo JeongFranz WeberSelcuk KucukseymenFranziska DenglerSelma MlinarićFraser CollinsSelvakumar EdwardrajaFreddy MoraSemih Can AkıncılarFrederic BecqSenaka RanadheeraFrédéric CapelSenem KamilogluFrédéric CoutantSenol PiskinFrédéric DumurSenthilnathan PalaniyandiFrédéric EbsteinSeog-Young YoonFrédéric G. BrunetSeon Hwa KimFrederic LezotSeong Kyu HanFrederic MarsolaisSeong Soo A. AnFrédéric Nicolas DaussinSeong Who KimFrédéric SaltelSeong Young KwonFrédéric VelardSeong-Gon KimFrederic W. HopfSeonyeong KwakFrederick J. FullerSeon-Yong JeongFrederik DenormeSepanta HosseinpourFrédérique A. O. HilliouSe-Ran YangFrederique PeronnetSerdar DursunFrederique VerdierSerena BianchiFredrik EdforsSerena BoninFredrik SchaufelbergerSerena CecchettiFreeborn RwereSerena CoiaiFriederike KlempinSerena DuchiFriederike TrognitzSerena StangaFriederike ZunkeSerena VeschiFriedrich AltmannSerge LyashchenkoFriedrich JungSerge MuyldermansFuad IraqiSerge NatafFuat TopuzSerge PerezFuhua HaoSergei Andreevich DzubaFulvia FarabegoliSergei G. GaidinFulvia OrtolaniSergei NechaevFulvio BorellaSergei RyazanskyFulvio CelsiSergei SpiridonovFulvio LauretaniSergei Y. GrishinFulya TaylanSergej PirkmajerFumiko SekiguchiSergey A. AkimovFumio ChikamoriSergey A. ShmakovFumio SakaneSergey A. SinenkoFumitaka KogaSergey BaykovFun-In WangSergey BelyakovFurong TianSergey BrezginFuyang LiSergey ChetverikovFuyou FuSergey DenisovFuyuki SatoSergey DikalovG. Andres ContrerasSergey KolomeǐchukG. BrajuškovićSergey KozlovGábor KocsySergey LashinGábor KökénySergey LevitskiiGábor KonczSergey M. IvanovGábor LendvaiSergey MalkinGábor MátisSergey N. LominGábor RigóSergey SamsonovGábor TuruSergey SedykhGábor VargaSergey ShekhovtsovGabriel EstrelaSergey ShvetsovGabriela AmbrožováSergey SiletskyGabriela Loredana PopaSergey V. DorozhkinGabriele TabaracciSergey V. OkovityiGabriele TumminelloSergi ClotetGabriella EndreSergio CominciniGabriella SarmaySergio CortelazzoGabriella TamasiSergio Crespo-GarciaGaetano MarvertiSergio DavinelliGaewon NamSergio EspositoGaia FaustiniSérgio F. SousaGalina PavlovaSérgio Filipe SousaGalina RalduginaSergio FucileGangjee KoSergio Granados-PrincipalGannon C. K. MakSergio M. Granados-PrincipalGaochao DongSergio Martínez-HøyerGary SteinmanSergio Martínez-RodríguezGary StephensSergio Miguel Castaneda ZegarraGaurav JoshiSergio MinucciGauravi DeshpandeSergio MorenoGautam SethiSergio OchattGaynor Ann SmithSérgio P. C. AlvesGenevieve C. SparagnaSérgio P. CamõesGenjiro SuzukiSergio Rius-PérezGeoffrey BrownSergiu FendrihanGeoffrey HartSergiusz LulińskiGeoffrey I. SandleSeri JeongGeorg FeichtingerSerkan YílmazGeorg FuellenSeth FrietzeGeorg VargaSeung Hee LeeGeorge F. RohrmannSeung Hwan LeeGeorge LambrinidisSeung Hyun KimGeorge P. StudzinskiSeungkwon YouGeorge PadosSeung-Soon ImGeorge PotamiasSeungwoo KangGeorge Z. KyzasSeung-Yup KuGeorgeta Violeta TruscaSevda ŞenelGeorgiana CodinaSeverin MairingerGeorgios AndroutsopoulosSéverine BärGeorgios BokiasSeverine Chaumont DubelGeorgios ParaskevopoulosSeverino PersechinoGeorgios SpyrouliasSevero Vázquez-PrietoGeorgios TsaniklidisSeyed Khosrow TayebatiGeorgios TsiavaliarisSeyedAbdolreza SadjadiGeorgios TziatziosSeyun KimGera SoniaSeyyed Mojtaba MousaviGerald F. AudetteSezai ErcisliGerald Fritz SchusserSha ShaGerald TomkinShad B. SmithGerard MartensShadab MdGerarda CappuccioShafiul AlamGergely FeherShahab UddinGergő KallóShahabaldin RezaniaGerhard StegerShahid FarooqGerry LushingtonShahid KhanGertjan KramerShahid MansuriGeun-Shik LeeShahin HomaeigoharGeza BujdosoShahrokh GhobadlooGhais KharmandaShahrul SarbiniGheorghe IliaShahzad MunirGholamali RahnavardShailabh KumarGhulam Md AshrafShailendra Pratap SinghGiada AmodioShailesh K. ShahiGiampiero CaiShailesh KumarGiampiero MeiShakti SinghGianCarlo PecorariShakur MohibiGianluigi MazzoccoliShamroop Kumar MallelaGiin-Yu Amy TanShan YanGilbert ErianiShancy Petsel JacobGil-Martínez MartaShane LiddelowGimeno MercedesShang-Te Danny HsuGiorgia AilunoShangting YouGiorgio ColomboShankar Jaikishan EvaniGiorgio GambinoShankung LinGiorgio RispoliShannon C. KenneyGiorgio SantoroShannon L. KelleherGiovanna LattanziShantanu PradhanGiovanni BarillariShanthi G. ParkarGiovanni C. ActisShan-Ying WuGiovanni CannarozzoShao ChunlinGiovanni DamianiShao Jun TangGiovanni Di ZenzoShaobin WangGiovanni TarantinoShao-Chen LeeGitta TurowskiShaofei JiGiulia AbateShao-Hsuan KaoGiulia BerzeroShaolei TengGiulia CasoratiShaopeng PeiGiulia MatacchioneShaoqiu HeGiuseppe AmorusoShao-Wen HungGiuseppe BiamontiShaoyong LuGiuseppe De PalmaSharan BobbalaGiuseppe GattusoSharan SwarupGiuseppe JurmanShariq QayyumGiuseppe Lucio CasciniSharma SachinGiuseppe MalfaSharmila FagooneeGiuseppe MurdacaSharmila MasliGiuseppe PalumboSharmishtha MusalgaonkarGiuseppe PortellaSharon K. MichelhaughGiuseppe QuerquesSharon SwangerGiuseppe SammarcoShashank PandeyGiuseppe SantarpinoShashank SrivastavaGiuseppina MartellaShashi Shekhar SinghGiusto TrevisanShatavisha DasguptaGiusy TornilloShawn ChristensenGizak AgnieszkaShawna L. McMillinGlatz KatharinaShazid Md. SharkerGlen BorchertShebli AtrashGlenn LoboShefali DobhalGloria Benítez-KingSheikh RiazuddinGloryn ChiaShekhar SahaGo KanzakiSheldon LinGobinda SarkarShelly KaushikGoedele MaertensShelly LuGottfried J. PalmSheng-Fan WangGottfried UndenSheng-Mou HsiaoGötz HenselSheng-Nan WuGovinal Badiger BhaskaraSheng-Wei FengGraça LopesShengwen Calvin LiGrace Y. SunShenkui LiuGrażyna DobrowolskaSheo PandeyGregory CaputoShereen Cynthia D′CruzGroo Anne-ClaireSherif EbadaGrzegorz BulajSherif T. S. HassanGrzegorz PanasiewiczSherry ShuGrzegorz R. JuszczakSheyda ShakibaGrzegorz SchroederShian-Ren LinGrzegorz ZagułaShiao-Wen TsaiGuanglei ZuoShichen ShenGuangzhao LiShidan WangGuan-Ting PanShien-Der TzengGuglielmina ChimientiShifra Ben-DorGu-Jiun LinShigeaki SunadaGulam RabbaniShigefumi OkamotoGunn-Guang LiouShigeharu UekiGünter EmonsShigehisa AokiGünther SchererShigeki FuruyaGuofang ShenShigeki MatsubaraGuohua HuaShigeki SetoGuozheng WangShigeki SugawaraGurpreet Kaur AroraShigemi SeoGurtej DhootShigeo OhbaGururaj Kudur JayaprakashShigeo S. SuganoGustavo Di LalloShigeru MatsumuraGuy RostokerShigeru MorimuraGuzel R. KudoyarovaShigetaka ShimodairaGwang Hyeon EomShigeto MoritaGwendolyn Vaughn ChildsShigetoshi SanoGyörgy BallaShigeyuki TanakaGyörgy DormánShih-Chao LinGyörgy VámosiShih-Che SueGyőző SzolnokyShih-Chieh ChenGyudo LeeShih-Feng ChoGyula BattaShih-Heng ChenH. Dayton WildeShih-Hsun HungH. Denis AlexanderShih-Hua LinH. M. M. Abdel-AzizShih-Kuo ChenHae-Rahn BaeShih-Min HsiaHaewon ByeonShiho NaitoHafiz AhmedShiho OhnishiHahn Young KimShihori TanabeHai-Chou ChangShih-Sung ChuangHaifei ShiShihui JiaoHaigh TonyShih-Yen LoHailin WangShih-Yi HuangHaining YangShih-Yi LinHaishu LinShi-Jye ChuHaixiao HuShike WangHajime KonoShilpa IyerHak Soo SeoShilpa SinghHakim EchchannaouiShilpa ThakurHalil İbrahim ÇiftçiShimeles TilahunHamid BadaliShimi MeleppattuHamid MaadiShimpei HigoHamza El HadiShin EnosawaHana BuchtováShin Ichi ArimuraHandan XiangShin La ShuHang Nga MaiShin TaketaHang QiShing Yau TamHang Soo ParkShing-Hwa LiuHang ZhouShingo KasamatsuHanha ChaiShingo NakahataHanki KimShin-ichi FujiwaraHanki LeeShin-ichi HorikeHankyu LeeShin-ichi NakamuraHan-Mou TsaiShinichi SatoHanna BandurskaShinichi SuzukiHanna BurkhalterShin-Ichi TateHanna Dams-KozlowskaShin-ichiro KarakiHanna DanielewiczShinichiro KashiwagiHanna KmitaShinichiro MorichiHanna MannellShining LooHanna MoniuszkoShinji AsanoHanna SchilbertShinji KajimotoHannah K. DrescherShinji MiuraHann-Chorng KuoShinji NagataHannes GlaßShinji TakaiHannu KoistinenShinji TakamatsuHans BinderShinobu OhnumaHans BockShintaro SukegawaHans H. HendriksShinya HasegawaHans Henning Von HorstenShinya KanzakiHans Peter DeignerShinya TakahashiHans Peter DienesShiou-Hwa JeeHans Van LeeuwenShiqian ShenHans-Christian SiebertShiri LiHans-Georg ClassenShiro FukutaHans-Georg SchaibleShiro KoizumeHans-Jörg MeiselShiro MitsuyaHans-Richard SliwkaShiro SuetsuguHanuma Reddy TiyyaguraShisong RongHanwei JiaoShiu Cheung LungHany I. SakrShiv BharadwajHany MareiShiv VermaHanying ChenShiva AkbarzadehHanyong PengShiva GadilaHan-Yu HsuehShiva KantHanzhong KeShiva PathakHao HuangShivanand M. PudakalakattiHao YuanShivshankar ThanigaimaniHao ZuoShiwei ZhuHao-ai ShuiShizu Hirata-TsuchiyaHaobo LiShizuka UchidaHao-Ching WangSho NishidaHao-Jui WengShobhan GaddameedhiHaoming ZhangShofiul AzamHaoshen ShiShogo OzawaHao-Xun ChangShogo ShigetaHapuarachchige Chanditha HapuarachchiShohei MitaniHaq Abdul ShaikShoko Morita-TakemuraHaralambos E. KaterinopoulosShokrollah ElahiHarald LahmShona MookerjeeHarald PlattaShona MurphyHarald W. PlattaShosuke ItoHarant HannaShota YamamotoHardik AminShovan NaskarHardik KundariyaShow-Mei ChuangHari Datta KhanalShozo YanoHaridha ShivramShravan GiradaHariprasada R. Kanna ReddyShravan MorlaHaris KamalShrestha Sinha RayHarish HandralShrey KohliHarish Palleti JanardhanShrikant PawarHarish VashisthShrikant SharmaHarleen KaurShriram VenkatesanHarri LönnbergShruti ChoudharyHarris PratsinisShuai GaoHarry Karmouty-QuintanaShuai WangHarry ScherthanShuang ZhangHartmut KuhnShuanhu ZhouHaruhiko JimboShubhankar NathHaruhiko KashiwazakiShuchi WangHaruhiko SugimuraShu-Chun ChangHarukazu SuzukiShugo MaekawaHaruki KoikeShuhei YamadaHaruo NakayamaShuichi MatsumuraHaruyoshi YamazaShuichi SakamotoHasan AldewachiShuichi ShigenoHasan MehrajShuji OginoHaseeb ZubairShuji OzakiHasin FerozShuji TachibanakiHassan Abdoul-CarimeShuji YokoiHassan El-RamadyShu-Ju WuHassan KassassirShu-Jui KuoHassen S. WolleboShu-Ling TzengHatasu KobayashiShumin WangHatem A. ElshabrawyShun IshibashiHatem ElshabrawyShun MaekawaHauke BuschShun-Fen TzengHåvard JenssenShungo OtagakiHaw-Ming HuangShunji ItoHaya Lorberboum-GalskiShunmoogum A. PattenHayato MaedaShunsuke WatanabeHazem S. ElshafieShunyi JianHe LiShuowei CaiHeath MurrayShu-Pin HuangHeather C. EtcheversShusei KanieHeather DrummondShuzo SatoHeather EnrightShweta SharmaHeather WilkinsShwu-Chen TsayHeba AbdelrazikShyam Kumar GudeyHeba EbeedShyam MoreHéctor CarrascoShyamalagauri JadhavHector De Paz CarmonaShyh-Chyi LoHector HerreraShyi-Long LeeHector Mora-MontesShyiwu WangHedwig Sutterlüty-Fall HedwigSi ChenHee Jung KimSi Hyung LeeHee Kyoung JooSiang Boon KohHee Min YooSibali DebnathHee Y. KangSibhghatulla ShaikhHee Young KangSi-Chong ChenHeesun CheongSiddharth ShuklaHeeyoung ChaeSidney J. HayesHeicko LemckeSiegfried LabeitHeidi NeubauerSiemowit MuszyńskiHeidi TillerSierra A. ColavitoHeike EndepolsSigmar StrickerHeike KroegerSigrun EickHeike WeighardtSijin GuoHeinrich StichtSijo MathewHei-sung KimSijung YunHeithem Ben AmaraSilvan KleinHelder AndreSilvana AlfeiHelen ForresterSilvana BriugliaHelen HughesSilvana GaetaniHelen KnowlesSilvana MorelloHelen MirandaSilvana ZanlungoHelen MottSilvano FerriniHelen ParfreySilverio CocoHelen StolpSilverio RotondiHelen V. NewSilvia AlbertHelena Cristina VasconcelosSilvia Alberti-ViolettiHelena FelgueirasSilvia Alice LocarnoHelena Jernberg-WiklundSilvia Ayora HirschHelena PortaSilvia BarbonHelena RibeiroSilvia BarrabésHelena RitchieSilvia BattistoniHelena ŠtorchováSilvia BistiHelena TrindadeSilvia BuroniHélène Dubois-Pot-SchneiderSilvia CañasHelene FachimSilvia CaponiHélène RoumesSílvia Castro CoelhoHelga KraxnerSilvia CorrochanoHelga MoncayoSilvia DeaglioHelge Immo LehmannSilvia Di AgostinoHélia CardosoSilvia DottiHélio AlbuquerqueSilvia DragoniHelle DamkierSilvia FalcinelliHelle Smidt MogensenSilvia FischerHelmut BerglerSilvia FranchiHelmut GreimSilvia GramolelliHelmut MairSilvia Guil-LunaHelmy M. YoussefSílvia J. BidarraHemant GiriSilvia LandiHemant K. MishraSilvia LiskovaHemantkumar ChavanSilvia LiuHemendra VekariaSilvia M. Sanz-GonzálezHemmen SabirSilvia MarchianòHengyou ZhangSilvia MarracciHenk FranssenSilvia MeziHenk KoppelaarSilvia PampanaHenna MyllymäkiSilvia PasquiniHenning ArltSilvia PomellaHenning PoppSilvia RiondinoHenriette Elisabeth AutzenSilvia RocchiccioliHenrik PedersenSilvia RussoHenrike LucasSilvia S. BarbieriHenrique GiraoSilvia SalvatoreHenry Chun Hin LawSilvia SocorroHenry J. ThompsonSilvia Stefania LongoniHenry P. GodfreySilvia SvegliatiHenry PleassSilvia ValtortaHenry SutantoSilvia VasiliuHenry WongSílvia Vilares CondeHenryk KozlowskiSilvia VilasiHerbert R. TreutleinSilvia ZappavignaHerbert Ryan MariniSilvia ZorrillaHerbert SchneckenburgerSilvie BernatováHerbert SchulzSilvina LompardíaHeriberto Rodriguez MartinezSilvio MaringhiniHerjan J. T. Coelingh BenninkSilvio NaviglioHerman SintimSilvio TundoHermis IatrouSim NamkoongHermona SoreqSimerdeep Kaur DhillonHernan Diego FolcoSimon AmiardHerui WangSimon ChuHervé BègueSimon D. Van HarenHervé Le StunffSimon E. LewisHervé QuiquampoixSimon G. PatchingHervé SchohnSimon Kwoon-Ho ChowHesham El-SeediSimon L. GoodmanHesso FarhanSimon LebaronHeta MattilaSimon McArthurHeung-Man LeeSimon Petersen-JonesHiba NatshehSimon R. LawHicham M. DrissiSimon SieberHicham ZegzoutiSimona BernardiHidayat HussainSimona BiancoHideaki FujiiSimona BolampertiHideaki IjichiSimona BungauHideaki InagakiSimona CabibHideaki KanetoSimona CandianiHideaki OkajimaSimona Carmen LiţescuHidehiko KikuchiSimona CeccarelliHidehiro OkuSimona CitroHidekazu HiroakiSimona D′AguannoHidekazu TakahashiSimona De SummaHideki AiharaSimona DedoniHideki FujiiSimona FermaniHideki KitauraSimona GurzuHideki MochizukiSimona LucioliHideki SakaiSimona MagiHideki ShibataSimona MartinottiHideki SugiiSimona NardozzaHideki TakaseSimona NicoaraHideki WanibuchiSimona OlmiHideko SoneSimona PagliucaHidemasa BonoSimona PergolizziHidemasa IzumiyaSimona SacchiniHidenori NishiharaSimona SerratìHideo HashizumeSimona TrittoHideo IwaïSimona ViglioHideo TsukadaSimona ZaamiHideo WadaSimonas RamanaviciusHideto TsujiSimone BattagliaHidetoshi KassaiSimone BeninatiHidetoshi KomatsuSimone BrogiHideyuki J. MajimaSimone CantamessaHieronim GolczykSimone DonatiHieronim JakubowskiSimone GrassiHilda E. GhadiehSimone MirabiliiHilde Harb BuzzáSimone MorselliHilka Rauert-WunderlichSimone PaliniHimangshu SonowalSimone PompeiHimanshu KathuriaSimone SimoniHinanit KoltaiSimone TambaroHindra HindraSimone VanoniHira FatimaSimons SvirskisHiraku OnoSimran MaggoHiran A. PragSina NaserianHirase HajimeSina ShadfarHirayuki EnomotoSinead M. SmithHiroaki FujiiSinem KaramanHiroaki KodamaSinisa BjelicHiroaki KonishiSinu P. JohnHiroaki NakagawaSipei LiHiroaki SaikaSiqi ChenHirofumi HiokiSiranjeevi NagarajHirofumi KuritaSiri PauloHirohiko TaniSiro LuvisettoHirohito IchiiSiro SimizuHirohito YanoSirpa JalkanenHirokazu FukuiSitanshu S. SinghHirokazu KimuraSitthivut CharoensutthivarakulHirokazu SakamotoSittisak HonsawekHiroki KokuboSiva Krishna AngalakurthiHiroki MitomaSiva S. PandaHiroki MizukamiSivakumar Vadivel GnanasundramHiroki TeragawaSivaraj Mohana SundaramHiroki ToyodaSivaraman NatarajanHiroki YokotaSiyam M. AnsarHiroko Ikeshima-KataokaSiyi GuHiroko YamashitaSjoerd RijpkemaHiromi SakaiSkalicka-Woźniak KrystynaHiromi SatoSlađan PavlovićHiromi YanagisawaSlaven CrnkovicHiromichi SakaiSławomir BorekHiromitsu NegoroSlawomir CiesielskiHironori YoshinoSlawomir GonkowskiHiroo ImaiSlawomir GrabowskiHiroshi FujisakiSławomir LewickiHiroshi FukushimaSławomir MichałekHiroshi HibinoSławomir MilewskiHiroshi KikukawaSlawomir TeperHiroshi MitomaSławomir WołczyńskiHiroshi MiyamotoSlobodan VukicevicHiroshi NagashimaSmijin K. SomanHiroshi SaitoSmilja TodorovicHiroshi SakaueSmrithi PadmakumarHiroshi ShimizuSmruti ChaudhariHiroshi TakemoriSneha RajuHiroshi TomodaSneha VivekanandhanHiroshi TsutsumiSnežana D. SavićHiroshi WakoSnezana Ilic-StojanovicHiroshi YoshidaSnezana KusljicHiroshi YoshitakeSnježana KerešaHirotada MoriSnježana Mardešić-BrakusHirotaka ImaiSnježana RamićHirotaka MatsuiSnjezana Zidovec-LepejHirotake TsukamotoSo Hee KwonHirotoshi EchizenSobia ZaidiHirotsugu OhkuboSobuj MiaHiroyasu SakaiSodio C. N. HsuHiroyoshi MoriSofia Barbosa-GouveiaHiroyoshi Y. TanakaSofia Ferreira GonzalezHiroyuki AonoSofia FranciaHiroyuki KanzakiSofia GamaHiroyuki KurataSofia GuedesHiroyuki NakamuraSofia KarkampounaHiroyuki TakamaruSofia LimaHiroyuki TsuchiyaSofia M. MorozovaHiroyuki WakiguchiSofia PereiraHiroyuki YamadaSofia ShevtsovHisako MasudaSofia SoaresHisanori FukunagaSofiane Y. MersaouiHisashi HaradaSofie V. NielsenHisashi IzasaSohel M. JuloviHisashi MeraSohsuke YamadaHisato YagiSoichiro YamamuraHisayuki KomakiSoisungwan SatarugHisham BahmadSokhna M. S. Yakhine-DiopHisham FansaSokou RozertaHiu-yee KwanSoleiman Mohammadi SamaniHo Bang KimSoliman KhatibHo Leung NgSolomon AfelikHo LinSolomon MainaHo TsoiSomchai AmornyotinHo Won JungSomchai ChutipongtanateHoang Khoa LySon Long HoHojin RyuSona BalentovaHolger BierhoffSonal ShreeHolger EltzschigSonali NashineHolger ErbSonam KumariHolger FlechsigSondra T. BlandHolger Jon MøllerSong Cheol KimHolger PuchtaSongbo WangHolger WilleSonghai TianHollis E. KrugSongyoung ParkHolly Bauser-HeatonSonia AroniHoman KangSonia BañuelosHomero Reyes De La CruzSonia BruñaHong JinSónia C. CorreiaHong Kwan KimSonia CacciaHong MaSónia CarabineiroHong Sheng ChengSonia De CastroHongbo JiangSonia Eiras PenasHong-Duck KimSonia EliginiHonghe LiuSonia Escandón-RiveraHong-Ki LeeSónia G. PatrícioHongliang HeSonia GonzálezHong-Long JiSonia L. AlbarracínHongnan CaoSonia LeviHong-Seob SoSonia M. OliveiraHongshun YangSonia MartinsHong-Wa YungSonia NathHongwei HanSonia RamosHongxia RenSònia SeguraHongyu QiuSonia SernaHong-Yuan HuangSonia SeverinHoon KimSonia Torres-SanchezHoria HaragusSonia ZuñigaHorst VogelSonja HinzHorst Von BernuthSonja KollersHosam O. ElansarySoo In LeeHossam AshourSoo Jin YangHossam El-Sheikh AliSoochong KimHossein AhangariSooim ShinHossein HosseinkhaniSoo-Kyoung ChoiHossein TabatabaeianSoon Ae KimHossein TaghizadehSoon Hee KimHouda BenhelliSoon KimHouria DaimiSoon Wook KwonHouwen PanSoon Yew TangHoward S. YoungSoong Deok LeeHowbeer Muhamad AliSoonwook KwonHridaya ShresthaSoo-Youl KimHristo PenchevSophia HoberHrvoje JakovacSophia LetsiouHrvoje RimacSophia X. SuiHsi ChangSophie JarraudHsiangen WuSophie Quevillon-CheruelHsiao Tien LiuSophie ShawHsiaohang ChungSoraya L. VallesHsiao-Hsuan KuoSoraya Paz MontelongoHsien-Bin HuangSören LukassenHsien-Ming LeeSoriful IslamHsien-Yuan LaneSorin HostiucHsih-Yin TanSorin T. BarbuHsin Ling HsuSotirios G. StavropoulosHsin-Chen LeeSotirios G. ZarogiannisHsin-Chien ChenSotirios KatsamakasHsing-Chun LinSotiris AmillisHsin-Yao WangSotiris K. HadjikakouHsiu-Chen HuangSotiris KotsiantisHsiyuan HuangSouichi SuenobuHsueh-Te LeeSoulaiman SakrHsueh-Wei ChangSoumita GhoshHsu-Feng LuSoumya IyerHsu-Wen ChaoSoumya PoddarHu ChenSoumyadev SarkarHu QiaobinSoumyajit D. RoyHu WangSoumyashree GangopadhyayHuabin CaoSousa JavanNikkhahHuafeng WangSouska ZandiHuaishan WangSousuke ImamuraHuan ZhangSovann KhanHuanyu (Larry) ChengSowmya R. RamachandranHuapeng FengSowmyalakshmi SubramanianHuarong ChenSoyeon LimHua-Tao WuSoyoun KimHubert SytykiewiczSo-Youn WooHubert ZiółkowskiŠpela KonjarHudson Caetano PoloniniSperanza RubattuHue Jung ParkSpiridon MantzoukasHuei-Jane LeeSpiro M. KonstantinovHueng-Chuen FanSpundana MallaHugo Arasanz EstebanSpyridon KintziosHugo Arias-PulidoSpyridon PetropoulosHugo MartinianoSpyros PerlepesHugo MirandaSpyros TsiftsisHugo Vara RiveraSrba MijailovichHugues Allard-ChamardSrecko GajovicHugues J. ChapSrecko StopicHugues JacobsSree Deepthi MuthukrishnanHui HuangSreedhara SangadalaHui JingSreeHarsha NagarajaHui LiuSreejith RaveendranHui LuSreekumar OthumpangatHui WangSreelakshmi VasudevanHui Yin LimSreenivasulu ChintalaHuibin LvSreenivasulu KilariHui-Chen ChenSreeprasad T. SreenivasanHui-Chun WangSri Renukadevi BalusamyHui-Hua HsiaoSridar ChitturHuiming LuSridhar MuthusamiHuitao LiuSrikanth GattuHuiying ZhangSrikanth MandaHuize PanSrinivas AbbinaHuizhi WangSrinivas AluriHumberto HernandezSrinivas Niranj ChandrasekaranHumira SonahSrinivas SistlaHun Sik KimSriramana KanginakudruHung Huey-ShanSruthy Maria AugustineHung-Chi ChengSsu-Wei HsuHung-Chuan PanStacey SchutteHung-Pin TuStanescu Ana Maria AlexandraHung-Wei YangStanislav KafkaHung-Yao HoStanislav M. CherepanovHung-Yi ChuangStanislav MiertusHung-Yi WuStanislav PantelyushinHung-Yin LinStanislav PshenichnyukHussain Yar KhanStanislav RybtsovHussein KaddourStanislav S. TerekhovHussein N. RubaiyStanislav TrdanHwa-Jin LeeStanislava PankratovaHwa-Jung KimStanisław KrompiecHwangjae LeeStanisław NiemczykHye Jin ShinStanislaw OłdziejHye Jin YouStanislaw SłomkowskiHye Kwon KimStanisław WitkowskiHye One KimStanislawa Bazan-SochaHye Ryoun KimStanley LipkowitzHye Young KimStanley StevensHyemin KimStathis FrillingosHyeong Sim ChoiSteen J. MadsenHyeong-Geug KimSteet RichardHye-ra LeeStefaan VerbruggenHyo-Jong LeeStefan BauersachsHyonchol JangStefan BergerHyotcherl IheeStefan BoehmHyouta HimenoStefan BroerHyuck Jin LeeStefan BurénHyuk-Kwon KwonStefan Cristian VesaHyuk-Sang JungStefan DüsterhöftHyun Jin KwunStefan HeinrichHyun Joo AnStefan Ioan VoicuHyun Koo KimStefan LauferHyun Woo ChoiStefan MagezHyunbo ShimStefan Marian FrentHyung Joo KwonStefan NaydenovHyung Joo SuhStefan PuschHyungjin KimStefan RiwaldtHyungjong ParkStefan SchildknechtHyungju KwonStefan SchwanHyung-Sik KimStefan TanglHyungsuk KimStefan TimmHyunjoon ParkStefan W. VetterHyunju RoStefan WegerHyunsoo KimStefania BroccaHyun-sook LeeStefania CantoreHyun-sun ParkStefania CaparrottaHyun-Woo ParkStefania CastagnettiHyun-Woo RheeStefania CerutiHyun-Woo ShinStefania CoccoI. G. TiganovaStefania ContiI. P. GrigorievStefania D′AngeloIain HargreavesStefania GaldieroIain LamontStefania LamponiIain M. MorganStefania MarianoIain MacArthurStefania MarsiliIain Parry HargreavesStefania MarzoccoIan Charles PatersonStefania MerighiIan CowellStefania MocciaIan E. GentleStefania MomiIan JohnsonStefania PilatiIan KerrStefania PragliolaIan M. RogersStefania RacovitaIan MacDonaldStefania RaimondoIan RossStefania SciallaIan SaltStefania StoleriuIan TetlowStefania SutIan WymanStefania VillaIbolya AndrásStefanie GierIbrahim BitarStefanie Kennon-McGillIbrahim M. SayedStefanie KleinI-Cha LinStefanie MaurerI-Chen PengStefano AccoroniI-Chen WuStefano BellostaI-Chi LeeStefano BellucciIchiro HirataniStefano BeniniIchiro HisakiStefano BiffoIchiro TakadaStefano CacciatoreIda CariatiStefano CagninIda Daniela PerrottaStefano CampanerIda MannaStefano CanosaIddo PinkasStefano CardeaIdris RajiStefano CiciliotIgea D′AgnanoStefano CorniIghli Di BariStefano CrespiIgnacija VlašićStefano D′AlessandroIgnacio DávilaStefano Da PozzoIgnacio Díez LópezStefano DastoliIgnacio Novo-VeleiroStefano DocciniIgnacio RodríguezStefano FaisIgnacio Torres AlemanStefano Farioli-VecchioliIgnazio Gaspare VetranoStefano FocaroliIgor A. KhalymbadzhaStefano GarofaloIgor A. PaštiStefano GastaldelloIgor B. RogozinStefano GottiIgor BelyaevStefano GrolliIgor Borisowitsch BuchwalowStefano LeporattiIgor BuravlevStefano MarulloIgor DeynekoStefano P. PiottoIgor E. DeyevStefano PiazzaIgor E. GolubStefano PierettiIgor G. KhaliulinStefano Stefano NobileIgor I. KireevStefano TozzaIgor Iuco Castro Da SilvaStefano TuroloIgor IvanovStefano VaggeIgor JakovcevskiStefanos LeontopoulosIgor JerkovićStefanos RoumeliotisIgor JermanSteffen DaubIgor JordanovSteffen JustIgor JurakSteffen OrmannsIgor KukhtevichSteffen VannesteIgor MaiborodinSteffen WitzlebenIgor MaksimovStelian Sergiu MaierIgor Moiseyevich KvetnoyStelios PsarrasIgor OscorbinStella ArelakiIgor Piotr TurkiewiczStella LeeIgor PottosinStepczynska MagdalenaIgor PrudovskyStephan DreschersIgor RouzineStephan FischerIgor SeršaStephan ImmenschuhIgor ShmarakovStephan Joel ReshkinIgor SplichalStephan KrähenbühlIgor TomasevicStephan LindemannIgor ZhukovStephan NieuwoudtIhn HanStephan PhilippIhor S. VirtStephan ReshkinI-Ju YehStephan SchillingI-Jung TsaiStephan Van EedenIke De La PeñaStephan Von HörstenIke OlivottoStephana CarelliIk-Hwan KimStéphane BurteyIkuo InoueStéphane ChavanasIkuo TsunodaStephane EsnautIl Soo MoonStephane LemiereIlaria BaglivoStephane R. GrossIlaria BellezzaStephane RetyIlaria CeccarelliStéphane SavaryIlaria CosciStéphane TéletchéaIlaria DecimoStéphane TerryIlaria DettoriStephanie BaudIlaria Elena Lena PalamàStephanie BurrIlaria FerrarottiStephanie ByrumIlaria FrassonStephanie C. JoachimIlaria GiustiStéphanie CabantousIlaria IacobucciStephanie DauthIlaria OttonelliStéphanie DavalIlaria ParentiStephanie DuguezIlaria PianoStephanie Evelyn KingIlaria ReaStephanie F. LingIlaria RubinoStephanie HallIlaria RussoStephanie HehlgansIlaria TamagnoStephanie JoachimIlavenil SoundharrajanStephanie KreisIldikó BácskayStephanie L. OsbornIldikó CsókaStephanie LongetIldikó DomonkosStéphanie PlenchetteIldikó JócsákStephanie SwarbreckIldiko RaczStéphanie ToméIldiko SzantoStephen A. MorrisIleana Alexandra PavelStephen B. CarrIleana C. FarcasanuStephen C. HardiesIleana ManduteanuStephen CessnaIleana RauStephen ChimIlhem MessaoudiStephen D. BarrIlian BadjakovStephen FairweatherIlias DoulamisStephen HillIlias ElmoukiStephen InbrajIlias MigdalisStephen J. BushIlias P. NikasStephen J. PetersonIlie BodaleStephen J. TerryIlijana GrigorovStephen K. RandallIlijas JelcicStephen LewisIliya PetrievStephen MartinIliyan IvanovStephen O. DukeIlja L. KruglikovStephen O. EvansIlka EngelmannStephen SawcerIlker BayerStephen TooveyIlkeun LeeStephen TsoiIlkka K. IlonenStephen WilsonIlkka M. KantolaStephen YarwoodIlona KovalszkyStergios BoussiosIlona RybińskaStergios KatsiougiannisIlona SadokSteve EllisIlya A. OstermanSteve FiesterIlya G. ShenderovichSteve MurrayIlya O. VelegzhaninovSteve N. FieringIlya UlasovSteve WiltonIlya V. KorolkovSteven A. FreseIlya YakavetsSteven B. MarstonIlze ŠtrumfaSteven F. GameiroIman KavianiniaSteven FeldmanIman ManavitehraniSteven H. StraussIman MirmazloumSteven HeadImelda Ontoria-OviedoSteven HollowayI-Ming ChuSteven J. BurgessImmacolata AndolfoSteven LevyImmacolata SerraSteven M. FrischImo HoeferSteven Michael ClaypoolImola WilhelmSteven N. FieringImourana Alassane-KpembiSteven Paul NisticòImran FarooqSteven R. DuncanImran HouseSteven RothImran KazmiSteven T. DenhamImran UllahSteven ThrelkeldImre HolbSteven VermeulenImre LengyelStevo NajmanImre SóvágóStig LinderImtiaz KhanStojanovska VioletaIn Jung KimStormi Pulver WhiteIn Koo HwangStoyan PetkovIna KochStrzemski MaciejIna TarkkaStuart AstburyInaam NakchbandiStyliani GizaIñaki EtxeberriaStyliani N. ChorianopoulouIñaki Milton-LaskibarStylianos PouliosIn-Beom KimStylianos RavanidisInbo HanSu Young KimInchan KwonSuba RajendrenIndira ShrivastavaSubban KathiravanIndra ChandrasekarSubbroto Kumar SahaIndra D. SahuSubendu SarkarIndrajit ChowdhurySubhadeep ChakrabartiIndu G. RajapakshaSubhan DanishIndu KhatriSubhashree SubramanyamIneke JansenSubhasish ChatterjeeInes Bilic-ĆurčićSubodh VermaInês BrandãoSubramani PandianInês ChavesSubrata DebInês Dinis AiresSubroto ChatterjeeInês DinizSuchithra Ashoka SahadevanInes DrenjančevićSuchitra KamleInes LiebscherŠuchová KatarínaInês M. AraújoSudarson Sekhar SinhaInes PrimožičSudeep TiwariInês SilvaSudha SharmaInês SimõesSudhanshu AbhishekInes SwobodaSudhanshu RaikwarInes Vieira Da SilvaSudhir PandeyInese CakstinaSudhir Putty ReddyInese FilipovaSudip BanerjeeInga KwiecienSudip MukherjeeInga NeumannSudip PaulIng-Ming ChiuSudipto RoyIngo NolteSue C. LeeIngo SchmitzSuet Nee ChenIngrid Brust-MascherSugantha P. ElayapillaiIngrid CipakovaSugasini DhavamaniIngrid DijkgraafSugato HajraIngrid LangerSuhail RazakIngrid Lang-OlipSuhas ShindeIngrid MollSuhrid BanskotaIngrid PlottonSu-Hyung HongIngrid Rosenburg CordeiroSu-Jane WangIngrid TonhajzerovaSujata MohantyIn-Hong ShinSujay GuhaIn-Jung KimSujay RayInka BrockhausenSu-Ji JeonInkyu ParkSujit BasakInkyung JungSuk Ling MaInmaculada BanegasSukhbir KaurInmaculada CousoSukhbir S. MokhaInmaculada SeguraSulaiman S. IbrahimInna LavrikSule CanberkInna P. GladyshevaSumaira HasnainInna S. MidzyanovskayaSuman ChowdhuryInna SolomonovSuman DuttaInna SzékácsSuman GhosalInnocence HarveySuman MukhopadhyayInnokentii E. VishnyakovSuman RanjitIn-Sul HwangSumanta SahooInuk JungSumeet SolankiInyeong ChoiSuming LiIoan SarbuSumit MukherjeeIoana Dorina VlaicuSumit MurabIoana FerecatuSumit Randhir SinghIoana M. IlieSumit SarkarIoana MozosSumit SiddharthIoana StanculescuSun Hee DoIoanina ParlatescuSun Hwa KimIoanna NtaikouSun ImIoannis BakoyiannisSun XiaoIoannis GeorgiouSun Young ParkIoannis GrivasSunao ShojiIoannis IliasSuneel KumarIoannis KostakisSuneet KaurIoannis KostopoulosSung Eun KimIoannis KoutelidakisSung Hee LimIoannis KyrgiosSung Ki LeeIoannis KyrouSung Un HuhIoannis LiampasSung-Bau LeeIoannis MylonasSungeun KimIoannis Ntanasis-StathopoulosSunghan KimIoannis ParaskevaidisSungHee ChoiIoannis PartheniadisSung-Ho GohIoannis PirmettisSungho WooIoannis S. PaterasSung-Hoon KimIoannis SmyrniasSungjin ChungIoannis TheologidisSungjun KimIoannis TsamesidisSung-Lim LeeIoannis VagelasSungsin JoIoannis VamvakarisSung-Soo YoonIoannis XyniasSungsoon FangIoannis ZabetakisSungyoung LeeIoannis-Dimosthenis AdamakisSungyup ChoIolanda De MarcoSun-Hyung LimIolanda FrancoliniSunil GoodwaniIolanda Midea CuccoviaSunil MaloniaIonel MangalagiuSunil S. RaikarIonel SandoviciSunita S. SharmaIonela Andreea NeacsuSunitha KodidelaIonut LuchianSunjae LeeIori SakakibaraSunok MoonIosif MarincuSun-Shin YiIppokratis MessaritakisSun-wook ChoIraia Muñoa HoyosSun-Woong KangIraide AllozaSuowen XuIrena Brčić KaračonjiSupawadee SuksereeIrena Duś-IlnickaSupratim BasuIrena GlazarSupreet AgarwalIrena JanuskaitieneSupriyo RayIrena KratochvilovaSuraj SharmaIrena ManovSurajit BhattacharjyaIrena SmagaSurajit ChatterjeeIrene Andres-BlascoSuran NethisingheIrene BonadiesSurendra RajpurohitIrene CostantiniSuresh KalathilIrene CuadradoSuresh Kumar KailasaIrene GulloSuresh V. AmbudkarIrene Heredero-BermejoSuresh ValiyaveettilIrene PaternitiSurinder M. SoondIrene RamosSurjit SinghIrene RomeroSurya Veerendra Prabhakar VattikutiIrène TatischeffSusan FletcherIrene ValasiSusan L. CampbellIreneusz SowaSusan Philippa WalkerIrfan A. RatherSusana B. BravoIrfan KhanSusana CardosoIrina AnisimovaSusana FerreiraIrina BakloushinskayaSusana García-SilvaIrina BogolyubovaSusana Gómez-BarreroIrina C. AlbulescuSusana M. CardosoIrina D. KonstantinovaSusana P. PereiraIrina Draga CǎruntuSusana RochaIrina FierascuSusana Santos BragaIrina Goldenkova-PavlovaSusanna CogoIrina HeidSusanna SalvadoriIrina KiselevaSusanna ValanneIrina M. KuznetsovaSusanne Burdak-RothkammIrina MakarevitchSusanne FüsselIrina MateiSusanne GrässelIrina MirzaevaSusanne KimeswengerIrina NesmelovaSusanne LutzIrina Neta GostinSusanne MommertIrina PortierSushama SivakumarIrina RistescuSusheel Kumar NethiIrina Roxana NatSushil MishraIrina RusakovaSushmita B. NandyIrina St. LouisSusmita SilIrina V. GuzhovaSusumu MinamisawaIrina VasevaSusumu MitsuyamaIrina ZaidmanSusumu OhyaIrineu BatistaSusy KohoutIrini DoytchinovaSusy LopesIris ForteSuvasini LakshmananIris Z. UrasSuwimol WongsakulphasatchIrit DavidsonSuzana BerendIrma ColomboSuzana K. StrausIrma DianzaniSuzana ŠegotaIrma JärveläSuzanne M. E. LewisIrmgard Irminger-FingerSuzanne PonikIrshad SharafutdinovSuzie LefebvreIryna BenilovaSvatava KubickovaIryna RusanovaSvein-Ole MikalsenIryna V. KosakivskaSven BudikIsaac G. OnyangoSven FlemmingIsaac J. JensenSven ReichenbergerIsaac J. KimseySven SchnichelsIsaac JardinSven T. StrippIsaac Perea-GilSven-Erik BehrensIsabel DuarteSvenja SydorIsabel Espadas VillanuevaSverre Helge TorpIsabel F. AmaralSvetlana A. DemyanenkoIsabel Faria-RamosSvetlana AntonyukIsabel Kolm-DjameiSvetlana FaIsabel LarreSvetlana JovanovićIsabel LegazSvetlana KarakhanovaIsabel MarquesSvetlana KryštofováIsabel RodríguezSvetlana MarkovaIsabel SilveiraSvetlana N. SushkovaIsabel VeladaSvetlana P. ChapovalIsabela T. PereiraSvetlana S. EfimovaIsabella BarbieroSvetlana SaikovaIsabella DerlerSvetlana TamkovichIsabella PallottaSvetlana UzbekovaIsabella PanfoliSvetlozar VelizarovIsabella PigaSvetoslav DimovIsabella RomeoSveva BolliniIsabella SaggioSwagat SharmaIsabella SalzerSwapan ChakrabartyIsabella SperdutiSwapna Priya RajarapuIsabella ZanellaSwapnalee SarmahIsabella ZironiSwapnil BawageIsabelle AudoSwapnil Ganesh SanmukhIsabelle Brun-HeathSwarup RoyIsabelle CheminSwarup Roy ChoudhuryIsabelle Galy-FaurouxSwastik PhuleraIsabelle HueSwati JainIsabelle J. SchalkSwayam Prakash SrivastavaIsabelle LeclercqSwee Leong SingIsabelle LemassonSwee-Suak KoIsabelle PerraultSweilem B. Al RihaniIsabelle PlanteSyam SomasekharanIsaiah ArkinSybille HasseIsaiah Catalino M. PabuayonSydney AtenIsao IshiiSydney TraskIsao MatsuokaSyed Adeel ZafarIsha MutrejaSyed Farooq AdilIshita ChatterjeeSyed Haris OmarIshwarlal JialalSyed Husain Mustafa RizviIsidro FerrerSyed ImamIsidro Vitoria MiñanaSyed J. KhundmiriIslam El-SharkawySyed MehdiIsmael RodrigoSyed Mianİsmail ÖçsoySyed Saad B. QasimIsra MareiSygkliti-Henrietta PelidouIsrael AlshanskiSylvain BourgoinIsrael D. J. SampaioSylvain De BreyneIsrael GonzálezSylvain LatourIsrael SeklerSylvain LegrandIsrafil KucukSylvain MarcelIssac SachmechiSylvain ProvotIstván BaczkóSylvère StörmannIstván BaloghSylvette Chasserot-GolazIstvan KrizbaiSylvia C. HewittIstvan NagySylvia FaictIstván OsztheimerSylvie AmuIstván PetákSylvie BouvierIstván SzilágyiSylvie CoscoyI-Ta LeeSylvie DucreuxItahisa Marcelino-RodríguezSylvie FriantItalia FalconeSylvie MazanItamar SimonSylwester GawinkowskiItaru KatoSylwia BalutaI-Tsang ChiangSylwia BlochItzik CooperSylwia BudzynskaIulia PinzaruSylwia Katarzyna KrólIulia-Cristina BagiuSylwia Męczyńska-WielgoszIva LakićSylwia MichlewskaIva PichováSylwia OkońIva SovadinováSylwia Olimpia RzońcaIvaldo ItabaianaSylwia ProchowskaIvan A. PaponovSylwia StudzinskaIvan Ayuso-FernandezSylwia StudzińskaIvan BarvikSylwia WrotekIvan ChenchevSylwia ZielińskaIván ConejerosSzabat MartaIvan ConteSze Wan Samantha ShanIvan Cruz-ChamorroSzereday LászlóIvan De Godoy MaiaSzu-Cheng KuoIvan HirschSzu-Tah ChenIvan KodrinSzymon JanczarIvan KosikSzymon KubalaIvan KreftSzymon SękowskiIvan Krešimir SvetecSzymon SkoczeńIvan KushkevychT. A. KuferIván M. MoyaT. Bucky JonesIvan ManziniT. L. DuarteIvan NombelaT. P. KukinaIvan RadosavljevicTabito KinoIvan S. KiselevTadahisa TeramotoIvan SalazarTadaho NakamuraIvan Shterev DonevTadaomi FurutaIvan SpasojevicTadashi SatohIvan TarakanovTadashi WatabeIvan Yu. ChernyshovTadashi YoshidaIvan Yu. SkvortsovTadayoshi BesshoIvana AntonucciTadayuki OshimaIvana BiljanTadeja ReženIvana BjelobabaTadeusz InglotIvana GobinTadeusz IssatIvana HálováTadeusz KamińskiIvana JarakTadeusz MalewskiIvana JochmanováTadeusz PawełczykIvana JukićTadeusz PolonskiIvana KavecanTae Hyun KangIvana KholováTae Nyoung ChungIvana KolackovaTae Sung JungIvana KurelacTaebo SimIvana NovakovicTae-Bong KangIvana PeranTaeck JeonIvana PerkovicTae-Don KimIvana SamarzijaTae-Gyu LimIvana SemovaTae-Hong KangIvana TomazTaejong PaikIvana TrapaniTae-Ju ChoIvana Vinkovic VrčekTaemin KimIvana ViolaTaeok BaeIvayla DinchevaTaesic LeeIvelina TsachevaTae-Yub KwonIveta HerichovaTaha AzadIveta Y. YotovaTahseen H. NastiIvette Buendía RoldánTaichi MatsubaraIvica PelivanTai-Chia ChiuIvo HornTaichiro GotoIvo OliveiraTaichiro NonakaIvo PiantanidaTaiho KambeIvone MartinsTai-Li ChenIvonne NelTai-long PanIwata OzakiTaira MiyaharaIwona Bednarz-MisaTaisen IguchiIwona Ben-SkowronekTaiyo ToribaIwona Cichowska-KopczyńskaTajana FilipecIwona CiechomskaTakaaki SenbonmatsuIwona CiereszkoTakaaki ShimohataIwona DąbkowskaTakaaki TomofujiIwona Jarocka-KarpowiczTakafumi HaraIwona KarbownikTakahiro InoueIwona KarwaciakTakahiro KannoIwona ŁakomskaTakahiro KodamaIwona RadziejewskaTakahiro MatsukiIwona SaduraTakahiro NemotoIwona ŚcibiszTakahiro SatoIyyakkannu SivanesanTakahiro UchidaIzabela MakalowskaTakahiro YamasakiIzabela Papiewska-PajakTakahisa HiramitsuIzabela PawłowiczTakahito MukaiIzabela RaganTakahito OhshiroIzabela ZakrockaTakakazu MitaniIzabella OlejniczakTakako OsakiIzabella Slezak-ProchazkaTakako YasudaIzhar Hyder QaziTakaku MotokiIzortze SantinTakamasa SuzukiIztok TurelTakamichi ItoIzumi HorikawaTakamitsu A. KatoIzumi KajiTakamitsu KatoIzumu SaitoTakanari NakanoJ. Adolfo García-SáinzTakane Kikuchi-UedaJ. Arjuna RatnayakaTakanori IdaJ. Cameron MillarTakanori KuboJ. Catharina DuvigneauTakao HibiJ. David WarrenTakao KanamoriJ. Kelly SmithTakao MorinagaJ. Mark WeberTakao ShimazoeJ. Matthew HinkleyTakao TsunedaJ. Michael SorrellTakashi AokiJ. U. CarmonaTakashi HimotoJaakko L. O. PohjoismäkiTakashi HoshibaJaap G. NeelsTakashi ItoJabadurai JayapaulTakashi KanbayashiJacek BiałowąsTakashi MatozakiJacek DrobnikTakashi MisawaJacek E. NyczTakashi MuramatsuJacek FrancikowskiTakashi NishidaJacek GębickiTakashi OnoJacek KozdrójTakashi SaitoJacek KwiecienTakashi TodaJacek PiatekTakashi TsukamotoJacek PiosikTakashi UebansoJacek R. WilczyńskiTakatsune ShimizuJacek RóżańskiTakaya MoriguchiJacek SkarżewskiTakayo OtaJacek StępniewskiTakayoshi WakagiJacek TabarkiewiczTakayuki MurataJacek ZebrowskiTakayuki NegishiJack Christopher LeoTakayuki TsukubaJack FeehanTakeaki OzawaJack H. RyanTakehiko KanazawaJack HarrowfieldTakehiko MatsushitaJack J. W. A. van LoonTakehiro KawashiriJack M. WebsterTakeji UmemuraJacky LooTakemi OtsukiJacob C. LutterTakemichi FukasawaJacob KageyTakeo MukaiJacob LandisTakeshi A. OnumaJacob Lorenzo-MoralesTakeshi AibaJacob RaberTakeshi HanoJacob Stewart-OrnsteinTakeshi HashimotoJacob WilsonTakeshi KamiyaJacobo SellarésTakeshi KikuchiJacomine K. Krijnse-ĹockerTakeshi NishimuraJacopo IacovacciTakeshi NodaJacopo Junio Valerio BrancaTakeshi SasakiJacopo LamannaTakeshi SatoJacopo MeldolesiTakeshi TokuyamaJacopo SabbatinelliTakeshi UemuraJacopo SgrignaniTaketo YamadaJacqueline CapeauTaku KouroJacqueline FindlayTaku OzakiJacqueline M. BentelTaku ShojiJacqueline ReinhardTaku YoshiyaJacqueline W. MaysTakuhiro OtosuJacques A. NunèsTakuji KurimotoJacques GilloteauxTakuji ShodaJacques PouyssegurTakuji TanakaJacques PouysségurTakujiro HommaJacques RobertTakuma MatsubaraJade Jue ShiTakumi IshizukaJadranka MilikićTakumi TakataJadwiga LaskaTakuo EmotoJadwiga WyszkowskaTakuro HoriiJae Ho LeeTakuro NakagawaJae Hoon MoonTakuro NakamuraJae Hwan JungTakuya FurutaJae Min ChoTakuya IyodaJae Sung ShimTakuya OmoteharaJaechan LeemTakuya TeraiJaehan KimTalma BrennerJae-Ho LeeTam PaulJae-Ho ShinTamako HataJaehoon SimTamar KeasarJae-Hyung LeeTamara BotoJae-Hyung ParkTamara PečenkováJae-kwan SongTamara TuuminenJaemin LeeTamás BíróJae-Min OhTamás GállJaeseok HanTamas KálaiJae-Seong YangTamás KőszegiJaeson CallaTamas KovacsJaesung KimTamás Kovács-ÖllerJa-Eun KimTamas KoziczJaewhan SongTamás LakatosJaewon JangTamás LetohaJae-Yean KimTamás LőrinczJae-Yeol JooTamás TornóczkyJaeyoon KimTamer MohamedJafar JafariTamerlan T. MagkoevJafar RezaieTamil ThangavelJafar SharifTaminori ObayashiJagadeesh K. VenkatesanTamis DarbreJagoda LitowczenkoTammy M. MartinJai S. RohilaTamotsu HoshinoJai Young ChoTamsyn MontagnonJai-Hong ChengTáňa RavingerováJaime Rubio-MartinezTanaka TeruyukiJain PriteshTancu Ana Maria CristinaJaiprakash SharmaTaner SarJaiprasath SachithanandhamTang Her JaingJaison JeevanandamTania C. SorrellJakir HossainTania ColasantiJakob NäslundTania FiaschiJakob ReiserTania GamberiJakub FamulskiTania MaffucciJakub HadzikTania Martins-MarquesJakub NalepaTania PenchevaJakub RokTania Quesada-LópezJakub SuchodolskiTania RomachoJakub SzperlikTânia S. MoraisJakub ZdartaTania SilvaJalal IsaadTanima BoseJamal BouitbirTanima DeJames A. BunceTanja GrotenJames A. CarrollTanja Grubić KezeleJames A. MarrsTanja KunejJames AdjayeTanja Nicole HartmannJames AngelastroTanja RestinJames B. GibsonTanmay GhongeJames B. HurleyTanmoy MajiJames BonnerTanner O. MonroeJames BrasicTanos C. C. FrançaJames C. L. ChowTanumoy MondolJames CroninTanushree DangiJames D. SutherlandTanveer Ahmad TabishJames DavisTanya A. WagnerJames DoutchTanyalak ParimonJames E. McLarenTao LinJames FrelichowskiTao LiuJames G. RavinTao SuJames HoTao TongJames Ian RobinsonTao WangJames Isaac CohenTao YangJames K. HartsfieldTao ZhangJames Kumi-DiakaTao-Hsin TungJames L. CookTâp Ha-DuongJames LiTara G. McDaneldJames M. HymanTara M. StruttJames M. Kunert-GrafTara McmorrowJames MetcalfTaras P. PasternakJames P. BennettTarek Mohamed Abd El-AzizJames P. DilgerTariq H. IqbalJames ScholeyTarique BagalkotJames SherwoodTaro ShimizuJames SleighTaronish Dorab DubashJames StewartTarsila G. CastroJames V. AquavellaTarun JaiRaj NarwaniJames V. GruberTasha Marie Santiago-RodriguezJames VesenkaTaslima HaqueJames W. BarlowTasneem P. SharmaJames WestTata Nageswara RaoJames WilliamsTateaki NaitoJamie O′RourkeTatiana A. KorolenkoJan BakosTatiana ArmeniJan BilskiTatiana AstrelinaJan BocianowskiTatiana EfimovaJan BrabekTatiana KostrominovaJan Czesław KotarskiTatiana M. VasilievaJan DanserTatiana MatveevaJan DörrieTatiana MinkinaJan E. AzarovTatiana S. FrolovaJan E. GrantTatiana ScorzaJan E. KammengaTatiana ShtamJan FílaTatiana TozarJan GebauerTatiana V. IlchibaevaJan GurskyTatiana V. ShelengaJan Jacek KaczorTatiana ZamayJan KorbeckiTatijana ZemunikJan KrajczewskiTatjana ArsenijevicJan KrijtTatjana G. ShibaevaJan Krzysztof NowakTatjana RadosavljevićJan KučeraTatsuhiko FurukawaJan KuhlmannTatsuhiro SatoJan KvasnickaTatsuhito MatsuoJan LakotaTatsuki KataokaJan LaRocqueTatsuki KunohJan LehotskyTatsuki R. KataokaJan LindemanTatsuo KandaJan Lj MiljkovićTatsuo KawaiJan ŁyczakowskiTatsuo MichiueJan MacutkevičTatsuo SatoJan MieszkowskiTatsuo ShimosawaJan Philipp BewersdorfTatsuro MutohJan Philipp MenzelTatsuro OhiraJan PithaTatsuya AbeJan PodkowińskiTatsuya HigakiJan R. T. Van WeeringTatsuya MorimotoJán RemšíkTatsuya ShirahataJan ŠevčíkTatsuya UsuiJan SkodaTatyana KolesnikovaJan StępniakTatyana L. AzhikinaJan TesarikTatyana MerkulovaJan WeberTatyana MeyersJan WijnholdsTatyana O. PleshakovaJan ZunaTatyana PasechnikJana FrankováTatyana V. SavchenkoJana JakubíkováTautgirdas RuzgasJana JanockovaTaxiarchis KatsinelosJana LibantováTeayoun KimJana MoravcikovaTécher HervéJana NekolováTeeratas KijpornyongpanJana PěknicováTeet SeeneJana PourováTeikichi IkuraJana RieggerTeja Muralidhar KalidindiJana SedlaříkováTe-Ling LuJana StanicováTelma QuintelaJana TchekalarovaTengteng YuJanak JoshiTeodor RusuJanaki IyerTeodora Alexa-StratulatJane A. LeopoldTeodora Emilia ColdeaJane DeverTeodorico C. RamalhoJane MurrayTeodorico De Castro RamalhoJane WelshTeodoro Coba De La PeñaJanet AlderTeofil JesionowskiJanet LighthouseTerenzio CosioJanet RubinTeresa AltabellaJanez MravljakTeresa AuguetJanez PlavecTeresa CardosoJanice ForsterTeresa CoutinhoJanice M. PluthTeresa DocimoJanina Gajc-WolskaTeresa Donze-ReinerJanina GospodarekTeresa FascianaJanine DanksTeresa GriecoJanine KahTeresa GrzelakJanine MeienbergTeresa P. SilvaJanine Van GilsTeresa PinheiroJanis MüllerTeresa RodriguesJanja TrčekTeresa SalvatoreJanja ZupanTeresa Sosa DíazJanna G. KoppeTeresa SummavielleJanne A. IhalainenTeresa TrottaJanne KlitgaardTeresa ŻołądekJanne KoivistoTeresa ŻołekJános AlmássyTereza GoliasJanos KanczlerTerezia KiskovaJános KappelmayerTerrence PivaJanos M. KanczlerTerrone RosenberryJanosch EhrenmannTerry D. HindsJanosch HellerTerry GordonJanusz BlasiakTerunao TakaharaJanusz Franco-BarrazaTetiana A. ZaǐchukJanusz JacakTetsuro IshiiJanusz NiedojadłoTetsuya HirataJanusz SzklarzewiczTetsuya KakoJaquelin P. DudleyTetsuya SuharaJara Pérez-JiménezTetyana P. BuzhdyganJared C. RoachTe-Yao HsuJared MayThais B. RodriguesJari LouhelainenThalita M. C. NishimeJarmila CelakovskaThanh Hai LeJarmila JedelskáTharmarajan RamprasathJarogniew J. ŁuszczkiThea MagroneJarogniew ŁuszczkiTheerut LuangmonkongJaromír MyslivečekThekla CordesJaromír VašíčekTheo M. De ReijkeJaroslav ČermákTheo ReinJaroslav PavlůTheocharis G. KonstantinidisJaroslav TruksaTheodora CalogeropoulouJaroslav V. BurdaTheodora FarmakiJaroslaw BaranTheodora PapamitsouJarosław BłaszczykTheodore W. ThannhauserJaroslaw CalkaTheodoros EleftheriadisJarosław CzyżTheodoros RampiasJarosław J. SakTheodosia MainaJarosław L. PrzybyłTheoni KanellopoulouJaroslaw PaluszczakTheoni MargaritopoulouJaroslaw PanekTheophilos IoannidesJarosław PasekTheresa HautzJarosław PoznańskiTheresa PohlkampJaroslawa RutkowskaTherese WilhelmJar-Yi HoTheresia ThalhammerJasenka Antunović DunićThi Tuong Vy PhanJasinski-Bergner SimonThiago J. BorgesJasmina Lukinac ČačićThiago Wendt ViolaJasmine SinhaThibault KervarrecJasminka ŠtefuljThibault ViennetJasna MetovicThierno Madjou BahJason C. SteelThierry CensJason C. YoungThierry HauetJason E. BruemmerThierry MagnaldoJason E. SwainThilina U. JayawardenaJason EarThiru VanniasinkamJason GardinerThiruganesh RamasamyJason H. YangThom HuppertzJason KarchThomas A. LagaceJason L. BlumThomas AchJason LanoueThomas AndlJason LiThomas B. B. JacobsJason M. BelitskyThomas BadetJason MagnusonThomas BalligandJason P. SchwansThomas BrandJason ParsonsThomas D. BurleighJason R. HerrickThomas DandekarJason StewartThomas DechatJason Thomas DuskeyThomas DippongJasper YikThomas DittmarJathish PonnuThomas DresbachJaume TorresThomas F. MooreJavad AlizargarThomas FalguièresJavad TavakoliThomas FlaggJaved AhmadThomas FranchiJavier A. Linares-PasténThomas GreitherJavier Álvarez MartínThomas GrewalJavier AnguloThomas GudermannJavier BrumosThomas H. MasseyJavier C. AnguloThomas HellwegJavier Caballero-VillarrasoThomas HieronymusJavier Conde ArandaThomas HollemannJavier Eduardo Garcia CastañedaThomas J. MarianiJavier Fernández-RodríguezThomas J. SmithJavier Fernández-RuizThomas J. TuckerJavier Gallego-BartoloméThomas KelleyJavier GarzónThomas L. MindtJavier Leon SerranoThomas LiehrJavier Martinez-UserosThomas LuxJavier Menéndez-MenéndezThomas M. HarrisJavier Molina-CerrilloThomas MaginJavier MoragaThomas MittmannJavier OrozThomas NavesJavier Quílez-BermejoThomas NeumannJavier Redondo-MuñozThomas NussbaumerJavier Rodríguez VillanuevaThomas PabstJavier Segarra-MartiThomas PatersonJavier UserosThomas ProftJaw-Yuan WangThomas R. CaulfieldJay H. ChungThomas RaabeJay S. MishraThomas ReiterJay V. PatankarThomas RoscoeJaya AseervathamThomas S. WeissJaya PadmanabhanThomas SchlathölterJaya Prakash GollaThomas SimonJayachandran VenkatesanThomas TheisJayanta Kumar PatraThomas ThiebaultJayanthi ParthasarathyThomas W. PriceJayapal Reddy MallareddyThomas WestermaierJayarama GunajeThomas WilkieJayaraman BalamuruganThomas. O. KragJayasimha Rayalu DaddamThor EysteinssonJaycob D. WarfelThorsten GnadJaydeep BhatThorsten KirschJayson R. BamanThorsten KraskaJean A. RondalThorsten MaretzkyJean AmiralThorsten R. DöppnerJean BoutinThu DuongJean CadetTiago A. FernandesJean Christopher ChamcheuTiago DiasJean De La RosetteTiane C. FinimundyJean DemarquoyTianhu LiJean Luc OlivierTianhua NiuJean Michel DavièreTiara Bunga Mayang PermataJean Michel MaixentTibor BruntJean Michel PouvesleTibor HianikJean MukherjeeTibor HortobagyiJean VanderpasTibor JandaJean-Alain FehrentzTibor KrenácsJean-Baptiste BouléTibor PankotaiJean-Daniel MalcorTibor RohacsJeanette GrundströmTien Duc PhamJeanette LeusenTiffany F. KautzJean-François HernandezTiffany KhongJean-François HocquetteTiffany WillsJean-François PicimbonTihana Lenac RovisJeanine R. Jarnes-UtzTihana MarčekJean-Jacques MonsuezTihomir SolomunJean-Louis DacheuxTiina KelkkaJean-luc DavignonTilili BarhoumiJean-Luc MorelTill DammaschkeJean-Luc PoyetTilman SchlothauerJean-Marc BarretTilman ZieglerJean-Marc ChatelTim FosterJean-Marc LancelinTim PagetJean-Marie ExbrayatTim StevensJean-Marie RuysschaertTim WilliamsJeanmarie VerchotTimo GaberJean-Michel GuenetTimo Homeier-BachmannJean-Michel SiaugueTimo W. M. De GroofJeannine DieschTimofei ZatsepinJeannine R. LaRocqueTimofey V. MalyarenkoJean-Paul BourdineaudTimothy ArtlipJean-Paul MottaTimothy CorsonJean-Paul NobenTimothy D. VadenJean-Philippe BerteauTimothy EricksonJean-Philippe HaymannTimothy EubankJean-Pierre GagnerTimothy HopkinsJean-Pierre JulienTimothy HughesJean-Pierre RaufmanTimothy J. PetrosJean-pierre Saint-JeannetTimothy J. ShaferJean-Pol FrippiatTimothy JaromeJean-Sébastien RougierTimothy John MahonyJean-Yves RoignantTimothy John TranbargerJed LampeTimothy L. MegrawJedrzej GrzegrzołkaTimothy P. NewsomeJee Hyun KimTimothy R. HooverJee Myung YangTimothy SandsJee Taek KimTimothy SnowdenJeella AcedoTimothy W. KraftJeevisha BajajTimur FatkhudinovJeeyoun JungTina BeyerJeff C. W. WangTina Di PalmaJeff HollyTina GumiennyJeff WiluszTina McKayJeffery HainesTine Van BergenJeffery S. TessemTing HanJeffrey BraultTing LiJeffrey BrenderTing WeiJeffrey De VeroTingting HongJeffrey F. ScottTing-Wen ChenJeffrey HordTingyu ChangJeffrey I. PennyTing-Yu LiuJeffrey MillerTing-Yun LinJeffrey R. AndolinaTinhinane FaliJeffrey R. LiddellTin-Tin Win-ShweJeffrey V. LeytonTirupapuliyur DamodaranJeffrey W. BrownTissa WijeratneJeffrey WangTiziana AlberioJeffrey WigleTiziana CiarambinoJegan IyyathuraiTiziana De CristofaroJehad KharrazTiziana FuocoJehan AlamTiziana SchioppaJehan El-JawhariTiziano MarzoJekaterina ErenpreisaTiziano VerriJelena MandićTobias DallengaJelena MijalkovićTobias GorisJelena Osmanović BarilarTobias LangeJelena Popović ĐorđevićTobias LortzingJelena ViderTobias MaetzigJelle Van Den AmeeleTobias MeitzelJemima MellerioTobias ReiffJen-Chih TsengTobias StauberJender WuTobias StraubJeng-jer ShiehTobias StrunzJennifer AltomonteTod StuessyJennifer CaseyTofayel AhmedJennifer D. Foulke-AbelToh Tan BoonJennifer FangTohru SuzukiJennifer Geddes-McAlisterToivo MaimetsJennifer GreenwichTojung TsengJennifer GubitosaTokuhiro ChanoJennifer L. FreemanTokuko HaraguchiJennifer MytychTom C. MartinsenJennifer O. ManilayTom DariusJennifer PattersonTom HansenJenning TsaiTom HuxfordJenn-Wei ChenTom IrvingJenny BulgarelliTom J. H. ArendsJenny EkbergTom KaragiannisJens André HammerlTom L. BroderickJens BosseTom P. FlemingJens Christian MöllerTomas BlancoJens GeorgTomás González-LezanaJens HahneTomáš HavránekJens KlockgetherTomáš HluskaJens SchäferTomás José González-LópezJens SchlossmannTomáš KašparovskýJens SmiatekTomas NecasJens-Uwe GrabowTomas OlejarJen-Tsung ChenTomas PospisilJeong A. ParkTomás Rocha-RinzaJeong Eon LeeTomas SimurdaJeong June ChoiTomas TorrobaJeong Ki MinTomas VinarJeong-An GimTomáš VyhnánekJeong-Chae LeeTomaso BottioJeong-hae ChoiTomasz BajdaJeong-Hoon LimTomasz BlicharskiJeongil YuTomasz BondaJeongkwon KimTomasz CzechowskiJeongmin LeeTomasz DziodzioJeongsik YongTomasz FrączykJeong-Tae KohTomasz FrancuzJeppe EmmersenTomasz GizewskiJeppe PrætoriusTomasz GrzywaJera JerucTomasz GubicaJer-An LinTomasz J. PrószyńskiJeremias MotteTomasz KluzJeremie D. OliverTomasz KockiJérémie GautheronTomasz KoczorowskiJeremy AstierTomasz KoziorJeremy BellienTomasz M. KarpinskiJérémy CheretTomasz MisztalJeremy ChienTomasz MroczekJeremy CouturierTomasz NiedzielaJeremy D. MarksTomasz PanczykJéremy DenizotTomasz PoplawskiJeremy GoldmanTomasz RydzkowskiJeremy LagrangeTomasz SadkowskiJeremy P. K. TanTomasz SarnowskiJeremy TurnbullTomasz SawickiJeremy WidemanTomasz SkrzypekJernej StareTomasz SledzinskiJeroen T. BuijsTomasz SzymborskiJérôme BusserollesTomasz TokarekJérôme E. RogerTomasz W. KaminskiJérôme EeckhouteTomasz WarzechaJerome H. L. HuiTomasz WentaJerome LafontTomasz WypychJerome MontnachTomasz ZąbekJerome PaggettiTomasz ŻokJerome StraussTomaž JagričJerónimo GonzálezTomica HrenarJerran SantosTomislav MestrovicJerry VockleyTomislav RončevićJerry W. SimeckaTomita KazunoriJersson PlacidoTommaso De MarchiJerzy BeltowskiTommaso GoriJerzy ChudekTommaso LombardiJerzy JuśkiewiczTommaso M. ManziaJerzy WegielTommaso NeriJesper BomanTommaso TabaglioJesper JørgensenTommaso ZandriniJesse DeereTommy BaumannJesse GoyetteTomoaki ShintaniJessica Aijia LiuTomoaki TakataJessica BarsonTomofumi YamamotoJessica BauerTomohiko AoeJessica BurketTomohiro BitoJessica D. HohensteinTomohiro MizobataJessica Dal ColTomohiro OsakiJessica GluckTomohiro SawaJessica GuerraTomohisa IshikawaJessica L. CaldwellTomokazu KonishiJessica LowethTomoki HoshinoJessica M. LohmarTomoko HasegawaJessica MaiuoloTomoko TakanoJessica PepeTomomi FujiiJessica RosatiTomomi TakanoJessica YoungTomomi YamazakiJessy A. SlotaTomomitsu MiyagakiJesús Cosín-RogerTomonori IyodaJesus Delgado-CalleTomonori WakuJesús DevesaTomoya EsumiJesus Fabricio Guayaquil-SosaTomoyo OkadaJesus Jiménez-BarberoTomoyuki KawamuraJesús Jiménez-RuizTong FeiJesus M. Martin-CamposTong-Hong WangJesús M. MíguezToni GiorginoJesús María VielbaToni GossmanJesús OsadaTonino TrainiJesús PalomeroTony HeurtauxJesus PastorTor Henrik TvedtJesus RequenaTore SkotlandJesus V. Jorrin-NovoTorill SauerJesus Vicente De Julián OrtizTorry A. TuckerJet BliekToru IshikawaJetty Chung-Yung LeeToru IwasaJe-Wen LiouToru TakahashiJe-Yoel ChoToru TatenoJi Heui KimToru TogawaJi Hoon JungToru YagoJi Hyeon AhnToshana Lauria FosterJi Hyeon JuToshiaki AraJi Hyun ParkToshiaki ArataJi Yeon KimToshiaki OharaJi Youn YooToshifumi TsukaharaJia L. SongToshiharu YamamotoJia Ping WuToshihide KuriharaJia RongToshihiko TashimaJia Rong JhengToshihiro KushibikiJia XiongToshihiro SakuraiJiabing FanToshihiro TsurudaJiacheng SunToshihisa MizunoJia-Feng ChangToshikazu SuzukiJiale YangToshinori YoshiharaJialei DuanToshio HattoriJian Q. YuToshio TakahashiJian ShiToshiro AraiJian TengToshitsugu FujitaJian ZhouToshiya TakahashiJianbin SuToshiyuki FukadaJiandong ChenToshiyuki KajiJianfeng LiToshiyuki KawaiJiang PiToshiyuki MatsuzakiJiangang ChenToshiyuki MitsuiJiangchuan ShenToshiyuki SetoJiangning YangToshiyuki YonedaJiang-Yun LuoTosso LeebJianhua XiongTou Cheu XiongJianhui ZhuTousif SultanJianjun WenToyofumi SuzukiJiankai LuoToyota KenjiJianmin SunTozzini Eva TerzibasiJianming KangTracey MadgettJiann-Chu ChenTraci MarinJiann-Jou YangTracy A. BrooksJianqiang ShenTracy Murray StewartJian-Yong WuTran Dang XuanJianzhou CuiTrevor CreamerJiao Jiao LiTrevor D. RapsonJiao WangTrevor RomsdahlJiarul MidyaTriantafyllos DidangelosJiashen LiTrim LajqiJiaxu LiTrine L. Toft-BertelsenJie GaoTrinidad De MiguelJie LeTrinidad Ruiz TéllezJie YangTripti SharmaJie ZhengTrish BergerJigar ModiTrond AasenJihane BasbousTroussard XavierJihane Homman-LudiyeTroy D. WoodJih-Kai YehTsan-Chi ChenJihong ParkTse-Min LeeJi-hwan LeeTsing BohuJiiang-Huei JengTsuchida SachioJikui GuanTsukasa OkiyonedaJill de JongTsunahiko HiranoJill JohnsonTsuneaki SadanagaJill MadineTsuneo IshidaJill P. G. UrbanTsung-Chuan HoJillian M. CarrTsung-Han ChouJimmy AlarcanTsung-Hsien LeeJin Ah ChoTsung-Hsuan LaiJin Joo ChaTsung-I HsuJin Kwon JeongTsung-Jen WangJin WangTsung-Jung LiangJin WeiTsung-Rong KuoJin ZhangTsun-Thai ChaiJinbao ZhangTsutomu HatanoJinbiao ChenTsutomu KatayamaJinbin XuTsutomu OmatsuJin-Bon HongTsu-Wei ChenJindřich ChmelařTsuyoshi HattoriJing TangTsuyoshi KimuraJing ZhangTsuyoshi ShutoJingfeng HuangTsuyoshi SugiuraJinghua WangTsvetan R. BachvaroffJingjing TangTsvetelina VelikovaJing-Jy ChengTsvetoslav GeorgievJingKai WeiTsz Kin Martin TsuiJingquan WangTufail BashirJingrong WangTugba KucukkalJingshan S. DuTuire SalonurmiJingtao ZhangTuli MukhopadhyayJingwei ChengTullia MaraldiJing-Wen ShihTullio FlorioJingyu ZhangTullio GenovaJin-Ha ChoiTünde FeketeJinhee LimTuo HuJin-Ho KohTuomas LönnbergJinhong MengTuomas NäreojaJin-Ichi InokuchiTurcu-Stiolica AdinaJinju ChenTushar TomarJinku KimTusty-Juan HsiehJinli WangTuula NymanJinling HuangTuyen T. DangJinpeng LiTvrtko HudolinJinsong HaoTwan RuttenJinu HanTylor R. LewisJinwei ZhangTzer-Bin LinJin-woo KimTzeta HuangJin-Wu NamTzibun NgJinxiang XiTzong-Yuan WuJin-Yi WuTzong-Yueh ChenJin-Yong LeeTzu-En LinJiong LiTzu-Shing DengJiraporn LueangsakulthaiUbaldo ArmatoJiri BarekUday KishoreJiří ČerveňUday Praful KundapJiří FajkusUdo BakowskyJiri FrimlUdo BläsiJiri HejnarUdo JeschkeJiri HrdyUdo KontnyJiří JiráčekUdo S. GaiplJiri MlcekUdo SchumacherJiri VondrasekUeli SchiblerJiro TakitoUfuk GunesdoganJiseok LimUgo CarraroJitendra KanaujiyaUgo Lionel MoensJitendra KumarUgo PerriconeJitendra ThakurUgo TestaJitendra TripathiUjendra KumarJitka ForstovaUjjal Kumar BhawalJiuann-Huey Ivy LinUlf AndereggJiun-Lin WangUlf GeumannJiunn- Liang KoUlf GuentherJiwon M. LeeUlf HanefeldJi-Won ParkUlf PetterssonJi-won RyuUlises Gomez-PinedoJixiang ZhangUlrich BaumgartnerJixu LiUlrich BlankJiyang CaiUlrich DobrindtJiyeon ChoiUlrich GergsJiyoung LeeUlrich SackJo LeroyUlrich WalterJo LewisUlrika Marie NordströmJo PerryUlrika Von DöbelnJo SourbronUlrike BacherJoab ChapmanUlrike BaschantJoachim GeyerUlrike GrünertJoachim KrebsUlrike ReschJoachim MüllerUlrike Rolle-KampczykJoachim RassowUlrike TopfJoachim StorsbergUlvi MoorJoachim VollbrechtUma K. AryalJoan CalvetUmang SwamiJoan EstelrichUmberto GalderisiJoan OlivaUmberto LaforenzaJoan Roselló-CatafauUmberto MalapelleJoan SeguraUmesh S. DeshmukhJoan Torras-AmbròsUmesh TharehalliJoana CaetanoUmeshkumar AthiramanJoana F. SacramentoUmut CaginJoana GasparUna RiekstiņaJoana M. O. SantosÜner KolukisaogluJoana MicaelUnni GrimholtJoana OliveiraUpendar GollaJoana RelatUpendra KatneniJoana RoloUrban BrenJoana SilvaUrmas ArumäeJoanna BogusławskaUrmi RoyJoanna BoniorUrsula WynekenJoanna BorgognaUrszula CytlakJoanna BorowiecUrszula DoboszewskaJoanna BrzeszczUrszula HohmannJoanna BukowskaUrszula ŁapińskaJoanna CabajUrszula LisieckaJoanna ChiuUrszula SzymanowskaJoanna Chorostowska-WynimkoUrszula WachowskaJoanna CzarneckaUrszula WnorowskaJoanna DąbrowskaUrszula WydroJoanna DepciuchUsategui-Martín RicardoJoanna DłużniewskaUta SchnabelJoanna Domagala-KulawikUte RömlingJoanna FilipowskaUtkarsh H. AcharyaJoanna Gdula-ArgasińskaUttara SaranJoanna Gromadzka-OstrowskaUwe RitterJoanna KaminskaUwe SteinhoffJoanna KarbowniczekUwe WolinaJoanna KaszubaV. E. OleynikovJoanna KolmasV. Praveen ChakravarthiJoanna KonopińskaVáclav VětvičkaJoanna KosackaVadim B. KrylovJoanna KovalskiVadim KhlestkinJoanna KozłowskaVadim Le JoncourJoanna L. SelfeVadim PokrovskyJoanna M. HaraznyVadim SumbayevJoanna Maria Souza-FabjanVadim VolkovJoanna MikloszVagner Ricardo LungeJoanna MoraczewskaVahan KépénékianJoanna MystkowskaVahid FarzanehJoanna ObaczVahideh RabaniJoanna OrtylVaibhav TiwariJoanna RossowskaVaidotas StankevičiusJoanna Skorko-GlonekVaishali AggarwalJoanna SolichVaishali KapoorJoanna StragierowiczValentin DjonovJoanna StrandValentin HerberJoanna TimminsValentin LobyshevJoanna WieczfińskaValentina BonettoJoanna WojtkiewiczValentina BoscaroJoanna WróblewskaValentina BravatàJoanna Zawacka-PankauValentina CalòJoanna ZemłaValentina CaputiJoanne BoldisonValentina CataniaJoanne HarveyValentina CecariniJoanne KeenanValentina Dell′OsteJoanne TobacmanValentina DincaJoão BotelhoValentina DoldiJoão C. SousaValentina DomeniciJoão Carlos Caetano SimõesValentina FanelliJoão Carlos RamosValentina G. MatveevaJoao GoncalvesValentina GargiuloJoão Guilherme CostaValentina GentiliJoao LoboValentina GiudiceJoão M. SantosValentina KovalevaJoao MamedeValentina LanteriJoão P. L. DaherValentina LatinaJoão Pedro BarbasValentina LiakinaJoão Pedro Costa-NunesValentina LulliJoao R. GomesValentina MarchicaJoao Ramalho-SantosValentina MassaJoão Ramalho-SantosValentina OnnisJoao Santos-AntunesValentina PallottiniJoao T. S. BotelhoValentina PavoneJoão TeodoroValentina PileczkiJoão-Paulo-Mendes TribstValentina PirotaJoaquim CarrerasValentina RussoJoaquim EgeaValentina SacconeJoaquim OristrellValentina SalaJoaquim VivesValentina ScattoliniJoaquin ArinoValentina ŠpaničJoaquin Gomis-CebollaValentina TagliettiJoaquín J. SalasValentina VenutiJoaquin Manzo-MerinoValentine ComaillsJoaquín María Campos RosaValentine S. MoulléJoaquina PinheiroValentyn OksenychJochen BaßlerValeria AllizondJochen LauterbachValeria BachioccoJochen MattnerValeria BariliJochen MetzgerValeria CostantinoJochen PölingValeria De MatteisJochen StrubeValeria LallaiJochen WilhelmValeria LeuciJodi HedgesValeria MarigoJoe JamesValeria MericoJoel JamesValeria MerzJoel M. HarpValeria PecceJoel R. DrevetValeria Rachela VillellaJoel T. MagueValeria ScalaJoen LuirinkValeria TaralloJoerg BurgstallerValerian DormoyJohan HenrikssonValerie BolivarJohan HoebekeValérie GaudinJohan LindqvistValerie Gouilleux-GruartJohan P. Van Den HeeverValerie J. CarabettaJohann BauerValérie Jouan-HureauxJohann GoutValerie Le SageJohann HeiderValérie Metzinger-Le MeuthJohann SchredelsekerValerio CaputoJohannes FrankValerio CicconeJohannes HaybaeckValerio FulciJohannes Jakobsen SidelmannValerio MagnaghiJohannes K. EhingerValerio PaoliniJohannes LüttgenauValerio SanguigniJohannes MüthingValerio VolianiJohannes NimpfValeriy VerchenkoJohannes ReichValery AndrushchenkoJóhannes ReynissonValery ZinchenkoJohannes SchlondorffValle PalomoJohannes StadlmannVallì De ReJohannes ZueggValya N. VassilevaJohji KatoValya VassilevaJohn A. D′EliaVance G. NielsenJohn A. St. CyrVance Girard NielsenJohn A. TayekVanesa CepasJohn AndersonVanesa EstebanJohn BarrettVanesa Martín FernándezJohn Chad BrennerVanessa A. MoraisJohn CrispinoVanessa CastelliJohn CuppolettiVanessa D′AntongiovanniJohn D. BoyceVanessa Gil FernándezJohn D. HorowitzVanessa Liévin-Le MoalJohn D. LueckVanessa Morais FreitasJohn De VosVanessa P. HoudeJohn DzimianskiVanessa R. KayJohn ErpeldingVanessa RaymontJohn G. CarmanVanessa VieiraJohn G. LaffeyVanja DuricJohn G. LoganVanja IvkovićJohn GrayVarsha JainJohn HaycockVarun KulkarniJohn HelliwellVarun Kumar KesherwaniJohn Hoon RimVarun Shenoy GangoliJohn HulmeVarvara ValotassiouJohn ImigVasantha-Srinivasan PrabhakaranJohn J. HalperinVasanthi R. SunilJohn J. NemunaitisVasco BrancoJohn J. SkokoVasco SequeiraJohn J. TurchiVasile SimulescuJohn JamesVasileios SiokasJohn Jia En ChuaVasileiou PanagiotisJohn L. ReaganVasilia FasoulaJohn Lalith Charles RichardVasilii Vitalievich PtushenkoJohn Mac SharryVasiliki KalatzisJohn MackrillVasiliki PanagiotakopoulouJohn ManionVasiliki SarliJohn MariadasonVasilios TsarouhasJohn MatsoukasVasily A. PopkovJohn N. HutchinsonVasily KashtalapJohn O. OnukwuforVassilia Ismini AlexakiJohn P. BrooksVassilios PapaloisJohn StanfordVassilios SikavitsasJohn T. BatesVassilis AidinisJohn T. ConnellyVassilis AthanasiadisJohn W. NicholsonVassilis J. DemopoulosJohn W. SteeleVassilis PaschalisJohn WilliamsVassiliy TsytsarevJohnathan NapierVasti T. Juárez-GonzálezJohnny D. FigueroaVeena KochatJohnny Di PierdomenicoVenerando PistaràJoji OkazakiVenkat R. PannalaJolán CsiszárVenkat ReddyJolanta ArtymVenkata MachhaJolanta B. ZawilskaVenkata SabbasaniJolanta Behnke-BorowczykVenkategowda RamegowdaJolanta DorszewskaVenkatesan ThimmakonduJolanta KowalskaVenkatesh Periyakavanam Thiruma-laikumarJolanta LiesieneVenkitasamy BaskarJolanta MierzejewskaVenugopala Reddy GonehalJolanta Opiela‪Venura HerathJolanta RzymowskaVera A. KrischikJolanta SzymańskaVera AmicarelliJolanta TarasiukVera ChernonosovaJolanta WeaverVera FranciscoJoline RozeVera Marisa CostaJon Anthony HyettVera MatveevaJon Del ArcoVera MuccilliJon P. GoldingVera RogiersJon P. WoodsVera TiedjeJonas BystromVera V. MorozovaJonas R. KnudsenVerdiana RavarottoJonas TreebakVerena KohlerJonathan A. LindquistVerena ScheperJonathan AshmoreVerena WieserJonathan BallVernadeth AlarconJonathan ColemanVernell WilliamsonJonathan DickerhoffVernon W. DolinskyJonathan FusiVeronica AlonsoJonathan M. BeckelVeronica AntipovaJonathan P. MillerVeronica AranJonathan PerreaultVeronica BegniJonathan Puente-RiveraVerónica C. MartinsJonathan R. DimmockVeronica DusiJonathan S. LindseyVeronica Elena TrombitasJonathan S. ReichnerVeronica EspositoJonathan SoVeronica Gomez-GilJonathan TurnerVeronica MacchiJonathan TyzackVeronica MartiniJong BhakVerónica Moreno-ViedmaJong Hee LeeVeronica Sanda ChedeaJong Hyuk SungVeronica VellaJong Jin OhVeronica VeschiJong KimVeronica WileyJong Kook ParkVeronika AleksandrovychJong Kwan ParkVeronika HuntosovaJong Pil ParkVeronika HyskovaJong-Eun KimVeronika Kralj-IglicJongho JeonVeronika Lukacs-KornekJonghyun OhVeronika StokaJong-Jer LeeVéronique BergougnouxJongki ChoVéronique BroussolleJongmin KimVéronique CadoretJong-Seok MoonVéronique KemmelJoni H. YlostaloVéronique StovenJoo Mi YiVersha BanerjiJoo Young HuhVesna JacevicJoon W. ShimVesna KosecJoong Hyoun ChinVesna PeršićJoong-Hyuck AuhVesna Tumbas ŠaponjacJoost Van Der VorstVesna VasicJoost WillemseVesna VucicJoo-Won JeongVesta SteiblieneJoradan PatikViacheslav NikolaevJordi AlcarazVibe Hallundbæk OestergaardJordi MiroVibhor MishraJordi MolgóVicente AndreuJordi Pérez TurVicente Espinosa-SolisJordi PuiggalíVicente Gotor-FernándezJordi Querol AudíVicente MartinezJordi TamaritVicente Seco-RoviraJörg BernhardtVickery Trinkaus-RandallJörg D. HoheiselVíctor A. Lórenz-FonfríaJörg FitterVictor Bocos-BintintanJörg KleeffVictor ChongJörg LehmannVíctor De La Asunción NadalJörg PetersVictor F. PlyusninJörg PieperVictor GarciaJörg ScheffelVíctor García-GonzálezJörg WaglerVictor GonzalezJorge BecerraVictor HernandezJorge CanhotoVictor HsiaJorge Eduardo Aedo AbadieVictor HuberJorge EstévezVictor Ivanovich SeledtsovJorge FeitoVictor J. CidJorge Galindo-VillegasVictor J. YusteJorge GonçalvesVictor LamzinJorge Gonzalez-GarciaVíctor M. Sánchez-PedregalJorge H. LeitãoVíctor Manuel Navas-LópezJorge LopezVíctor Quesada PérezJorge LoraVictor RomanovJorge M. A. OliveiraVíctor Ruiz-Valdepeñas MontielJorge M. Alves-SilvaVictor TatarskiyJorge M. B. VítorVictor Van BeusechemJorge Morales-MontorVictor YuJorge Moreno CharcoVictoria BunikJorge OliveiraVictoria CachofeiroJorge Rodriguez-GilVictoria Del PozoJorge Santos-JuanesVictoria LawsonJorge Verdú-AndrésVictoria McGilliganJørn Bolstad ChristensenVictoria PastorJorrit M. EnserinkVictoria SevostyanovaJorunn BörveVictoria V. MironovaJosanne VassalloVictoria V. SevostyanovaJosé A. CañasVictoria ValdiviaJosé A. Godoy-LugoVida StrasserJosé A. González-PérezVidas RaudonisJosé A. MartinsVidmantas BendokasJose A. TapiaVidula VachharajaniJosé AndradeVidyalakshmi SethunathJosé Ángel ArmengolVidyani SuryadevaraJose Angel FontenlaViera KovacovaJosé António BeloViera SchwarzbacherováJosé Antonio Fernández-LópezVigdis AasJosé Antonio López-GuerreroVijai Kumar Reddy TangadanchuJose Antonio MolerVijay AvinJosé Antonio Morales-GonzálezVijay KumarJosé Antonio PalenzuelaVijay Kumar SiripuramJosé Antonio PellicerVijay Kumar ThakurJosé Antonio PintoVijay ParasharJosé Avendaño-OrtizVijay RamakrishnanJosé Bernardo Noronha MatosVijay Sagar MadamsettyJosé Blanco SalasVijay VyasJose CanasVijaya KaroorJose Caparros-MartinVijayabhaskar VeeravalliJosé Cleiton Sousa Dos SantosVijayakumar SukumaranJose CornejoVijayalaxmi G. GuptaJosé DiasVikas SaxenaJose DieVikash Kumar YadavJosé Dijair AntoninoVi-Khanh TruongJose Emanuel Loeza AlcocerVikram K. MulliganJosé Enrique Torres VaamondeVikram MulliganJosé F. Ruiz PérezVikram PrasadJosé Felipe Warmling SprícigoVikram SharmaJosé Félix De CelisVikramjit S. BajwaJosé Fernando FierroVikrant RaiJose Garcia De La TorreViktor I. SevastianovJosé Ginés Hernández-CifreViktor Korzhikov-VlakhJosé Guadalupe Soñanez-OrganisViktor PilepićJosé H. TeixeiraViktor V. RevinJose J. G. MarinViktor VíglaskýJose Javier FusterViktoria JeneyJosé Javier Zamorano-LeonViktória JeneyJose L. Gómez-UrquizaViktoria MilkovaJose L. HuesoViktorie VlachovaJosé Luis AffranchinoVilborg PalsdottirJosé Luis Gómez-ArizaVilceu BordignonJose Luis LavanderaVilma PetrikaiteJose Luis Pérez-CastrillónVilma Yuzbasiyan-GurkanJose Luis Rodriguez-FernandezVinay KumarJosé Luis ZugazaVinay ShuklaJose M. LizcanoVinaya ManchaiahJose M. LorenzoVinayaga Srinivasan GnanapragassamJose M. MuletVinayak C. PalveJosé M. Muñoz-FélixVincent Blasco-baqueJosé M. Pérez-OrtizVincent ChaptalJose M. PrietoVincent DelormeJosé M. Rodrigo-MuñozVincent GiguereJosé Manuel BaizabalVincent HascallJose Manuel BreaVincent PiccoJosé Manuel Pérez De La LastraVincent Picher-MartelJosè Manuel PionerVincent ReyesJose Manuel Rodriguez-VargasVincent YeungJose Manuel Villalobos EscobedoVincenza BarresiJosé Marco-ContellesVincenza GianfrediJosé María González-GranadoVincenzo AlterioJose María RequenaVincenzo CarafaJosé Miguel GuzmánVincenzo CavalieriJose Miguel SorianoVincenzo Cristian FrancavillaJosé Muñoz EspaderoVincenzo De FilippisJosé P. Cerón-CarrascoVincenzo De LeoJosé PalmaVincenzo Di StefanoJosé Pedraza ChaverriVincenzo L′ImperioJosé Pedro Pinto De AraújoVincenzo LionettiJosé Peña-AmaroVincenzo MarottaJose PerdomoVincenzo MatteiJose PereiraVincenzo MitoloJosé Pérez-RigueiroVincenzo MollaceJosé PinelaVincenzo Nicola TalesaJose Prieto GarciaVincenzo PavoneJose R. BayascasVincenzo PezziJosé Rafael De AlmeidaVincenzo PotaJosé Ramón González-PorrasVincenzo QuagliarielloJose Ramon PinedaVincenzo ScarlatoJosé RiveraVincenzo TufarelliJose TudelaVincenzo VindigniJosef ElsterVineela ParvathaneniJosef HeckmannVineeta TanwarJosef JůzaVinicius AlvesJosef Stolberg-StolbergVinit RajJosefa Muñoz AlamilloVinit ShanbhagJosefina AleuVinita ChittoorJosefina PonsVioleta MelinteJosene Maria ToldoVioleta S. RilchevaJosep JulveVioletta MacioszekJosep M. Cambra BortVioletta Opoka-WiniarskaJoseph (Sifis) PapamatheakisViorica Railean-PlugaruJoseph A. CurranVipin Chandra KaliaJoseph AllisonVirag VasJoseph BorgVirender SinghJoseph Calvin KouokamVirginie DoceulJoseph John KochmanskiVirginie EscabasseJoseph K. BelanoffVirginie LibanteJoseph LorentVirginie MarcelJoseph M. DragavonVirginie MariotJoseph MarcotrigianoVirginie Prulière-EscabasseJoseph MarinoVirginie RappeneauJoseph O. Falkinham IIIVirginie TardifJoseph PancrazioVirginija JankauskaiteJoseph R. PetersonVishal JaikaransinghJoseph ZaiaVishal M. KothariJosephina MeesterVishnu D. RajputJosephine FerreonVishnu Priya MuraliJosephine S. Modica-NapolitanoVishnu RevuriJoshua D. KleinVishnu Sukumari NathJoshua M. KaplanVishwaraj SontakeJoshua OoiVita GolubovskayaJoshua ScallanVitali SikirzhytskiJoshua T. MorganVitaly A. SorokinJoshua V. VermaasVitaly KiselevJosiah OchiengVitaly V. KushnirovJosie ParkerVito D′AndreaJosimar De Oliveira EloyVito Giuseppe D′AgostinoJosip A. BorovacVito TerlizziJosipa VlainićVittoria CammisottoJosko BozicVittoria CenniJoško OsredkarVittoria ColottaJosselin LupetteVittoria Di MauroJosy AugustineVittorio AprileJovan PesovicVittorio BianchiJowy Yi Hoong SeahVittorio GentileJoydeep BhaduryVivek AdvaniJózef HaponiukVivek GargJózef J. BujarskiVivek ManivelJózsef DeliVivian A. GuedesJozsef DudasVivian BernauJozsef FodorVivian De WaardJózsef TímárVivian K. O′DonnellJózsef TőzsérViviana BarraJr-Jiun LiouViviana CordedduJu Hyun ParkViviana MeravigliaJuan A. BernalViviana TriacaJuan A. Fafián-LaboraVivienne ReeveJuan A. PargaVjekoslav PeitlJuan AyalaVlad PădureanuJuan Bautista De SanctisVlad TomaJuan Carlos AlonsoVlada PenevaJuan Carlos Del PozoVladan ČokićJuan Carlos DominguezVladan OndřejJuan Carlos GardónVladimir A. TimoshevskiyJuan Carlos LagunaVladimir BabenkoJuan Carlos LedesmaVladimir BaulinJuan Carlos Rodríguez-ManzanequeVladimir ChubanovJuan E. Palomares-RiusVladimir ChulanovJuan Francisco ArandaVladimir DyakonovJuan GalloVladimir I. ArkhipovJuan I. Pérez-CalvoVladimir I. KalininJuan J. FreireVladimir IsachenkoJuan José Martínez NicolasVladimir KashoJuan José ParajoVladimir KefalovJuan José Ramos-RodríguezVladimir Mulens-AriasJuan LiVladimir MuronetzJuan M. Fernández-CostaVladimir NikolenkoJuan Manuel Gonzalez RosaVladimir O. PustylnyakJuan Mozas-MorenoVladimir PacutaJuan Pablo NicolaVladimir PalyulinJuan Pablo RigalliVladimir RudajevJuan ParisVladimír ScholtzJuan PerdomoVladimir SerezhenkovJuan Pérez-FernándezVladimir StevanovićJuan R. Muñoz-CastañedaVladimir SukhorukovJuan Sanchez GurmachesVladimir TesarJuan TorrasVladimir TominJuan Vicente LucianoVladimir TorbeevJuan VillenaVladimir UverskyJuana Serrano LopezVladimir V. KuznetsovJuanita AndersVladimir V. SharoykoJudit CastilloVladimir ZhabinskiiJudit MarsillachVladimir ZhemkovJudit SymmankVladimirs PilipenkoJudit VárkonyiVlastimil KuldaJudite N. BarbosaVoahanginirina RandriamboavonjyJudith DavieVojislava BursicJudith KassisVolker M. LauschkeJudith Klein-SeetharamanVolker MüllerJudith MihályVolkhard HelmsJudith OgilvieVolodymyr RadchukJudith PetersVolodymyr ShvadchakJudith StaerkVolodymyr TryndyakJudith Van HoutenVolodymyyr KravetsJudy CallisVonk LucienneJue HouVoula KanelisJuei-Tang ChengVrba JiriJuexin WangVsevolod V. GurevichJu-Fang LiuVui King Vincent-ChongJugal Kishore SahooVyacheslav YurchenkoJuha P. VäyrynenVytaute StarkuvieneJu-Hee KangW. D. GrimmJuin-Hong CherngW. Rudolf SeitzJui-Yen HuangW. Ted AllisonJuli UnternaehrerWaheed ShabbirJulia Christina GrossWai Keung LeungJulia CostaWai Kit ChuJulia EtichWai KwokJulia GauerWakako FujitaJulia LorenzoWakiro SatoJulia MetzgerWalace Gomes-LealJulia Pereira LemosWalburgis BrennerJulia RomanoWaldemar GoldemanJulia SchuelerWaldemar GrzegorzewskiJulia StadlerWalee ChamulitratJulia VainonenWaleska C. DornasJulia WeissWalid AzabJulián AragonésWalid El KayalJulian ChojnowskiWalter CaseriJulian ChristiansWalter FiedlerJulian Freen-van HeerenWalter G. BradleyJulian HillyerWalter MancinoJulian RozenbergWalter WahliJuliana CotabarrenWan LeeJuliana M. RosaWanda LattanziJuliane LokauWang JiaweiJuliane MüllerWanting NiuJuliane Salbach-HirschWanwen ZengJuliann G. KiangWard De SpiegelaereJulianna KardosWarren HeidemanJulianne C. GriepenburgWaruna DissanayakaJuliano Vilela AlvesWasim H. ChowdhuryJulie A. Sterling RhoadesWassim El-NemerJulie BejoyWasu Pathom-areeJulie Brogaard LarsenWataru KamitaniJulie DaviesWataru MiyazakiJulie E. SimpsonWataru NishimuraJulie FoxWayne Brian HunterJulie GrahamWayne CarverJulie Guillermet GuibertWayne GilesJulie RageulWayne RobertsJulie TuckerWayne ThomasJulie Y. JiWayne XuJulien ChapuisWei CuiJulien CurabaWei KangJulien DairouWei LeiJulien P. DupuisWei LiJulien PernierWei LiuJulien RossignolWei ShaoJulien Saint-PolWei ShenJulien TailhadesWei SunJulien VignardWei WeiJuliet MorrisonWei WuJuliette E. MoreauWei YaoJulija VolmajerWei ZhangJulio ÁvilaWei ZhongJulio Bastos-ArrietaWei-Biao LiaoJulio DelgadoWeibing YangJulio EscribanoWeichen HuangJulio MoralesWei-Cheng LoJulita KulbackaWei-Cheng TsengJulita PiaseckaWei-chieh ChengJulita RegułaWei-Chieh LeeJulius MoratinWei-Chyou WeiJumpei FujikiWei-Hsuan YuJumpei SaitoWeihua QinJun HamazakiWeihua TianJun ImaiWeijie ZhangJun InoueWei-Li ChenJun KobayashiWeilue HeJun KunisawaWei-Min ChangJun MiyakeWeiming LiJun MiyataWei-Ning LinJun MoritaWeiqin LuJun OhnoWeiran ShanJun TeishimaWei-Shiung LianJun ToyoharaWeiyan YinJun UedaWei-Yi LinJun WangWen Chieh LiaoJun Young HeoWen Hai ShaoJun ZhouWenbin TuoJunaith S. MohamedWenchao YangJunchao TongWen-Cheng ChenJune Ereño-OrbeaWen-Chin LeeJune-seok HeoWen-Chung HuangJune-Sun YoonWendell D. JonesJunfeng MaWen-Der WangJunfeng WangWendy BollagJung Eun KimWendy L. ImlachJung Ryeol LeeWendy S. SmithJung Su RyuWen-Fu LaiJung Sun YooWen-hong LiJung-Hwa Tao-ChengWen-Jen LeeJunghyun KimWenjia WangJungil ChoiWenjing PengJung-suk SungWen-Juan MaJungwon LeeWenjun ZhangJungwoo LeeWen-Lii HuangJungwoon LeeWen-Ling LiaoJun-Hong LiuWen-pin HuJunhua YangWen-Pin SuJunichi HanaiWensheng WangJunichi OmuraWenshih TsaiJun-ichi SawadaWen-Ta SuJunichi ShinozakiWen-tao DengJunichi TakinoWenting DuJunji FuruseWen-Wei ChangJunji KawakamiWen-Wei SungJunji TakayaWenwen JingJunjiang SunWenyan XuJunjie LiWern-Joo SohnJunken AokiWeronika Dymara-KonopkaJunki SakataWeronika RzepnikowskaJunko KurokawaWesley Raup-KonsavageJunmo KimWhitney A. BullockJun-O JinWhitney LongmateJunqi SongWibke BayerJun-Ren SunWidiastuti SetyaningsihJunsei MimuraWieslaw BarabaszJunsoo LeeWiesław BielasJunsoo ParkWiesław KacaJunwei ShiWieslaw SwietnickiJunxi WuWieslaw ZiolkowskiJunya TanakaWieslawa KrancJunyan TaoWiktor JedrzejczakJuozas LazutkaWiktor PaskalJu-Pi LiWilber Romero-FernandezJuraj MokrýWilfredo De Jesús-RojasJuraj PiestanskyWilfried Le GoffJūratė SkerniškytėWilfried RozhonJure KnezWilhelm K. AicherJürg NüeschWill BailisJürgen BartelWilliam A. LaingJürgen BrosiusWilliam A. McEwanJürgen J. HeinischWilliam B. MillerJuscelino TovarWilliam BlalockJustin A. RobyWilliam Brad O′DellJustin ConleyWilliam Bryan TerzaghiJustin H. J. NgWilliam Burgess GrantJustin HsuanWilliam C. MerrickJustin HwangWilliam CamuJustin J. GreenleeWilliam Chi-Shing TaiJustin P. BlumenstielWilliam ConnellyJustin RenaudWilliam DuddyJustyna E. GołȩbiewskaWilliam G. MayhanJustyna GodosWilliam G. WillmoreJustyna HermanowiczWilliam H. WalkerJustyna Karkowska-KuletaWilliam HuckleJustyna KozłowskaWilliam J. JeffcoateJustyna MilcWilliam JacotJustyna MiszczykWilliam K. ChanJustyna StruzikWilliam McBrideJustyna SzerementWilliam MoarJustyna WidomskaWilliam N. HaJustyna ZińczukWilliam N. MwangiJuta KroičaWilliam N. SetzerJutiporn ThussagunpanitWilliam TerzaghiJutta Ludwig-MüllerWillibald WonischJutta PapenbrockWilly BienvenutJu-Yang JungWilma BarcelliniJu-Yi ChenWilman CarrilloJuzoh UmemoriWim DeclercqJwakyung SungWim Van Den EndeJya-Wei ChengWinfried L. NeuhuberJyh-Cherng JuWin-Long ChiaJyh-Shiarn CherngWioleta GrabowskaJyh-Yeuan LeeWioleta KowalczykJyotirmoy DasWioletta BielK. G. PrasanthWioletta Ewa PluskotaK. KisandWioletta SamolińskaK. KurodaWioletta Szczurek-WasilewiczK. Scott WeberWirginia Kukula-KochK. V. KiselevWisit KaewputKa Fung Peter ChiuWisnu Adi WicaksonoKa L. HongWissam FarhatKa Yee FungWissem MnifKaalak ReddyWitold JakubowskiKaare GautvikWitold SzaflarskiKabirullah LutfyWitold WachowiakKacper DruzbickiWladimiro JiménezKah Kheng GohWladyslaw JanusKah-Hui WongWładysław LasońKai Alexander DünserWlodzimierz A. StańczykKai CaiWojciech BikKai MasurWojciech CiszewskiKai P. HoefigWojciech DąbrowskiKai WangWojciech JelskiKai YangWojciech Jerzy PietrońKai ZhangWojciech KalasKai-Che WeiWojciech KamyszKai-Lee WangWojciech KochKaito TakahashiWojciech Michał CiszewskiKai-Wen HsuWojciech PaslawskiKaja Blagotinšek CokanWojciech PiekoszewskiKajetan GrodeckiWojciech SmułekKalaska BartlomiejWojciech WitkiewiczKa-Leung WongWolfgang BlenauKalidou NdiayeWolfgang E. BerdelKalina SzteynWolfgang EisenreichKállai NikolettWolfgang SattlerKálmán KönyvesWolfgang SchadenKalpana GhoshalWolfgang SchweigkoflerKalpana RajaWolfgang WeigandKalpita BanerjeeWolfram KunzKalra Rajkumar SinghWon Hee LeeKalyan SaginalaWon Kyong ChoKamakoti BhatWon Min ParkKamal A. M. Abo-ElyousrWonchung LimKamal JnawaliWoojin KimKamalendra SinghWoong-Hee ShinKamalika MojumdarWooyeong ParkKamayani SinghWuletaw Tadesse DeguKamel Abd-ElsalamWupeng LiaoKamel Ahmed Abd-ElsalamXi ChenKameyama ToshikiXiangmeng QuKamil JastrzębskiXiangping ChuKamil KaminskiXiangru TangKamil KostynXianjun PengKamil KrawczynskiXiaobin HuangKamil RomanXiaohong WangKamil RůzǐčkaXiaolin (Steven) CuiKamil WojciechowskiXiaoping PuKamila Kasprzak-DrozdXiaoyan ChenKamila ŘasováXiaoyan QiuKamilla StachXiaozhuo ChenKamlesh MakwanaXijun PiaoKamol DeyXinbo LiKamrun NaharXing LiuKamyar AsadipooyaXingguo ChengKan SaitoXisha WangKanai MasatakeXiuquan MaKanako Bessho-UeharaXuan LiKanate ThitiananpakornXudong HuangKanchan VishnoiXue-Hai LiangKandhasamy SowndhararajanXun ChenKang-Mo KuXuqiao ChenKaniz Fatima Binte HossainYa-Chung TianKankan WangYahli LorchKannan MuthukumarYamaoka ToshimitsuKaori FujitaYana CenKaori SakoYana J. AnfinogenovaKapfhammer JosefYanfei (Jacob) QiKar Wey YongYang Jae KangKarel DoležalYang LiKarel KotaškaYang Wen-ChiKarel KrálovecYan-Jye ShyongKarel SakslYanlun ZhuKarel SedlarYan-Ming XuKarel SmetanaYaroslav AndreevKaren A. BoehmeYarra RajeshKaren BoazYasemin M. AkayKaren CooperYasser HeakalKaren CrawfordYasuhiko KatoKaren MolyneuxYasuhiko KizukaKaren R. JonscherYasuhiro KubotaKaren SeiterYasuhiro KuroiKaren SkriverYasuhiro MikiKaren Wing Yee YuenYasuhiro OzekiKaren WrightYasuhito IshigakiKari KopraYasuhito ShiraiKari L. StoneYasuko OnoKari NaylorYasuo TerauchiKarim Alexandre AbidYasushi SasaiKarim HamaouiYasushige ShinguKarim HameschYasutaka ShigemuraKarima DjabaliYasuyuki FukuharaKarin BuschYau Kei ChanKarin RobergYaw Sing TanKarina Kubiak-OssowskaYa-Xiong TaoKarina SalekYayoi KobayashiKarina ShimizuYelena Valentinovna LikhoshwayKarine GoussetYen-Chien LeeKarine PageauYeng-Tseng WangKarl BayerYen-Wen LiuKarl Heinz KlempnauerYeon Soo HanKarl Henrik GrinnemoYeong Deuk JoKarl HerholzYeon-Sun SeongKarl J. SchreiberYeri RimKarl Kai McKinstryYevgeniya I. ShuruborKarl KunzelmannYi-Fan ChenKarl LintnerYihao ChenKarl RobinsonYih-Fung ChenKarl StraubYi-Hsien HsiehKarl TsimYi-Hsuan HsiaoKarl-Heinrich LinkYih-Wen WangKarl-Michael SchebeschYin-Chih FuKarmela BarisicYing-Hsien KaoKarmella HaynesYingleong Rigel ChanKarol CiepluchYino WuKarol FijałkowskiYi-Ping YangKarol SikoraYi-Shiuan LiuKarolina A. Wojtunik-KuleszaYi-Ting LinKarolina Anna Wojtunik-KuleszaYi-Ting YenKarolina BoguszewskaYohei IkezumiKarolina DrężekYohei SekinoKarolina KotYoichi EzuraKarolina KowalskaYoichiro MitsuishiKarolina LudwickaYong Qin KohKarolina M. StepienYong-Hwan MoonKarolina NowakYongming BaoKarolina PytkaYong-Moon LeeKarolina Skonieczna-ŻydeckaYongseok JeeKarolina SzczepanowskaYosef FichmanKarolina Szewczyk-GolecYoshiaki KitamuraKarolina WieszczyckaYoshihiko UsuiKarolina WoronieckaYoshihiro FukumotoKaroly HebergerYoshihiro HottaKarsten KnoblochYoshihiro ShidojiKarsten MelcherYoshihiro TaguchiKarsten PlotzYoshihisa KaizukaKarthik UppuluryYoshikazu KitanoKartik AnandYoshinari UeharaKartik BeheraYoshinori ArisakaKasahara KotaYoshinori HayashiKasandra BélangerYoshiro HoraiKasavajhala PrasadYoshiro ItataniKashmir SinghYoshitaka HosokawaKassem MakkiYoshitoshi KasuyaKassoum NacroYoshiyu TakedaKasturi Joshi-NavareYoshiyuki TatsumiKasturi PalYoshizumi IshinoKasturi RangannaYou LuKatalin LumniczkyYoung Ran KimKatalin SiposYoung UhKatalin SzásziYoung-Dan ChoKatarina KluckovaYoungjun MoKatarína RažnáYoung-Kwon SeoKatarina SmiljanićYoung-Ok SonKatarina VukojevicYoungsoo ParkKatarzyna A. PirógYousef NaserzadehKatarzyna Adamczewska-SowińskaYu Ah HongKatarzyna Baldy-ChudzikYu LiuKatarzyna Cieślik-BoczulaYu SekiguchiKatarzyna CzarzastaYu ToyodaKatarzyna DudaYuan HuKatarzyna Gaweł-BębenYuan-Hsi WangKatarzyna GolanYu-Chieh LiaoKatarzyna Hnatuszko-KonkaYue QiKatarzyna HojanYue ShengKatarzyna IżykowskaYuen ClementKatarzyna KaczyńskaYu-Fang ShenKatarzyna Kiec-KononowiczYuhei TakadoKatarzyna KirszYuh-shuen ChenKatarzyna KodziszewskaYu-Hsiang KuanKatarzyna Komosinska-VassevYuichi TogashiKatarzyna KorybalskaYuichi TsuchiyaKatarzyna KowalczykYuichiro SatoKatarzyna KowalskaYu-Jen ChenKatarzyna KoziakYuji HirowatariKatarzyna KubiakYuji KamatariKatarzyna KujawaYujiang FanKatarzyna KuterYu-Jung ChengKatarzyna KwiatkowskaYuki ManabeKatarzyna ŁudzikYuliya I. RaginoKatarzyna M. LisowskaYuliya KhrunykKatarzyna Marciniak-ŁukasiakYulong TangKatarzyna MichalekYumiko MotoiKatarzyna NucYung-An TsouKatarzyna OtulakYung-Shun JuanKatarzyna Otulak-KoziełYunhi ChoKatarzyna PalusYunkyoung LeeKatarzyna Patrycja SzymańskaYun-Qing LiKatarzyna PentośYuri SergeevKatarzyna Pietraszek-GremplewiczYuri V. SergeevKatarzyna PiórkowskaYuriy OrlovKatarzyna PiotrowskaYusof KamisahKatarzyna PlacekYusuke KatoKatarzyna PobiegaYusuke OshimaKatarzyna PogodaYuta HatoriKatarzyna PotrykusYuto UedaKatarzyna ReczyńskaYves HenryKatarzyna Ropka-MolikYvonne BörgelingKatarzyna RoszekZachary F. BurtonKatarzyna SkrypnikZaid MaayahKatarzyna SocałaZamora Martínez Ana MaríaKatarzyna StarowiczZbârcea Cristina ElenaKatarzyna StaszakZbigniew SamochockiKatarzyna Sykłowska-BaranekZdenek HodnyKatarzyna WalczakZdenek WimmerKatarzyna WalkiewiczZehuan LiaoKatarzyna WińskaZhao ChuntaoKatarzyna Winsz-SzczotkaZhen QiuKatarzyna Wójcik-PszczołaZhenghan WangKatarzyna Wolny-KoładkaZhengwu PengKatarzyna ZawadaZhengxin LiKatarzyna ZawadzkaZhi-Fu WuKatarzyna ZorenaZhihao ChenKate R. SecombeZhipeng LiuKate SutherlandZhiwei YeKateřina KaňkováZhong ZhengKaterina KuzelovaZhong-Ru XieKaterina P. SkenderiZhoufei WangKateřina PetříčkováZhu KeyingKaterina PsarraZibing JinKaterina StratiZihao OuKateřina ValentováZippora (Zippi) BrownsteinKatharina A. ScherfZiru LiKatharina RichterZiwen JiangKatharina RumpZiwen WangKatharina TimperŽofie SovováKatherine H. IngramZograb MakiyanKatherine R. HixonZoltan BozsoKatherine SchultheisZoltán DeimKatherine WitrickZoltán HeroldKathleen A. R. SchildrothZoltan JakusKathleen D. CusickZoltan MagyarKathleen HaleyZoltán PethőKathleen Hering-SmithZoltán VargaKathleen Laura HefferonZoltán WienerKathleen PappritzZongchao JiaKathrin EngelZsófia BánfalviKathryn E. ColeZsolt BacsoKathryn Y. BurgeZsolt SarangKatia CorteseZsolt SarszegiKatia LeiteZsuzsa SzondyKatie AldredZsuzsanna Bata-CsorgoKatie FrenisZsuzsanna LichnerKatie SheatsZuzanna BielanKatiuscia PaganoZygmunt WarzechaKatja Eckl


